# Scaffold Hopping in Discovery of HIV-1 Non-Nucleoside Reverse Transcriptase Inhibitors: From CH(CN)-DABOs to CH(CN)-DAPYs

**DOI:** 10.3390/molecules25071581

**Published:** 2020-03-30

**Authors:** Ting-Ting Li, Christophe Pannecouque, Erik De Clercq, Chun-Lin Zhuang, Fen-Er Chen

**Affiliations:** 1 Institute of Pharmaceutical Science and Technology, Zhejiang University of Technology, Hangzhou 310014, China; toplis163@163.com; 2 Rega Institute for Medical Research, KU Leuven, Herestraat 49, B-3000 Leuven, Belgium; christophe.pannecouque@kuleuven.be (C.P.); erik.declercq@kuleuven.be (E.D.C.); 3 Department of Chemistry, Fudan University, Shanghai 200433, China; 4 Shanghai Engineering Center of Industrial Asymmetric Catalysis for Chiral Drugs, Shanghai 200433, China

**Keywords:** CH(CN)-DAPYs, HIV-1, NNRTI, scaffold hopping, strucutral optimization

## Abstract

Scaffold hopping is a frequently-used strategy in the development of non-nucleoside reverse transcriptase inhibitors. Herein, CH(CN)-DAPYs were designed by hopping the cyano-methylene linker of our previous published CH(CN)-DABOs onto the etravirine (ETR). Eighteen CH(CN)-DAPYs were synthesized and evaluated for their anti-HIV activity. Most compounds exhibited promising activity against wild-type (WT) HIV-1. Compounds **B4** (EC_50_ = 6 nM) and **B6** (EC_50_ = 8 nM) showed single-digit nanomolar potency against WT HIV-1. Moreover, these two compounds had EC_50_ values of 0.06 and 0.08 μM toward the K103N mutant, respectively, which were comparable to the reference efavirenz (EFV) (EC_50_ = 0.08 μM). The preliminary structure–activity relationship (SAR) indicated that introducing substitutions on C2 of the 4-cyanophenyl group could improve antiviral activity. Molecular docking predicted that the cyano-methylene linker was positioned into the hydrophobic cavity formed by Y181/Y188 and V179 residues.

## 1. Introduction

Human immunodeficiency virus-1 (HIV-1) non-nucleoside reverse transcriptase inhibitors (NNRTIs) are key components of highly active antiretroviral therapy (HAART) for treating acquired immune deficiency syndrome (AIDS) [1]. Over the past decades, more than fifty structurally diverse classes of compounds have been reported as NNRTIs, which can be divided into the following categories according to their structures: TIBO (tetrahydroimidazobenzodiazepinone), α-APA (α-anilinophenylacetamide), ITU (iminothiourea), DABO (dihydro-alkoxy-benzyl-oxopyrimidine), DATA (diaryltriazine), DAPY (diarylpyrimidine), etc. [2,3]. Diarylpyrimidine (DAPY) derivatives are second-generation NNRTIs with remarkable anti-HIV-1 activity and favorable pharmacological properties [4]. Two representative DAPY analogues, etravirine (ETR) and rilpivirine (RPV), were successfully approved by the U.S. Food and Drug Administration (FDA) and are widely used in the clinic setting [5,6,7]. Although RPV versus some NNRTIs exhibited high activity, there are some issues such as high toxicity, poor water solubility, and low bioavailability for DAPY compounds used in clinical practice [8,9,10]. Therefore, further optimization of DAPYs is still a the research focus in the medicinal chemistry community.

**Figure 1:**
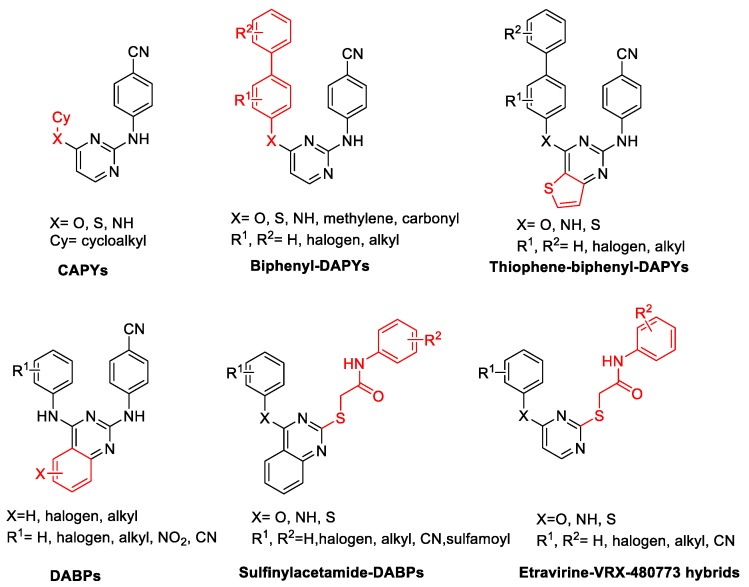
Representative non-nucleoside reverse transcriptase inhibitors (NNRTIs) obtained by the scaffold hopping strategy.

The scaffold hopping strategy is widely applied in the development of NNRTIs. In the past 20 years, our group published about multiple hybrids developed by the scaffold hopping strategy (Figure 1) [11], such as cycloalkyl arylpyrimidines (CAPYs) [12], biphenyl-diarylpyrimidines (Biphenyl-DAPYs) [10,13], thiophene-biphenyl-DAPYs [7,14], diarylbenzopyrimidines (DABPs) [15], sulfinylacetamide-DABPs, and etravirine-VRX-480773 hybrids [16,17,18]. As a result, these compounds exhibited remarkably increased antiviral activity compared with their parent lead compounds, indicating the efficacy of the hopping strategy in developing the anti-HIV drug candidates.

**Figure 2:**
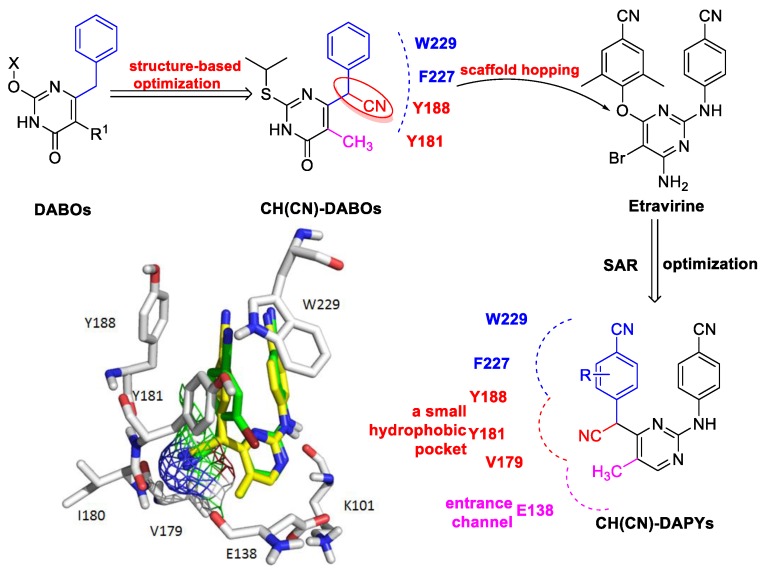
Molecular design of CH(CN)-DAPYs.

The dihydro-alkoxy-benzyl-oxopyrimidines (DABOs), first reported in 1992, were recognized to be potent anti-HIV compounds. In our group, several potent analogues were obtained by structure-based drug design (SBDD). In 2007, a cyano group was introduced into DABO as a linker to obtain a series of novel CH(CN)-DABOs (Figure 2) with superior antiviral activity against wild-type (WT) HIV-1 (LAI strain III_B_) (EC_50_ = 2 nM). Molecular docking results showed that the 6-(α-cyanobenzyl) moiety of CH(CN)-DABOs fitted into the aromatic-rich non-nucleoside binding pocket, surrounded by the aromatic side chains of Y181, Y188, F227, and W229 [19]. In addition, the cyano group can also form hydrophobic interactions with hydrophobic protein residues [20] together contributing to the high potency.

Given that the oxygen linker of ETR is located at a small hydrophobic pocket formed by Y181/Y188 and V179, we continued with this study to design a new series of DAPY derivatives with the cyano-methylene linker of CH(CN)-DABOs onto the ETR, employing the scaffold hopping strategy. Furthermore, introducing a methyl into the pyrimidinyl ring that is located at the entrance channel containing hydrophobic residues might establish nonpolar interactions with a reverse transcriptase (RT) enzyme (Figure 2) and improve the activity [21,22].

## 2. Results and Discussion

### 2.1. Chemistry

**Scheme 1:**
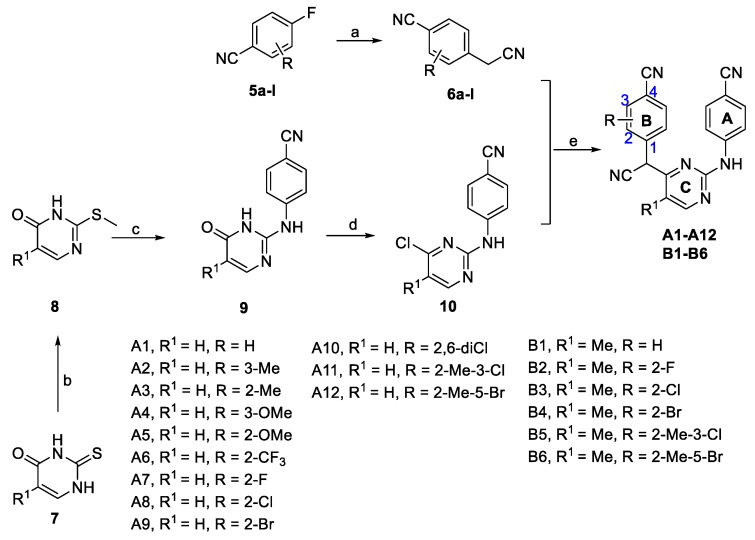
Reagents and conditions: (**a**) methyl cyanoacetate, 60% NaH, DMSO, 90 °C; then H_2_O, reflux; (**b**) MeI, NaOH, H_2_O, r.t., 24 h; (**c**) 4-cyanoaniline, 180–190 °C, 8 h; (**d**) POCl_3_, reflux, 30 min; (**e**) substituted-4-cyanophenylacetonitrile, 60% NaH, N_2_, anhydrous DMF, −15 °C to r.t., 48–72 h.

The synthetic route of the target compounds **A1**–**A12** and **B1**–**B6** are depicted in Scheme 1. The key intermediates 4-(4-chloro-pyrimidin-2-ylamino)benzonitriles (**10**) were prepared from compound (**7**) according to our previously reported three-step protocol [23]. The substituted 4-cyanophenylacetonitriles **6a-l** were synthesized via a one-pot procedure from **5a-l**. Then, compounds **6a-l** were reacted with key intermediates (**10**) to yield the target CH(CN)-DAPYs.

### 2.2. Antiviral Activity of Compounds ***A1***–***A12*** and Binding Conformation Analysis

First, we synthesized the CH(CN)-DAPY analogues (**A1**–**A12**) with methyl, methoxy, trifluoromethyl, and halogen groups on the 4-cyanophenyl group. Their anti-WT HIV-1 activity were evaluated and nevirapine (NVP), etravirine (ETR), efavirenz (EFV), and rilpivirine (RPV), four drugs currently used in clinical treatment of HIV-1 infection, were selected as reference compounds. As illustrated in Table 1, the target compounds **A1**–**A12** exhibited significantly different antiviral activity against WT HIV-1 (LAI strain III_B_) with EC_50_ values ranging from 0.059 to 11.74 μM. Compound **A2–A5** exhibited low cytotoxicity with CC_50_ values ranging from 10.9 to 78.4 μM. Compound **A1** with 4-CN exhibited anti-HIV-1 activity with an EC_50_ value of 3.27 μM. Compound **A2** with 3-Me-4-CN exhibited anti-HIV-1 activity with an EC_50_ value of 1.17 μM. Compared with **A2**, the antiviral activity was 17-fold increased when the methyl substitution was moved to the 2-position (**A3**) of the 4-cyanophenyl moiety. Compound **A3** with 2-Me-4-CN displayed potency against WT HIV-1 with an EC_50_ value of 0.069 μM and low cytotoxicity with a CC_50_ of 10.9 μM. Replacing the methyl by methoxy (**A4**) made the activity decrease to an EC_50_ of 11.74 μM. Similarly, the 2-methoxy analogue (**A5**) had a 200-fold higher activity than the 3-substituted analogue (**A4**). Compound **A5** with 2-OMe-4-CN displayed potency against WT HIV-1 with an EC_50_ value of 0.059 μM. Furthermore, compound **A5** exhibited low cytotoxicity with a CC_50_ of 25.8 μM, which was about 5-fold higher than that of RPV (CC_50_ = 5.9 μM). These results indicated that the 2-substituents of the 4-cyanophenyl moiety were more favorable to the improvement in antiviral activity. Thus, we further introduced a trifluoromethyl (**A6**), fluoro (**A7**), chloro (**A8**), or bromo (**A9**) group at the 2-position of the 4-cyanophenyl group. Compound **A6** with 2-CF_3_-4-CN and compound **A7** with 2-F-4-CN were less potent with EC_50_ values of 0.49 and 0.24 μM, respectively. Compound **A8** with 2-Cl-4-CN and compound **A9** with 2-Br-4-CN exhibited potent anti-WT HIV-1 activity with EC_50_ values of 0.063 and 0.079 μM, respectively. The activity of compound **A8** was comparable to that of compound **A5** with the highest potency against WT HIV-1 among compounds **A1**–**A9**, which were 2~3-fold higher than that of NVP (EC_50_ = 0.20 μM). Next, we introduced di-substituents on the 4-cyanophenyl to obtain compounds **A10**–**A12** with EC_50_ values ranging from 0.082 to 0.30 μM. Compared to the corresponding mono-substituted compounds on the 4-cyanophenyl ring (**A8**), 2,6-disubstituted compounds (**A10**) were less active. Compound **A10** with 2,6-diCl-4-CN exhibited anti-HIV-1 activity with an EC_50_ value of 0.082 μM. Compounds **A11**–**A12** with di-substituents on the 4-cyanophenyl ring exhibited low activity. Compound **A12** with 2-Me-4-CN-5-Br exhibited low cytotoxicity with a CC_50_ value of 13.2 μM.

**Figure 3:**
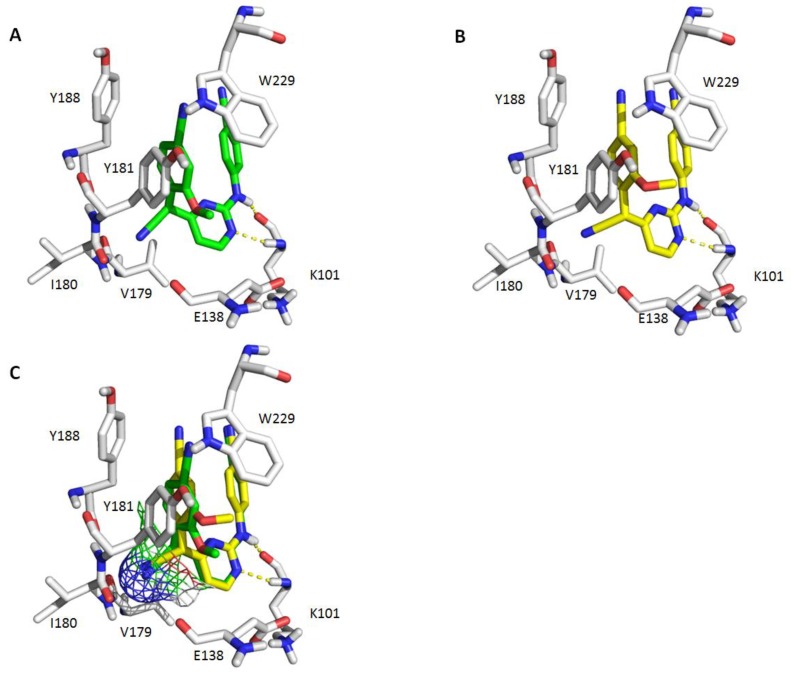
Predicted binding modes of compound **A5** with reverse transcriptase (RT) (PDB: 2ZD1). (**A**) RT with *S***-A5** (green); (**B**) RT with *R***-A5** (yellow); The carbons of the compounds are depicted in green or yellow. Residues involved in interactions are shown as gray sticks. Hydrogen bonds are depicted as yellow dashed lines. (**C**) The cyano linker of *S***-A5** (green) and *R***-A5** (yellow) docking with the small hydrophobic pockets formed by Y181/Y188 and V179.

Molecular docking was carried out to predict the interaction of the best compound **A5** with HIV-1 RT. As shown in Figure 3A,B, both *S*-**A5** and *R*-**A5** employed very similar “U”-like conformation with RT. They formed two key hydrogen bonds between K101 and the pyrimidine N1 (N…HN distance = 2.3 Å) and the *p*-cyanoaniline hydrogen (NH…O=C distance = 1.8 Å). The ring B of **A5** efficiently occupied the hydrophobic pockets. The small hydrophobic pockets in the regions of Y181/Y188 and V179 were occupied by the cyano group and adjacent to the nonpolar group V179 (CN…C distance = 3.3 Å), potentially enhancing the hydrophobic interactions (Figure 3C). Considering their similar binding modes with RT, the racemic forms of the target compounds were selected for biological evaluations.

### 2.3. Antiviral Activity of Compounds ***B1***–***B6*** and Binding Conformation Analysis

Next, we introduced a methyl group to the C5-position in the central pyrimidine ring at the entrance channel in order to improve the activity. The obtained new compounds **B1**–**B6** were evaluated for their anti-HIV activity (Table 2). All of them displayed low nanomolar EC_50_ values against the WT HIV-1 strain and different cytotoxicity with CC_50_ values ranging from 6.6 to 108.6 μM. Most compounds displayed similar cytotoxicity with the reference EFV (CC_50_ = 6.3 μM). Compared with the non-methyl substituted analogues **A1**–**A12**, the methyl group at the C5-position (R^1^) of the pyrimidine core significantly increased the anti-HIV-1 activity by 6~30-fold. The activity of compound **B2** with 2-F-4-CN and compound **B3** with 2-Cl-4-CN had EC_50_ values of 0.04 and 0.01 μM, respectively. Compound **B5** with 2-Me-3-Cl showed an EC_50_ of 0.02 μM. To our delight, **B4** and **B6** exhibited single-digit nanomolar antiviral potency. The activity of **B6** had an EC_50_ of 0.008 μM, which was comparable to the positive NNRTI drugs. Compound **B4** with 2-Br-4-CN displayed the highest potency against HIV-1 with an EC_50_ value of 0.006 μM and a selectivity index (SI) value of 1086, which was superior to the reference drug NVP (EC_50_ = 0.20 μM, SI > 76) and similar to the references EFV and ETR (EC_50_ = 0.003 and 0.005 μM, SI > 3939 and >1012, respectively). These results suggested that a methyl group at the C5-position (R^1^) of the pyrimidine core was favorable for antiviral potency. 

**Figure 4:**
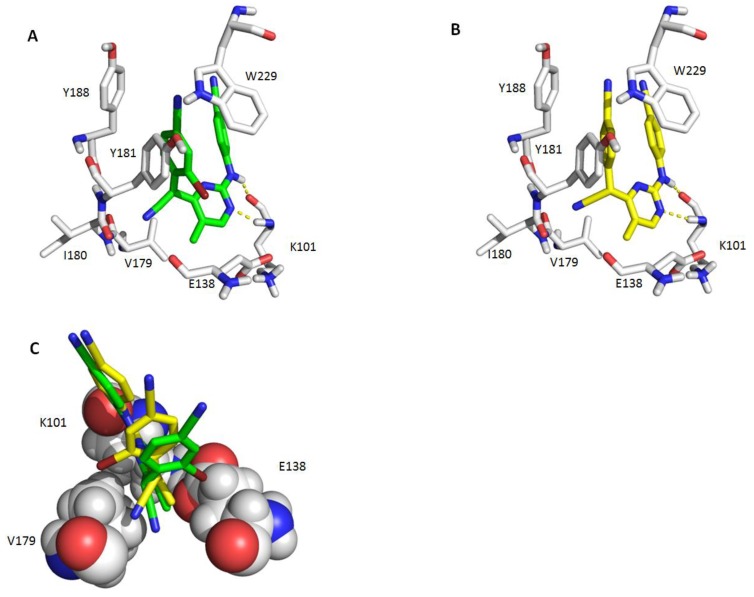
Predicted binding modes of compound **B4** with RT (PDB: 2ZD1). (**A**) RT with *S***-B4** (green); (**B**) RT with *R***-B4** (yellow); (**C**) The space-filling model for the predicted binding mode of *S***-B4** (green) and *R***-B4** (yellow). The carbons of the compounds are depicted in green or yellow. Residues involved in interactions are shown as gray sticks. Hydrogen bonds are depicted as yellow dashed lines.

Molecular docking predicted that the R-configuration of **B4** had a similar binding conformation with the S-configuration (Figure 4A,B). For the C-5 methyl group, nonpolar interactions between the new methyl of compound **B4** and hydrophobic side chains in the entrance channel cleft might be established in addition to the typical interactions. The methyl group was predicted to be located in the hydrophobic region of the protein (Figure 4C), conceivably contributing to the enhanced inhibitory efficacy.

### 2.4. Antiviral Activity against Mutants of Compounds ***A1***–***A12*** and ***B1***–***B6*** and Enzymatic Assay of Compounds ***B1***–***B6***

Furthermore, we tested the antiviral activity against a panel of clinically observed drug-resistant mutants (Table 3). Series A showed very low activity against the WT HIV-1. The compounds **B1**–**B6** with relatively higher anti-HIV-1 activity and SI values were more sensitive toward mutant strains. Among them, compounds **B3**–**B4** and **B6** had better potency against the mutants than other analogues in series A. Toward the K103N mutant, compound **B6** had an EC_50_ of 0.06 μM, which was more potent than the reference standards NVP (EC_50_ =4.70 μM) and EFV (EC_50_ = 0.08 μM). The activities of compounds **B3** (EC_50_ = 0.08 μM) and **B4** (EC_50_ = 0.08 μM) were comparable to those of EFV. Toward the E138K mutant, compounds **B3** (EC_50_ = 0.14 μM) and **B4** (EC_50_ = 0.11 μM) were more potent than NVP (EC_50_ = 0.20 μM). They were inferior to the references EFV and ETR against the L100I and Y181C mutant strains. Furthermore, compounds **B3**–**B6** were superior to NVP (EC_50_ = 4.28 μM) toward the virus with double mutations F227L + V106A. Compound **B4** showed an EC_50_ of 2.35 μM. Compound **B6** exhibited the highest potency against the double mutant F227L + V106A among all target compounds with EC_50_ values of 1.52 μM. However, toward the virus with double mutations K103N + Y181C, all target compounds exhibited low activity.

Next, series B were selected to further test the binding affinity toward the RT enzyme. All the novel DAPY derivatives displayed better inhibitory activity against WT HIV-1 RT than the reference standards NVP (IC_50_ = 0.45 μM) except compound **B1**. Moreover, compound **B3** showed an IC_50_ of 0.05 μM, both compounds **B4** and **B6** had an IC_50_ of 0.06 μM, the activity was higher than NVP (IC_50_ = 0.45 μM) by 7~9-fold.

**Figure 5:**
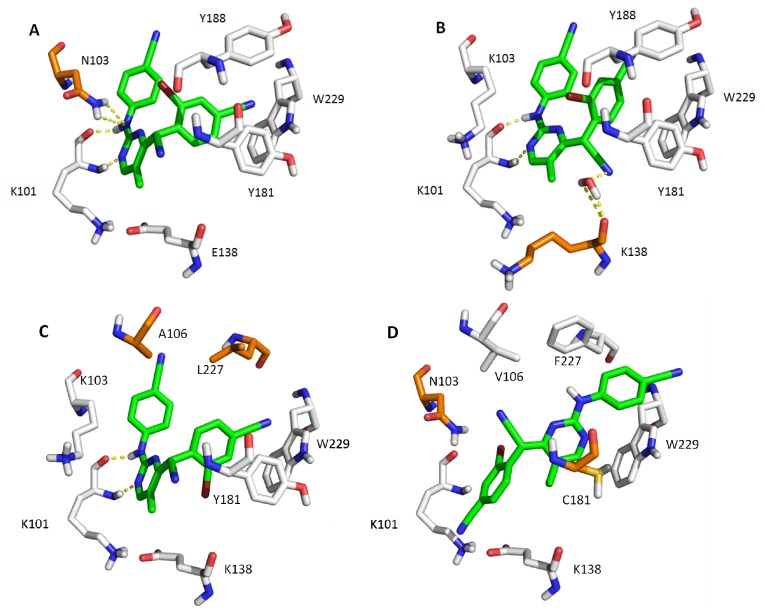
(**A**) Predicted binding modes of compound **B4** with K103N. (**B**) Predicted binding modes of compound **B4** with E138K (PDB: 2ZD1). (**C**) Predicted binding modes of compound **B4** with F227L + V106A. (**D**) Predicted binding modes of compound **B4** with K103N + Y181C. Mutated residues are depicted as orange sticks. The carbons of the compounds are depicted in green. Residues involved in interactions are shown as gray sticks. Hydrogen bonds are depicted as yellow dashed lines.

As shown in Figure 5A–C, **B4** efficiently occupied the pockets of the single amino acid mutations K103N and E138K and the double mutants F227L + V106A with an approximately “U” conformation, which was one of the essential condition for the antiviral activity in the DAPY scaffold. The hydrogen-bonding interactions between the NH linker and K101 was maintained. The N103 residue might be involved in additional hydrogen-bonding interactions with the linkage amide. A water-mediated hydrogen bond between the cyano-methylene linkage and K138 was formed. The F227L decreased the π-π stacking interaction with the p-CN-phenyl ring leading to lower activity compared to those of Etravirine and Rilpivirine. Docking of **B4** with K103N + Y181C predicted that several common features (“U” binding conformation, hydrogen bonds, and π-π stacking interactions) would be lost, which might contribute to the decrease in antiviral activity of compound **B4** against the HIV-1 double mutants K103N + Y181C.

## 3. Materials and Methods

### 3.1. Synthesis

All reagents and solvents were purchased from commercial sources and used as received. Column chromatography was performed on silica gel (300–400 mesh). TLC was carried out on 0.25 mm silica gel plates visualized with UV light (λ = 254 nm) and/or by staining with ethanolic phosphomolybdic acid (PMA) or iodine. Proton nuclear magnetic resonance (^1^H NMR) and carbon nuclear magnetic resonance (^13^C NMR) spectra were recorded in DMSO-*d*_6_, THF-*d*_8_, CDCl_3_, or Acetone-*d*_6_ on a Bruker AV-400 spectrometer with tetramethylsilane (TMS) as the internal standard. Chemical shifts (*δ*) are given in ppm relative to TMS, coupling constants (*J*) in Hz. Melting points were measured on a WRS-1B digital melting point apparatus. Mass spectra and HRMS were obtained on a Waters Quattro Micromass instrument and Brukersolari X-70 FT-MS instrument, respectively, using electrospray ionization (ESI) techniques. The purities of the target compounds were ≥95%, measured by LC-MS, performed on an Waters 2695 system with UV detector and high-performance liquid chromatography (HPLC) (Agilent 1260) using a C18 column (Eslipse XDB, 4.6*150 mm, 5 μm), eluting with a mixture of water (A) and methanol (B) (V_A_:V_B_ = 50:50 to 10:90) over 30 min with a flow rate of 0.8 mL/min and detection at 254 nm.

#### 3.1.1. General Procedure for the Preparation of Compounds **6a-l**

Methyl cyanoacetate (20 mmol, 2 equiv.) was slowly added to a suspension of sodium hydride (22.5 mmol, 60% dispersion in mineral oil, 2.25 equiv.) in DMSO (10 mL) at 0 °C. The mixture was stirred for 1h at room temperature before **5a-l** (10 mmol, 1 equiv.) was added as a solution in DMSO (10 mL) via cannula. The yellow solution was heated to 90 °C for 2.5 h, H_2_O (40 mL) was added, and the reaction was heated to reflux for 8 h. The mixture was cooled to r.t. and stirred for 10 h, the resulting precipitate was filtered, washed with water, and dried to afford **6a-l**.

#### 3.1.2. General Procedure for the Preparation of Compounds **A1**–**A12** and **B1**–**B6**

A solution of **10** (4 mmol, 1 equiv.), appropriate 4-cyanophenylacetonitrile (5.24 mmol, 1.3 equiv.), and anhydrous DMF (10 mL) was stirred for 0.5 h at −15 °C under N_2_. Then, NaH (13.71 mmol; 60% dispersion in mineral oil, 2 equiv.) was added portion-wise at −15 °C. Then, the mixture was stirred at −15 °C for another 2 h, warmed to room temperature, and then reacted for 48–72 h (monitored by TLC). The resulting mixture was poured into a saturated ammonium chloride solution. The precipitate was collected by filtration and the residue was then purified via column chromatography on silica gel, eluting with EtOAc/petroleum ether (1:3) to obtain compounds **A1**–**A12** and **B1**–**B6** as white or yellow solids. Full characterization data for all final compounds can be found in the Supporting Information.

**4-(4-(cyano-(4-cyanophenyl)methyl)-pyrimidin-2-ylamino)benzonitrile (A1)** Yield = 87%. mp: 243.2–244.7 °C. ^1^H NMR (400 MHz, THF-*d*_8_) *δ* 9.39 (s, NH), 8.40 (d, *J* = 5.0 Hz, 1H), 7.80 (d, *J* = 8.7 Hz, 2H), 7.68 (d, *J* = 8.2 Hz, 2H), 7.58 (d, *J* = 8.2 Hz, 2H), 7.48 (d, *J* = 8.7 Hz, 2H), 6.86 (d, *J* = 5.0 Hz, 1H), 5.53 (s, 1H). ^13^C NMR (101 MHz, THF-*d*_8_) *δ* 163.6, 159.6, 159.3, 143.8, 139.0, 132.4 (2C), 132.2 (2C), 128.6 (2C), 120.3, 118.2 (2C), 117.1, 116.8, 112.5, 110.4, 104.3, 43.5. HRMS (ESI): *m/z* calcd for C_20_H_12_N_6_ [M − H]^+^ 335.1051, found 335.1053.

**4-(4-(cyano-(3-methyl-4-cyanophenyl)methyl)-pyrimidin-2-ylamino)benzonitrile (A2)** Yield = 49%. mp: 223.7–224.9 °C. ^1^H NMR (400 MHz, THF-*d*_8_) *δ* 9.38 (s, NH), 8.39 (d, *J* = 4.8 Hz, 1H), 7.82 (d, *J* = 7.1 Hz, 2H), 7.60 (d, *J* = 7.9 Hz, 1H), 7.44–7.49 (m, 3H), 7.37 (d, *J* = 7.6 Hz, 1H), 6.84 (d, *J* = 3.1 Hz, 1H), 5.46 (s, 1H), 2.40 (s, 3H). ^13^C NMR (101 MHz, THF-*d*_8_) *δ* 163.7, 159.6, 159.2, 143.9, 142.4, 138.8, 132.7, 132.2 (2C), 129.4, 125.8, 118.9, 118.2 (2C), 116.8, 116.3, 112.9, 110.4, 104.3, 43.5, 19.0. HRMS (ESI): *m/z* calcd for C_21_H_14_N_6_ [M + Na]^+^ 373.1172, found 373.1173. HPLC analysis: retention time = 11.71 min; peak area, 97.24%.

**4-(4-(cyano-(2-methyl-4-cyanophenyl)methyl)-pyrimidin-2-ylamino)benzonitrile (A3)** Yield = 47%. mp: 213.5–214.6 °C. ^1^H NMR (400 MHz, Acetone-*d*_6_) *δ* 9.43 (s, NH), 8.64 (d, *J* = 5.0 Hz, 1H), 7.99 (d, *J* = 8.3 Hz, 2H), 7.80 (s, 3H), 7.68 (d, *J* = 8.3 Hz, 2H), 7.10 (d, *J* = 4.5 Hz, 1H), 5.99 (s, 1H), 2.52 (s, 3H). ^13^C NMR (101 MHz, Acetone-*d*_6_) *δ* 163.5, 160.1, 159.8, 144.3, 138.6, 137.9, 134.5, 132.8 (2C), 130.5, 130.3, 118.9 (2C), 118.8, 117.9, 117.5, 112.7, 111.5, 104.4, 41.6, 18.6. HRMS (ESI): *m/z* calcd for C_21_H_14_N_6_ [M + Na]^+^ 373.1172, found 373.1170.

**4-(4-(cyano-(3-methoxy-4-cyanophenyl)methyl)-pyrimidin-2-ylamino)benzonitrile (A4)** Yield = 63%. mp:156.2–157.0 °C.^1^H NMR (400 MHz, Acetone-*d*_6_) *δ* 9.44 (s, 1H, NH), 8.60 (d, *J* = 5.0 Hz, 1H), 8.08 (d, *J* = 8.5 Hz, 2H), 7.79 (d, *J* = 8.2 Hz, 1H), 7.70 (d, *J* = 8.5 Hz, 2H), 7.47 (s, 1H), 7.33 (d, *J* = 8.3 Hz, 1H), 7.12 (d, *J* = 5.0 Hz, 1H), 5.81 (s, 1H), 4.00 (s, 3H). ^13^C NMR (101 MHz, Acetone-*d*_6_) *δ* 163.9, 161.6, 160.1, 159.8, 144.4, 141.3, 134.5, 132.9 (2C), 120.8, 118.9 (2C), 118.8, 117.5, 115.3, 112.1, 111.5, 104.4, 101.9, 56.1, 44.1. HRMS (ESI): *m/z* calcd for C_21_H_14_N_6_O [M + Na]^+^ 389.1121, found 389.1121.

**4-(4-(cyano-(2-methoxy-4-cyanophenyl)methyl)-pyrimidin-2-ylamino)benzonitrile (A5)** Yield = 54%. mp: 189.4–190.1 °C. ^1^H NMR (400 MHz, Acetone-*d*_6_) *δ* 9.38 (s, NH), 8.60 (d, *J* = 5.0 Hz, 1H), 8.02 (d, *J* = 8.6 Hz, 2H), 7.77 (d, *J* = 8.2 Hz, 1H), 7.68 (d, *J* = 8.8 Hz, 2H), 7.56 (m, 2H), 7.08 (d, *J* = 5.0 Hz, 1H), 5.93 (s, 1H), 4.00 (s, 3H). ^13^C NMR (101 MHz, DMSO-*d*_6_) *δ* 163.8, 160.2, 159.6, 157.1, 144.9, 133.3 (2C), 131.4, 127.9, 125.6, 119.6, 118.9 (2C), 118.8, 118.3, 115.9, 113.5, 111.8, 103.3, 57.1, 40.6. HRMS (ESI): *m/z* calcd for C_21_H_14_N_6_O [M + Na]^+^ 389.1121, found 389.1123. HPLC analysis: retention time = 8.96 min; peak area, 96.35%.

**4-(4-(cyano-(2-trifluoromethyl-4-cyanophenyl)methyl)-pyrimidin-2-ylamino)benzonitrile (A6)** Yield = 37%. mp: 236.7–237.5 °C. ^1^H NMR (400 MHz, THF-*d*_8_) *δ* 9.43 (s, NH), 8.43 (d, *J* = 4.8 Hz, 1H), 8.21 (s, 1H), 8.01 (m, 1H), 7.87 (m, 1H), 7.72 (d, *J* = 4.5 Hz, 2H), 7.47 (m, 2H), 6.85 (d, *J* = 4.8 Hz, 1H), 5.82 (s, 1H). ^13^C NMR (101 MHz, THF-*d*_8_) *δ* 163.2, 159.9, 159.8, 144.1, 137.2, 136.6, 132.7, 132.6 (2C), 130.7 (t, *J*_C-F_ = 6 Hz), 129.3, 123.2 (t, *J*_C-F_ = 273 Hz), 118.6 (2C), 118.5, 116.7, 116.4, 113.9, 111.3, 104.8, 40.4. HRMS (ESI): *m/z* calcd for C_21_H_11_F_3_N_6_ [M + Na]^+^ 427.0889, found 427.0889. HPLC analysis: retention time = 9.90 min; peak area, 97.54%.

**4-(4-(cyano-(2-fluoro-4-cyanophenyl)methyl)-pyrimidin-2-ylamino)benzonitrile (A7)** Yield = 47%. mp:165.7–166.5 °C. ^1^H NMR (400 MHz, Acetone-*d*_6_) *δ* 9.43 (s, NH), 8.66 (d, *J* = 5.0 Hz, 1H), 7.96 (m, *J* = 17.0, 8.0 Hz, 3H), 7.85 (m, 2H), 7.68 (d, *J* = 8.5 Hz, 2H), 7.17 (d, *J* = 5.0 Hz, 1H), 6.07 (s, 1H). ^13^C NMR (101 MHz, Chloroform-*d*) *δ* 162.7, 160.3, 159.8, 159.7 (d, *J*_C-F_ = 251.7 Hz), 144.2, 132.8 (2C), 131.9 (d, *J*_C-F_ = 3.2 Hz), 129.4 (d, *J*_C-F_ = 3.9 Hz), 127.3 (d, *J*_C-F_ = 14.1 Hz), 120.0 (d, *J*_C-F_ = 25 Hz), 118.9, 118.8 (2C), 116.8, 116.6, 114.5 (d, *J*_C-F_ = 9.9 Hz), 111.3, 104.4, 38.4 (d, *J*_C-F_ = 2.4 Hz). HRMS (ESI): *m/z* calcd for C_20_H_11_FN_6_ [M − H]^+^ 353.0956, found 353.0941.

**4-(4-(cyano-(2-chloro-4-cyanophenyl)methyl)-pyrimidin-2-ylamino)benzonitrile (A8)** Yield = 53%. mp: 219.8–221.6 °C. ^1^H NMR (400 MHz, Acetone-*d*_6_) *δ* 9.40 (s, NH), 8.64 (d, *J* = 4.8 Hz, 1H), 8.08 (s, 1H), 7.98 (d, *J* = 8.5 Hz, 2H), 7.93 (d, *J* = 8.2 Hz, 2H), 7.65 (d, *J* = 7.7 Hz, 2H), 7.16 (d, *J* = 4.8 Hz, 1H), 6.14 (s, 1H). ^13^C NMR (101 MHz, Acetone-*d*_6_) *δ* 162.6, 160.2, 159.8, 144.2, 137.2, 134.4, 133.6, 132.8 (2C), 132.0, 131.7, 119.6, 118.9, 118.8 (2C), 116.7, 114.4, 111.9, 104.4, 41.9. HRMS (ESI): *m/z* calcd for C_20_H_11_ClN_6_ [M + Na]^+^ 393.0626, found 393.0628. HPLC analysis: retention time = 9.94 min; peak area, 98.79%.

**4-(4-(cyano-(2-bromo-4-cyanophenyl)methyl)-pyrimidin-2-ylamino)benzonitrile (A9)** Yield = 59%. mp: 223.5–224.7 °C. ^1^H NMR (400 MHz, THF-*d*_8_) *δ* 9.43 (s, NH), 8.42 (m, 1H), 8.08 (s, 1H), 7.75–7.78 (m, 4H), 7.46 (d, *J* = 8.7 Hz, 2H), 6.90 (d, *J* = 4.9 Hz, 1H), 5.90 (s, 1H). ^13^C NMR (101 MHz, THF-*d*_8_) *δ* 162.4, 159.6, 159.3, 143.8, 138.5, 136.4, 132.2 (2C), 131.5, 131.1, 123.7, 118.1 (2C), 116.1, 115.8, 114.3, 111.0, 110.8, 104.3, 43.6. HRMS (ESI): *m/z* calcd for C_20_H_11_BrN_6_ [M + Na]^+^ 413.0156, found 423.0159. HPLC analysis: retention time = 10.12 min; peak area, 95.35%.

**4-(4-(cyano-(2,6-dichloro-4-cyanophenyl)methyl)-pyrimidin-2-ylamino)benzonitrile (A10)** Yield = 73%. mp: 152.5–153.2 °C. ^1^H NMR (400 MHz, Chloroform-*d*) *δ* 8.55 (d, *J* = 5.0 Hz, 1H), 7.76 (s, 1H), 7.62 (d, *J* = 8.7 Hz, 2H), 7.55 (d, *J* = 8.7 Hz, 2H), 7.39 (s, 1H), 7.06 (d, *J* = 4.9 Hz, 1H), 6.18 (s, 1H). ^13^C NMR (101 MHz, Chloroform-*d*) *δ* 161.5, 159.7, 159.1, 142.7, 137.3 (2C), 134.9, 133.2 (2C), 132.1 (2C), 119.0, 118.5 (2C), 115.5, 115.3, 114.2, 111.0, 105.5, 40.1. HRMS (ESI): *m/z* calcd for C_20_H_10_Cl_2_N_6_ [M + Na]^+^ 427.0236, found 427.0232. HPLC analysis: retention time = 10.29 min; peak area, 98.48%.

**4-(4-(cyano-(2-methyl-3-chloro-4-cyanophenyl)methyl)-pyrimidin-2-ylamino)benzonitrile (A11)** Yield = 81%. mp:196.5–197.7 °C.^1^H NMR (400 MHz, Acetone-*d*_6_) *δ* 9.48 (s, NH), 8.65 (d, *J* = 4.9 Hz, 1H), 7.97 (d, *J* = 8.4 Hz, 3H), 7.81 (d, *J* = 8.1 Hz, 1H), 7.68 (d, *J* = 8.5 Hz, 2H), 7.12 (d, *J* = 4.8 Hz, 1H), 6.13 (s, 1H), 2.55 (s, 3H). ^13^C NMR (101 MHz, Acetone-*d*_6_) *δ* 163.2, 160.2, 159.8, 144.2, 139.6, 137.1, 132.9, 132.2 (2C), 128.9, 118.9, 118.8 (2C), 118.7, 117.3, 115.6, 114.0, 111.6, 104.4, 42.4, 16.4. HRMS (ESI): *m/z* calcd for C_21_H_13_ClN_6_ [M + Na]^+^ 407.0782, found 407.0784. HPLC analysis: retention time = 11.24 min; peak area, 95.39%.

**4-(4-(cyano-(2-methyl-5-bromo-4-cyanophenyl)methyl)-pyrimidin-2-ylamino)benzonitrile (A12)** Yield = 65%. mp: 213.9–214.5 °C.^1^H NMR (400 MHz, Acetone-*d*_6_) *δ* 9.45 (s, NH), 8.65 (d, *J* = 5.0 Hz, 1H), 8.03 (s, 1H), 7.97 (d, *J* = 8.5 Hz, 2H), 7.88 (s, 1H), 7.69 (d, *J* = 8.8 Hz, 2H), 7.16 (d, *J* = 5.0 Hz, 1H), 6.01 (s, 1H), 2.48 (s, 3H). ^13^C NMR (101 MHz, THF-*d*_8_) *δ* 162.7, 159.6, 159.4, 143.7, 139.0, 137.2, 136.3, 133.2, 132.2 (2C), 121.8, 118.2 (2C), 118.1, 116.4, 115.7, 115.6, 110.5, 104.4, 41.0, 17.6. HRMS (ESI): *m/z* calcd for C_21_H_13_BrN_6_ [M-H]^+^ 427.0312, found 427.0302. HPLC analysis: retention time = 11.30 min; peak area, 98.15%.

**4-(4-(cyano-(4-cyanophenyl)methyl)-5-methylpyrimidin-2-ylamino)benzonitrile (B1)** Yield = 71%. mp: 243.1–243.7 °C. ^1^H NMR (400 MHz, Acetone-*d*_6_) *δ* 9.35 (s, NH), 8.46 (s, 1H), 8.06 (d, *J* = 8.5 Hz, 2H), 7.92 (d, *J* = 8.4 Hz, 2H), 7.80 (d, *J* = 8.2 Hz, 2H), 7.68 (d, *J* = 8.7 Hz, 2H), 6.04 (s, 1H), 2.31 (s, 3H). ^13^C NMR (101 MHz, Acetone-*d*_6_) *δ* 161.2, 160.7, 158.4, 144.7, 139.0, 133.1 (2C), 132.9 (2C), 129.5 (2C), 120.4, 119.0, 118.4 (2C), 118.3, 117.5, 112.6, 103.9, 41.5, 13.5. HRMS (ESI): *m/z* calcd for C_21_H_14_N_6_ [M + Na]^+^ 373.1172, found 373.1165.

**4-(4-(cyano-(2-fluoro-4-cyanophenyl)methyl)-5 methylpyrimidin-2-ylamino)benzonitrile (B2)** Yield = 47%. mp:211.9–212.9 °C. ^1^H NMR (400 MHz, Acetone-*d*_6_) *δ* 9.32 (s, NH), 8.51 (s, 1H), 7.83–7.93 (m, 5H), 7.64 (d, *J* = 8.5 Hz, 2H), 6.20 (s, 1H), 2.37 (s, 3H). ^13^C NMR (101 MHz, DMSO-*d*_6_) *δ* 161.3, 159.9(d, *J*_C-F_ = 250.0 Hz), 159.9, 145.0, 133.3 (2C), 132.0 (d, *J*_C-F_ = 4.0 Hz), 130.1 (d, *J*_C-F_ = 3.0 Hz), 127.0 (d, *J*_C-F_ = 14.4 Hz), 120.7, 120.6, 120.5, 119.9, 118.5 (2C), 117,7 (d, *J*_C-F_ = 3.0 Hz), 117.3, 113.9 (d, *J*_C-F_ = 11 Hz), 103.0, 36.5 (d, *J*_C-F_ = 1.0 Hz), 14.2. HRMS (ESI): *m/z* calcd for C_21_H_13_FN_6_ [M + Na]^+^ 391.1078, found 391.1070. HPLC analysis: retention time = 9.78 min; peak area, 95.05%.

**4-(4-(cyano-(2-chloro-4-cyanophenyl)methyl)-5-methylpyrimidin-2-ylamino)benzonitrile (B3)** Yield = 62%. mp: 213.6–214.2 °C. ^1^H NMR (400 MHz, DMSO-*d*_6_) *δ* 10.26 (s, NH), 8.53 (s, 1H), 8.26 (s, 1H), 8.07 (d, *J* = 7.8 Hz, 1H), 7.78 (d, *J* = 8.0 Hz, 1H), 7.68 (d, *J* = 8.4 Hz, 2H), 7.59 (d, *J* = 8.6 Hz, 2H), 6.37 (s, 1H), 2.24 (s, 3H). ^13^C NMR (101 MHz, DMSO-*d*_6_) *δ* 161.3, 160.2, 158.2, 145.0, 137.3, 134.1, 134.0, 133.2 (2C), 132.5, 131.8, 121.0, 119.9, 118.5 (2C), 117.5, 117.2, 113.8, 102.9, 40.8, 14.3. HRMS (ESI): *m/z* calcd for C_21_H_13_ClN_6_ [M + Na]^+^ 407.0782, found 407.0778. HPLC analysis: retention time = 10.33 min; peak area, 96.14%.

**4-(4-(cyano-(2-bromo-4-cyanophenyl)methyl)-5-methylpyrimidin-2-ylamino)benzonitrile (B4)** Yield = 72%. mp: 238.9–240.1 °C. ^1^H NMR (400 MHz, Acetone-*d*_6_) *δ* 9.27 (s, NH), 8.49 (s, 1H), 8.22 (d, *J* = 1.3 Hz, 1H), 8.05–8.08 (m, *J* = 8.1, 1.4 Hz, 1H), 7.91 (d, *J* = 8.1 Hz, 1H), 7.79 (d, *J* = 8.5 Hz, 2H), 7.57 (d, *J* = 8.7 Hz, 2H), 6.14 (s, 1H), 2.35 (s, 3H). ^13^C NMR (101 MHz, DMSO-*d*_6_) *δ* 161.2, 160.4, 158.2, 145.0, 139.1, 137.1, 133.2 (2C), 132.9, 131.7, 124.4, 121.1, 119.9, 118.5 (2C), 117.4, 117.3, 113.9, 102.9, 42.4, 14.4. HRMS (ESI): *m/z* calcd for C_21_H_13_BrN_6_ [M + Na]^+^ 451.0277, found 451.0276. HPLC analysis: retention time = 10.75 min; peak area, 96.09%.

**4-(4-(cyano-(2-methyl-3-chloro-4-cyanophenyl)methyl)-5-methylpyrimidin-2-ylamino)benzonitrile (B5)** Yield = 65%. mp: 205.4–206.5 °C. ^1^H NMR (400 MHz, Acetone-*d*_6_) *δ* 9.32 (s, NH), 8.50 (s, 1H), 7.96 (d, *J* = 8.1 Hz, 1H), 7.87 (d, *J* = 8.5 Hz, 2H), 7.69 (d, *J* = 8.1 Hz, 1H), 7.61 (d, *J* = 8.7 Hz, 2H), 6.20 (s, 1H), 2.51 (s, 3H), 2.31 (s, 3H). ^13^C NMR (101 MHz, DMSO-*d*_6_) *δ* 161.3, 160.4, 158.3, 145.1, 139.6, 137.1, 136.9, 133.2 (2C), 132.9, 128.8, 120.8, 119.9, 118.5 (2C), 117.7, 116.5, 113.4, 102.9, 40.5, 16.9, 14.3. HRMS (ESI): *m/z* calcd for C_22_H_15_ClN_6_ [M + Na]^+^ 421.0939, found 421.0937. HPLC analysis: retention time = 11.15 min; peak area, 95.15%.

**4-(4-(cyano-(2-methyl-5-bromo-4-cyanophenyl)methyl)-5-methylpyrimidin-2-ylamino)benzonitrile (B6)** Yield = 76%. mp: 232.3–233.9 °C. ^1^H NMR (400 MHz, THF-*d*_8_) *δ* 9.43 (s, NH), 8.42 (s, 1H), 7.79–7.85 (m, *J* = 11 Hz, 4H), 7.57 (d, *J* = 8.7 Hz, 2H), 5.94 (s, 1H), 2.35 (s, 3H), 2.25 (s, 3H). ^13^C NMR (101 MHz, Acetone-*d*_6_) *δ* 160.9, 160.0, 158.4, 144.5, 139.3, 137.8, 136.7, 133.1, 132.8 (2C), 122.0, 120.8, 119.0, 118.3 (2C), 118.2, 116.5, 116.4, 115.6, 103.9, 39.1, 18.0, 13.5. HRMS (ESI): *m/z* calcd for C_22_H_15_BrN_6_ [M + Na]^+^ 465.0434, found 465.0431.

### 3.2. In Vitro Anti-HIV Assay

Evaluation of the antiviral activity of the compounds against HIV in MT-4 cells was performed using the MTT assay as previously described [24,25]. All used NNRTI-resistant strains were a gift of Professor Jan Balzarini from Rega Institute for Medical Research, KU Leuven. All used strains were analyzed and reported earlier [26,27,28]. Prior to use in our assays, every freshly grown virus stock was the subject of a genotypic analysis. In addition to the genotypic analysis, every stock was subject to a phenotypic analysis using a panel of relevant reference compounds in MT-4 cells. In the case of NNRTI-resistant strains the reference compounds used were NVP, EFV, and ETR. Stock solutions (10× final concentration) of test compounds were added in 25 μL volumes to two series of triplicate wells so as to allow simultaneous evaluation of their effects on mock- and HIV-infected cells at the beginning of each experiment. Serial 5-fold dilutions of test compounds were made directly in flat-bottomed 96-well microtiter trays using a Biomek 3000 robot (Beckman instruments, Fullerton, CA). Untreated HIV- and mock-infected cell samples were included as controls. HIV stock (50 μL) at 100–300 CCID_50_ (50% cell culture infectious doses) and culture medium (final 200 μL volume per well) were added to either the infected or mock-infected wells of the microtiter tray. Mock-infected cells were used to evaluate the effects of test compounds on uninfected cells in order to assess the cytotoxicity of the test compounds. Exponentially growing MT-4 cells were centrifuged for 5 min at 220× *g* and the supernatant was discarded. The MT-4 cells were resuspended at 6 × 10^5^ cells/mL and 50 μL volumes were transferred to the microtiter tray wells. Five days after infection, the viability of mock- and HIV-infected cells was examined spectrophotometrically using the MTT assay. The MTT assay is based on the reduction of yellow colored 3-(4,5-dimethylthiazol-2-yl)-2,5-diphenyltetrazolium bromide (MTT) (Acros Organics, Geel, Belgium) by mitochondrial dehydrogenase activity in metabolically active cells to a blue-purple formazan that can be measured spectrophotometrically. The absorbances were read in an eight-channel computer-controlled photometer (Infinite M1000, Tecan, Männedorf, Switzerland) at two wavelengths (540 and 690 nm). All data were calculated using the median absorbance value of three wells. The 50% cytotoxic concentration (CC_50_) was defined as the concentration of the test compound that reduced the absorbance (OD540) of the mock-infected control sample by 50%. The concentration achieving 50% protection against the cytopathic effect of the virus in infected cells was defined as the 50% effective concentration (EC_50_).

### 3.3. Reverse Transcriptase Assay

Recombinant wild type p66/p51 HIV-1 RT was expressed and purified as described [29]. The RT assay was performed with the EnzCheck Reverse Transcriptase Assay kit (Molecular Probes, Invitrogen), as described by the Manufacturer. The assay is based on the dsDNA quantitation reagent PicoGreen. This reagent shows a pronounced increase in fluorescence signal upon binding to dsDNA or RNA–DNA heteroduplexes. Single-stranded nucleic acids generate only minor fluorescence signal enhancement when a sufficiently high dye/base pair ratio is applied [30]. This condition is met in the assay. A poly(rA) template of approximately 350 bases long and an oligo (dT)16 primer are annealed in a molar ratio of 1:1.2 (60 min at room temperature). Then, 52 ng of the RNA/DNA is brought into each well of a 96-well plate in a volume of 20 μL polymerization buffer (60 mM Tris-HCl, 60 mM KCl, 8 mM MgCl_2_, 13 mM DTT, 100 μM dTTP, pH 8.1). Then, 5 μL of RT enzyme solution, diluted to a suitable concentration in enzyme dilution buffer (50 mM Tris-HCl, 20% glycerol, 2 mM DTT, pH 7.6), is added. The reactions are incubated at 25 °C for 40 min and then stopped by the addition of EDTA (15 mM fc). Heteroduplexes are then detected by addition of PicoGreen. Signals are read using an excitation wavelength of 490 nm and emission detection at 523 nm using a spectrofluorometer (Safire 2, Tecan). To test the activity of compounds against RT, 1 μL of compound in DMSO was added to each well before the addition of RT enzyme solution. Control wells without compound contained the same amount of DMSO. Results are expressed as relative fluorescence, i.e., the fluorescence signal of the reaction mix with compound divided by the signal of the same reaction mix without compound.

### 3.4. Molecular Modeling

Molecular modelling research work was performed with the Tripos molecular modelling software package (Sybyl-X 2.0). All the molecules for docking analysis were built using the standard bond lengths and angles from Sybyl-X 2.0/base Builder before being optimized using the Tripos force field for 10,000 generations two times or more, until the minimized conformers of the ligand were the same. The flexible docking method, called Surflex-Dock, docks the ligand automatically into the ligand binding site of the receptor by using a protocol-based approach and an empirically-derived scoring function. The protocol is a computational representation of a putative ligand that binds to the intended binding site and is a unique and essential element of the docking algorithm. The scoring function in Surflex-Dock, which contains hydrophobic, polar, repulsive, entropic, and solvation terms, was trained to estimate the dissociation constant (K_d_) expressed in −log (K_d_)^2^. The scoring function in Surflex-Dock, which contains hydrophobic, polar, repulsive, entropic, and solvation terms, was trained to estimate the binding energy. Prior to docking, the protein was prepared by removing water molecules, the ligand ETR, and other unnecessary small molecules from the crystal structure of the HIV-1 RT complex. Simultaneously, hydrogen atoms were added to the protein. Surflex-Dock default settings were used for other parameters, such as the number of starting conformations per molecule (set to 0), the size to expand the search grid (set to 8 Å), the maximum number of the rotatable bonds per molecule (set to 100), and the maximum number of poses per ligand (set to 20). During the docking procedure, all of the single bonds in amino acid residue side-chains inside the defined RT binding pocket were regarded as rotatable or flexible, and the ligand was allowed to rotate at all single bonds and move flexibly within the tentative binding pocket. The atomic charges were recalculated using the Kollman all-atom approach for the protein and the Gasteiger–Hückel approach for the ligand. The binding interaction energy was calculated, including van der WBls, electrostatic, and torsional energy terms defined in the Tripos force field. The structure optimization was performed for more than 10,000 generations using a genetic algorithm, and the 20 best-scoring ligand–protein complexes were kept for further analyses. The −log(K_d_)^2^ values of the 20 best-scoring complexes, which represented the binding affinities of the ligand with RT, encompassed a wide scope of the functional classes (10^−2^–10^−9^). Only the highest scoring 3D structural model of the ligand-bound RT was chosen to define the binding interaction. 

## 4. Conclusions

**Figure 6:**
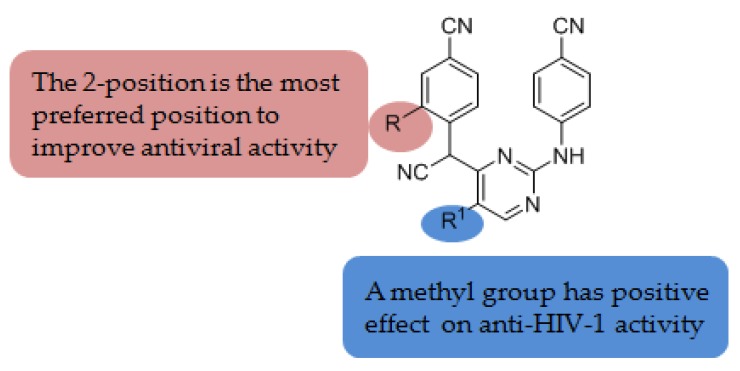
A systematic summary of the structure–activity relationship (SAR).

In summary, we have designed and synthesized a new series of substituted CH(CN)-DAPYs by using a scaffold hopping strategy. All the target compounds displayed anti-HIV-1 activity with micromolar to nanomolar EC_50_ values in HIV-1 infected MT-4 cells. Among the synthesized analogs, compounds **B4** (EC_50_ = 8 nM) and **B6** (EC_50_ = 6 nM) were nearly as potent as EFV and ETR against WT HIV-1. (1) The 2-position of 4-cyanophenyl was the most preferred position to improve antiviral activity. (2) A methyl group at the C5-position (R^1^) of the pyrimidine core could increase the anti-HIV-1 activity (Figure 6). This information might be helpful to further DAPY optimizations.

## Figures and Tables

**Table 1:**
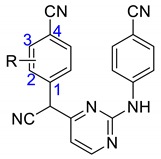
Activity and cytotoxicity against HIV-1 (III_B_) strains in MT-4 cells of compounds **A1**–**A12**.

Compounds	R	EC_50_ (μM) ^2^	CC_50_ (μM) ^3^	SI ^4^
		HIV-1 III_B_^1^		
**A1**	H	3.27 ± 2.25	32.7 ± 9.9	10
**A2**	3-Me	1.17 ± 0.34	16.1 ± 3.6	14
**A3**	2-Me	0.069 ± 0.008	10.9 ± 3.6	157
**A4**	3-OMe	11.74 ± 12.13	78.4 ± 10.4	7
**A5**	2-OMe	0.059 ± 0.011	25.8 ± 8.7	434
**A6**	2-CF_3_	0.49 ± 0.30	6.2 ± 1.4	13
**A7**	2-F	0.24 ± 0.16	10.3 ± 4.5	43
**A8**	2-Cl	0.063 ± 0.009	4.6 ± 2.7	72
**A9**	2-Br	0.079 ± 0.02	3.4 ± 0.3	42
**A10**	2,6-diCl	0.082 ± 0.02	4.5 ± 1.3	55
**A11**	2-Me-3-Cl	0.30 ± 0.03	6.1 ± 0.5	20
**A12**	2-Me-5-Br	0.24 ± 0.04	13.2 ± 1.6	54
Nevirapine		0.20 ± 0.10	>15.0	>76
Etravirine		0.005 ± 0.001	>4.6	>1012
Efavirenz		0.003 ± 0.001	>6.3	>3939
Rilpivirine		0.0016 ± 0.0003	5.9 ± 0.3	3747

^1^ III_B_ means the wild type (WT) of HIV-1 virus strain. ^2^ EC_50_: The effective concentration required to protect MT-4 cells against HIV-induced cytopathogenicity by 50%. ^3^ CC_50_: The cytotoxic concentration of the compound that reduced the normal uninfected MT-4 cell viability by 50%. ^4^ SI: selectivity index, ratio CC_50_/EC_50_ (WT).

**Table 2:**
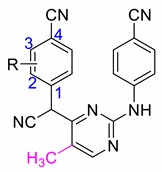
Activity and cytotoxicity against HIV-1 (III_B_) strains in MT-4 cells of compounds **B1**–**B6**.

Compounds	R	EC_50_ (μM) ^2^	CC_50_ (μM) ^3^	SI ^4^
		HIV-1 III_B_^1^		
**B1**	H	0.30 ± 0.11	108.6 ± 80.0	356
**B2**	2-F	0.04 ± 0.02	20.6 ± 3.6	586
**B3**	2-Cl	0.01 ± 0.01	9.6 ± 2.7	920
**B4**	2-Br	0.006 ± 0.001	6.6 ± 1.3	1086
**B5**	2-Me-3-Cl	0.02 ± 0.008	8.6 ± 2.2	370
**B6**	2-Me-5-Br	0.008 ± 0.004	9.8 ± 3.5	1215
Nevirapine		0.20 ± 0.10	>15.0	>76
Etravirine		0.005 ± 0.001	>4.6	>1012
Efavirenz		0.003 ± 0.001	>6.3	>3939
Rilpivirine		0.0016 ± 0.0003	5.9 ± 0.3	3747

^1^ III_B_ means the wild type (WT) of HIV-1 virus strain. ^2^ EC_50_: The effective concentration required to protect MT-4 cells against HIV-induced cytopathogenicity by 50%. ^3^ CC_50_: The cytotoxic concentration of the compound that reduced the normal uninfected MT-4 cell viability by 50%. ^4^ SI: selectivity index, ratio CC_50_/EC_50_ (WT).

**Table 3:** Inhibitory activity of compounds **A1**–**A12** and **B1**–**B6** toward a panel of clinically relevant HIV-1 mutant strains and inhibitory activity of compounds **B1**–**B6** toward WT HIV-1 RT.

Compounds	EC_50_(μM) ^1^						IC_50_ (μM) ^2^
	L100I	K103N	Y181C	E138K	F227L + V106A	K103N + Y181C	
**A1**	≥12.31	>32.70	11.26 ± 1.51	>32.70	>32.70	>32.70	ND ^3^
**A2**	>16.07	>16.07	>16.07	>16.07	>16.07	>16.07	ND
**A3**	5.03 ± 0.55	1.96 ± 0.45	1.71 ± 0.13	3.04 ± 0.39	>10.93	>10.93	ND
**A4**	≥61.36	≥62.18	≥48.12	≥56.55	≥61.00	≥65.72	ND
**A5**	4.72 ± 0.79	0.68 ± 0.16	1.25 ± 0.58	1.63 ± 0.32	≥12.42	>25.85	ND
**A6**	>6.23	>6.23	2.29 ± 0.02	>6.23	>6.23	>6.23	ND
**A7**	>10.36	>10.36	>10.36	>10.36	>10.36	>10.36	ND
**A8**	≥2.37	2.13 ± 0.22	1.87 ± 0.21	2.20 ± 0.35	>4.59	>4.59	ND
**A9**	>3.35	≥1.88	1.88 ± 0.42	2.31 ± 0.02	>3.35	>3.35	ND
**A10**	2.32 ± 0.48	1.78 ± 0.12	2.20 ± 0.27	2.19 ± 0.22	>4.49	>4.49	ND
**A11**	2.32 ± 0.48	>6.11	>6.11	>6.11	>6.11	>6.11	ND
**A12**	>13.23	2.52 ± 0.42	2.78 ± 0.36	3.73 ± 0.59	>13.23	>13.23	ND
**B1**	8.03 ± 4.43	3.95 ± 0.22	7.05 ± 0.96	4.92 ± 0.3	≥58.97	>108.57	1.46 ± 0.09
**B2**	3.04 ± 1.13	1.07 ± 0.37	1.59 ± 0.18	0.97 ± 0.14	>20.63	>20.63	0.22 ± 0.05
**B3**	0.82 ± 0.15	0.08 ± 0.02	0.26 ± 0.02	0.14 ± 0.03	4.04 ± 0.70	>9.56	0.05 ± 0.03
**B4**	0.51 ± 0.19	0.08 ± 0.03	0.16 ± 0.17	0.11 ± 0.04	2.35 ± 0.18	>6.62	0.06 ± 0.03
**B5**	1.82 ± 0.4	0.39 ± 0.07	1.02 ± 0.2	0.60 ± 0.10	1.66 ± 0.27	>8.58	0.07 ± 0.02
**B6**	0.86 ± 0.19	0.06 ± 0.01	0.33 ± 0.10	0.17 ± 0.06	1.52 ± 0.15	>9.79	0.06 ± 0.02
Nevirapine	0.98 ± 0.39	4.70 ± 0.63	6.07 ± 1.55	0.20 ± 0.15	≥4.28	>15.02	0.45 ± 0.25
Etravirine	0.008 ± 0.002	0.003 ± 0.001	0.017 ± 0.003	0.008 ± 0.004	0.014 ± 0.005	0.04 ± 0.01	0.01 ± 0.009
Efavirenz	0.04 ± 0.008	0.08 ± 0.01	0.006 ± 0.001	0.005 ± 0.002	0.28 ± 0.07	0.24 ± 0.06	ND
Rilpivirine	0.002 ± 0.001	0.001 ± 0.0002	0.003 ± 0.001	0.003 ± 0.001	0.04 ± 0.01	0.009 ± 0.002	ND

^1^ EC_50_: The effective concentration required to protect MT-4 cells against HIV-induced cytopathogenicity by 50%. ^2^ IC_50_: inhibitory concentration of the test compound required to inhibit biotin deoxyuridine triphosphate (biotin-dUTP) incorporation into WT HIV-1 RT by 50%. ^3^ ND: not determined.

## Supplementary Materials

The following are available online, containing HRMS, ^1^H-NMR, and ^13^C-NMR spectrum of final compounds.
