# Supplementary material for: Scaffold Hopping in Discovery of HIV-1 Non-Nucleoside Reverse Transcriptase Inhibitors: From CH(CN)-DABOs to CH(CN)-DAPYs
Source: Molecules. 2020 Mar 30;25(7):1581. doi: 10.3390/molecules25071581 (PMC7180830; doi:10.3390/molecules25071581)
Supplement: Supplementary file 1 [file molecules-25-01581-s001.pdf]

Supplementary Materials

Scaffold Hopping in Discovery of HIV-1 Non-nucleoside Reverse Transcriptase Inhibitors: From CH(CN)-DABOs to CH(CN)-DAPYs

Ting-Ting Li [1], Christophe Pannecouque [4], Erik De Clercq [4], Chun-Lin Zhuang [2,3,*] and Fen-Er Chen [1,2,3,*]

[1] *Institute of Pharmaceutical Science and Technology, Zhejiang University of Technology, Hangzhou 310014, People's Republic of China;*

[2] *Department of Chemistry, Fudan University, Shanghai 200433, People's Republic of China;*

[3] *Shanghai Engineering Center of Industrial Asymmetric Catalysis for Chiral Drugs, Shanghai 200433, People's Republic of China*

[4] *Rega Institute for Medical Research, KU Leuven, Herestraat 49, B-3000 Leuven, Belgium*

*Author to whom correspondence should be addressed.

E-mail: rfchen@fudan.edu.cn (F.-E. C); zclnathan@163.com (C.-L. Z);

HRMS, HPLC and NMR spectra of final compounds

1. HRMS, ^{1}H-NMR and ^{13}C NMR spectrum of A1

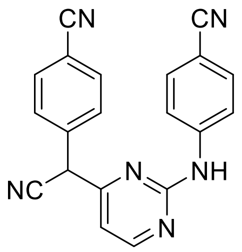

Chemical Formula: $C_{20}H_{12}N_6$
Exact Mass: 336.1123

A1

Display Report

Analysis Info

Analysis Name	D:\Data\data\2019\20200107LTTA-A1_RB2_01_3132.d	Acquisition Date	1/8/2020 10:37:48 AM
Method	MS-2MIN-NEG.m	Operator	BDAL@DE
Sample Name	20200107LTTA-A1	Instrument	compact 8255754.20127
Comment			

Acquisition Parameter

Source Type	ESI	Ion Polarity	Negative	Set Nebulizer	2.0 Bar
Focus	Active	Set Capillary	2800 V	Set Dry Heater	200 °C
Scan Begin	50 m/z	Set End Plate Offset	-500 V	Set Dry Gas	8.0 l/min
Scan End	1500 m/z	Set Charging Voltage	2000 V	Set Divert Valve	Waste
		Set Corona	0 nA	Set APCI Heater	0 °C

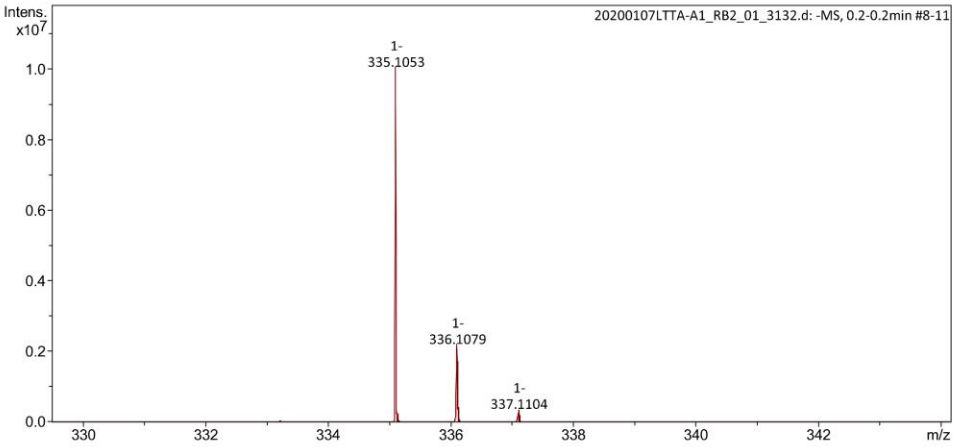

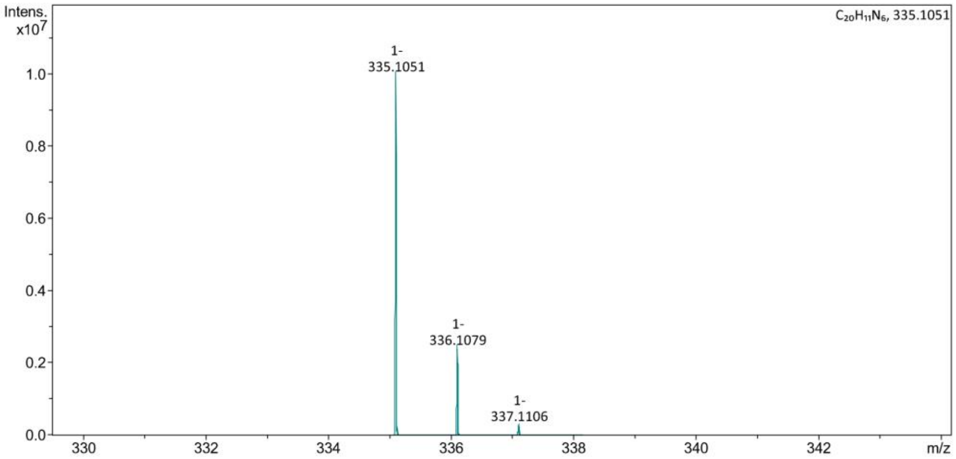

20200107LTTA-A1_RB2_01_3132.d

Bruker Compass DataAnalysis 4.3 printed: 1/8/2020 10:44:17 AM by: BDAL@DE Page 1 of 1

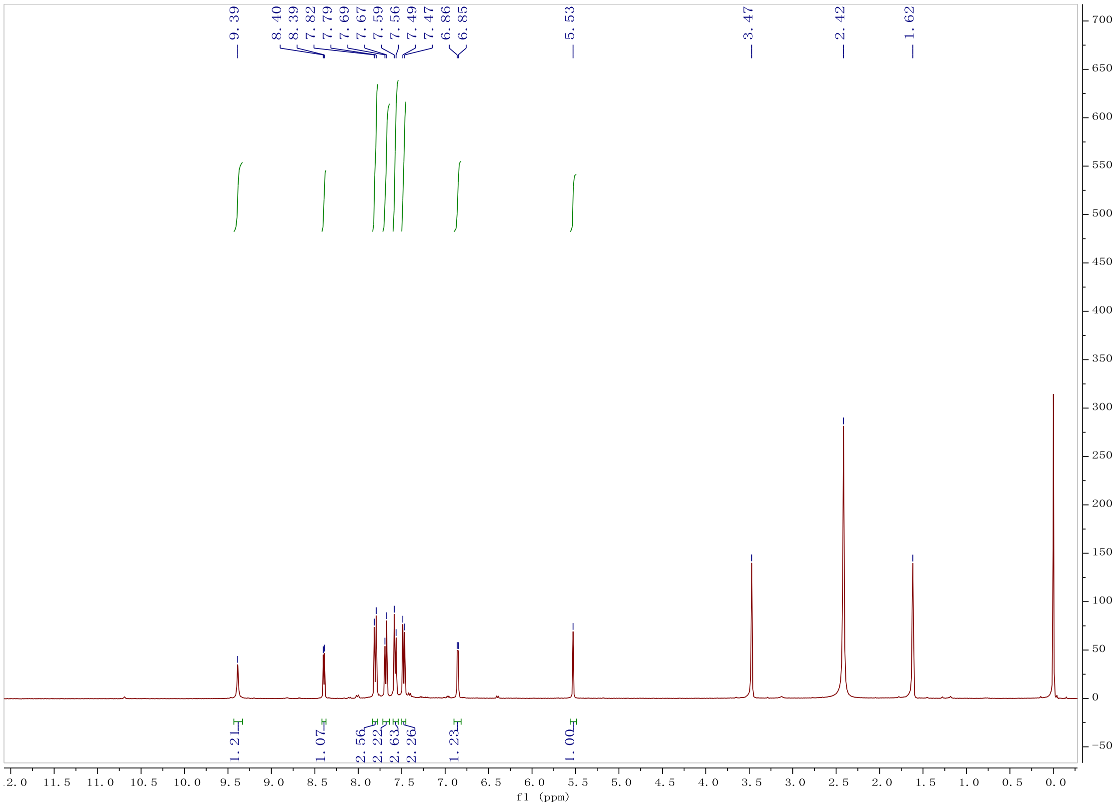
9.39
8.40
8.39
7.82
7.79
7.69
7.67
7.59
7.56
7.49
7.47
6.86
6.85
5.53
3.47
2.42
1.62
1.21
1.07
2.56
2.22
2.63
2.26
1.23
1.00
f1 (ppm)

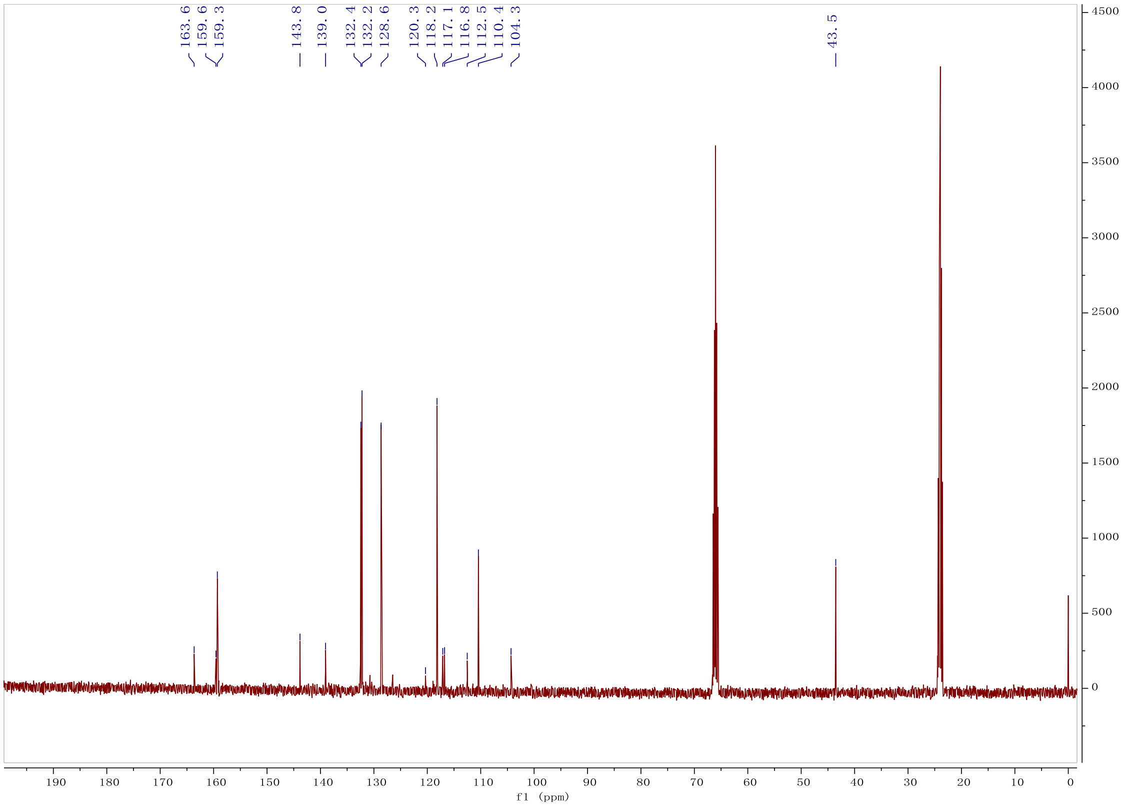
163.6
159.6
159.3
143.8
139.0
132.4
132.2
128.6
120.3
118.2
117.1
116.8
112.5
110.4
104.3
43.5
f1 (ppm)

2. HRMS, HPLC, ^{1}H-NMR and ^{13}C NMR spectrum of A2

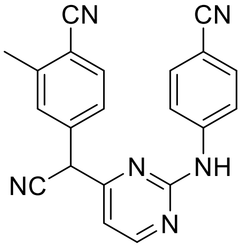

Chemical Formula: $C_{21}H_{14}N_6$
Exact Mass: 350.1280
A2

Display Report

Analysis Info

Analysis Name	D:\Data\data\2019\20200107LTT-B-A2_RA8_01_3131.d	Acquisition Date	1/8/2020 9:47:50 AM
Method	MS-2MIN-POS.m	Operator	BDAL@DE
Sample Name	20200107LTT-B-A2	Instrument	compact 8255754.20127
Comment			

Acquisition Parameter

Source Type	ESI	Ion Polarity	Positive	Set Nebulizer	2.0 Bar
Focus	Active	Set Capillary	4500 V	Set Dry Heater	200 °C
Scan Begin	50 m/z	Set End Plate Offset	-500 V	Set Dry Gas	8.0 l/min
Scan End	3000 m/z	Set Charging Voltage	2000 V	Set Divert Valve	Waste
		Set Corona	0 nA	Set APCI Heater	0 °C

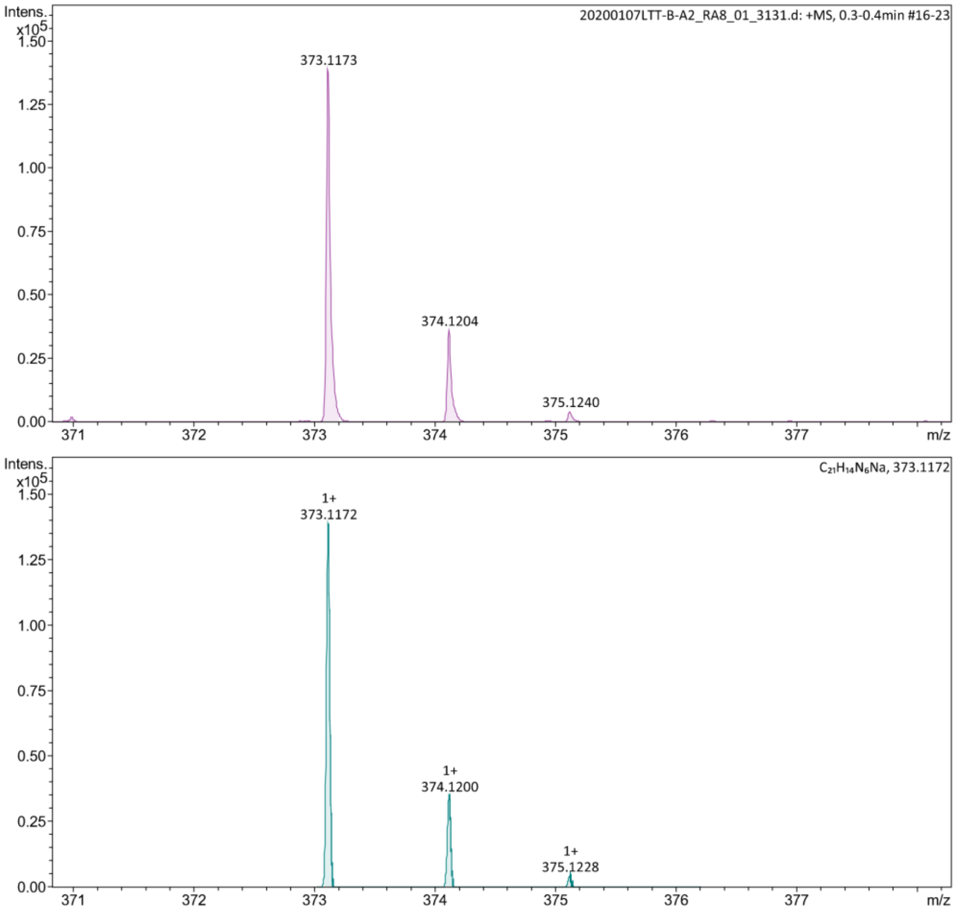

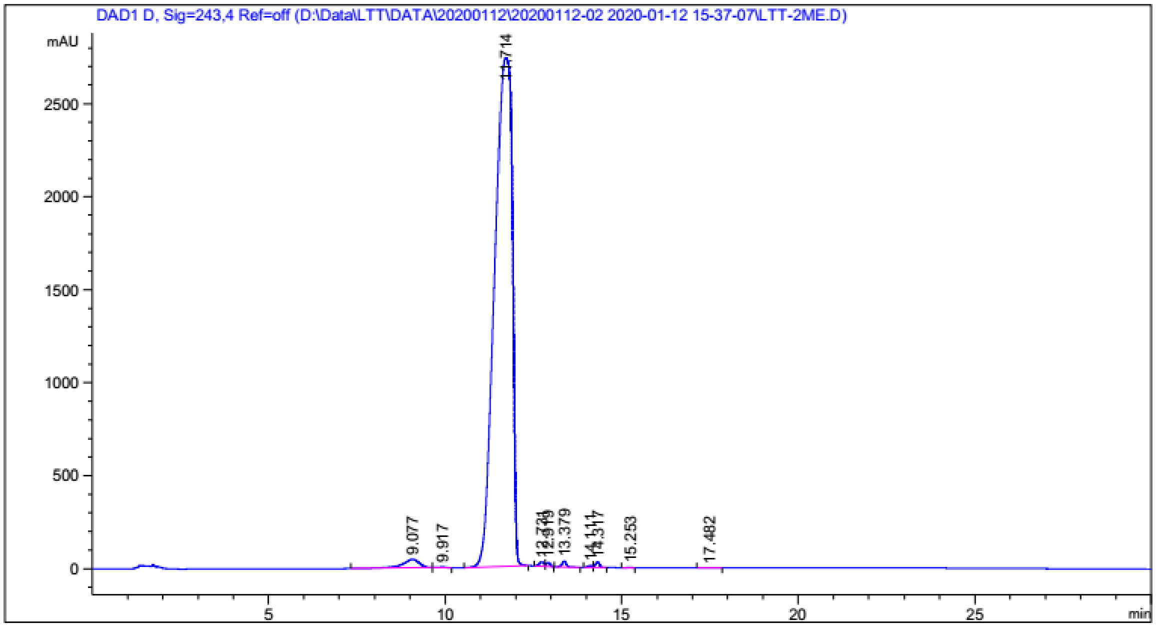

Peak #	RetTime [min]	Type	Width [min]	Area [mAU*s]	Height [mAU]	Area %
1	9.077	BB	0.4096	1550.65747	46.19415	1.6096
2	9.917	BB	0.1795	54.17162	3.54992	0.0562
3	11.714	BB	0.4014	9.36786e4	2733.26343	97.2399
4	12.731	BV	0.1455	229.94943	24.04994	0.2387
5	12.919	VB	0.1212	171.20580	22.13570	0.1777
6	13.379	BB	0.1330	283.75052	31.83833	0.2945
7	14.111	BV	0.1193	88.58525	10.45837	0.0920
8	14.317	VB	0.1216	246.14848	29.86804	0.2555
9	15.253	BB	0.1221	15.80414	1.55519	0.0164
10	17.482	BB	0.1726	18.78978	1.29221	0.0195

Totals : 9.63377e4 2904.20529

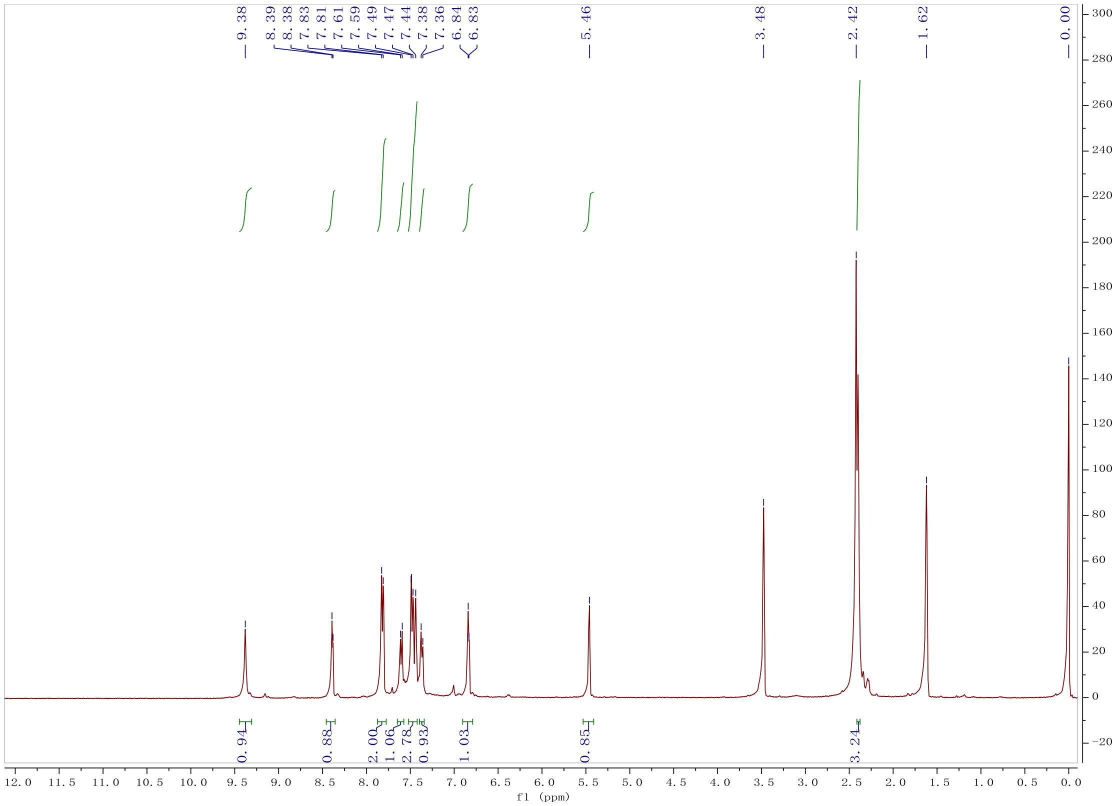
9.38
8.39
8.38
7.83
7.81
7.61
7.59
7.49
7.47
7.44
7.38
7.36
6.84
6.83
5.46
3.48
2.42
1.62
0.00
0.94
0.88
2.00
1.06
2.78
0.93
1.03
0.85
3.24
f1 (ppm)

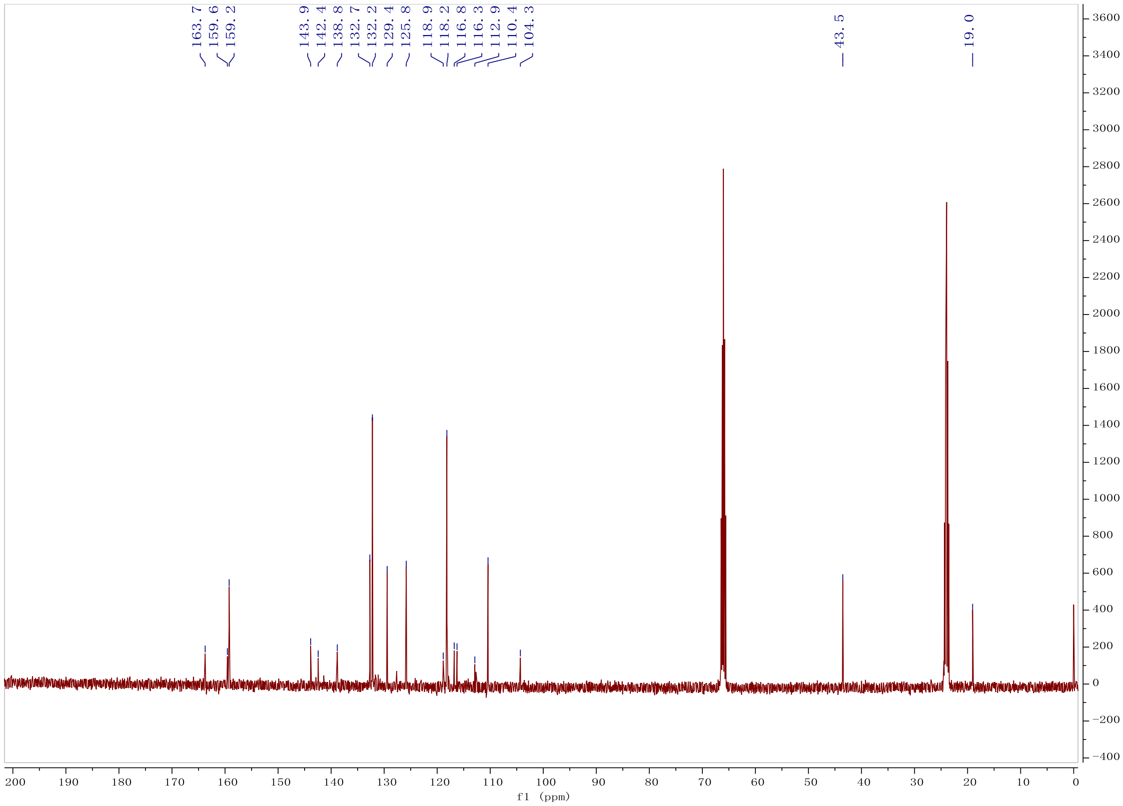
163.7
159.6
159.2
143.9
142.4
138.8
132.7
132.2
129.4
125.8
118.9
118.2
116.8
116.3
112.9
110.4
104.3
43.5
19.0
f1 (ppm)

3. HRMS, ^{1}H-NMR and ^{13}C NMR spectrum of A3

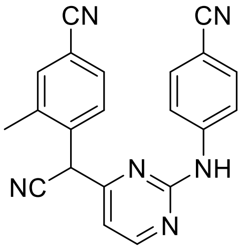

Chemical Formula: $C_{21}H_{14}N_6$
Exact Mass: 350.1280
A3

Display Report

Analysis Info

Analysis Name	D:\Data\data\2019\20200107LTT-C-A3_RA7_01_3130.d	Acquisition Date	1/8/2020 9:45:04 AM
Method	MS-2MIN-POS.m	Operator	BDAL@DE
Sample Name	20200107LTT-C-A3	Instrument	compact 8255754.20127
Comment			

Acquisition Parameter

Source Type	ESI	Ion Polarity	Positive	Set Nebulizer	2.0 Bar
Focus	Active	Set Capillary	4500 V	Set Dry Heater	200 °C
Scan Begin	50 m/z	Set End Plate Offset	-500 V	Set Dry Gas	8.0 l/min
Scan End	3000 m/z	Set Charging Voltage	2000 V	Set Divert Valve	Waste
		Set Corona	0 nA	Set APCI Heater	0 °C

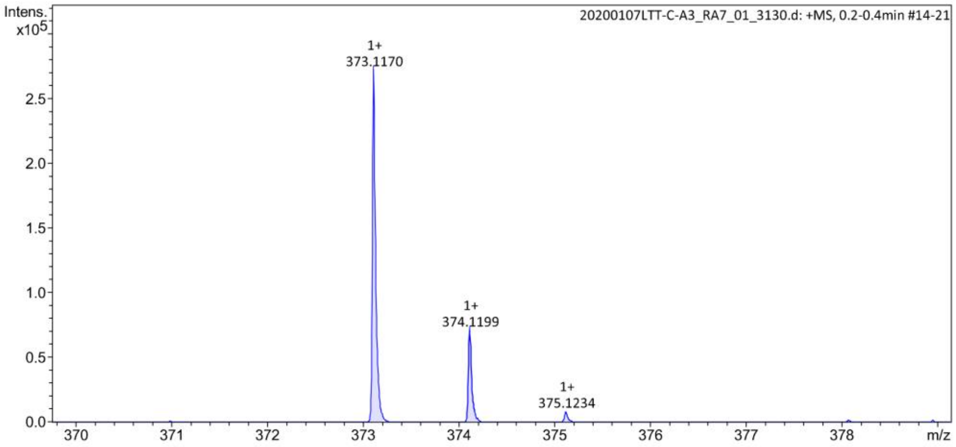

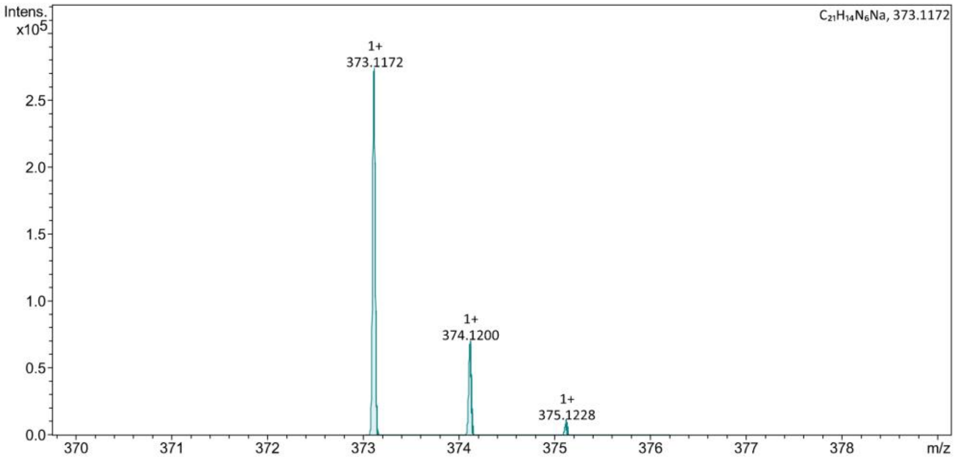

20200107LTT-C-A3_RA7_01_3130.d

Bruker Compass DataAnalysis 4.3 printed: 1/8/2020 10:19:05 AM by: BDAL@DE Page 1 of 1

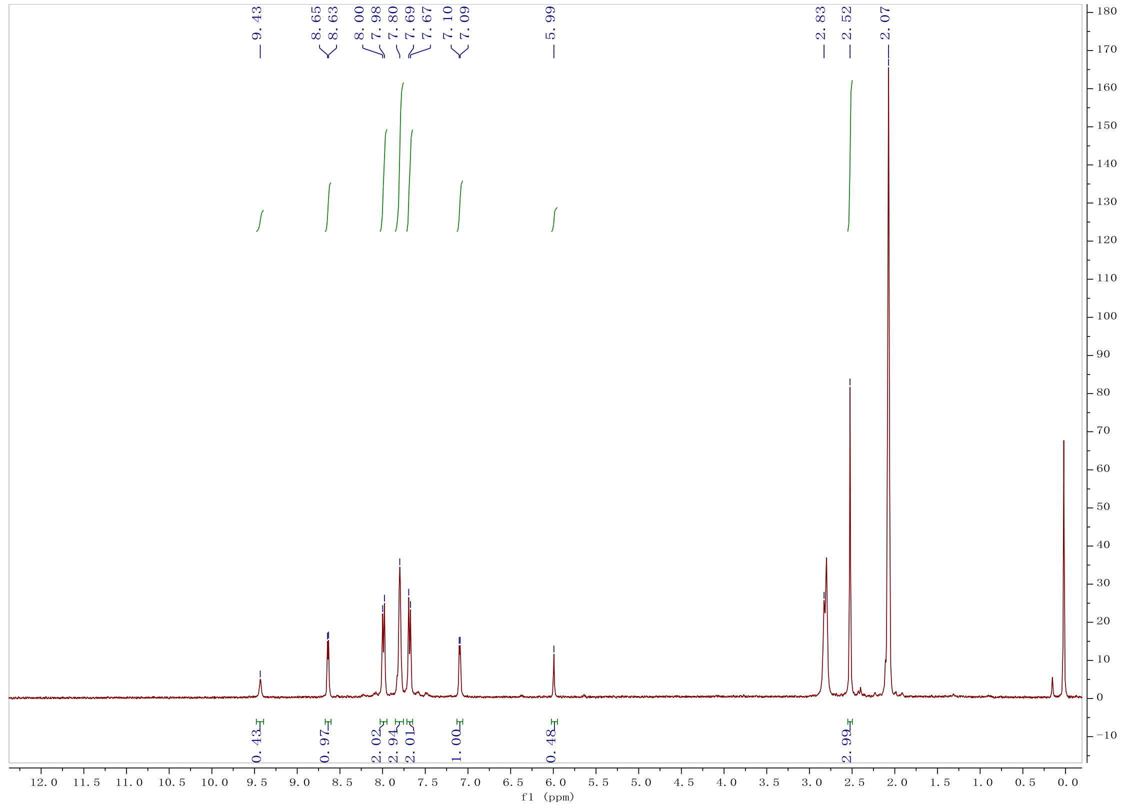

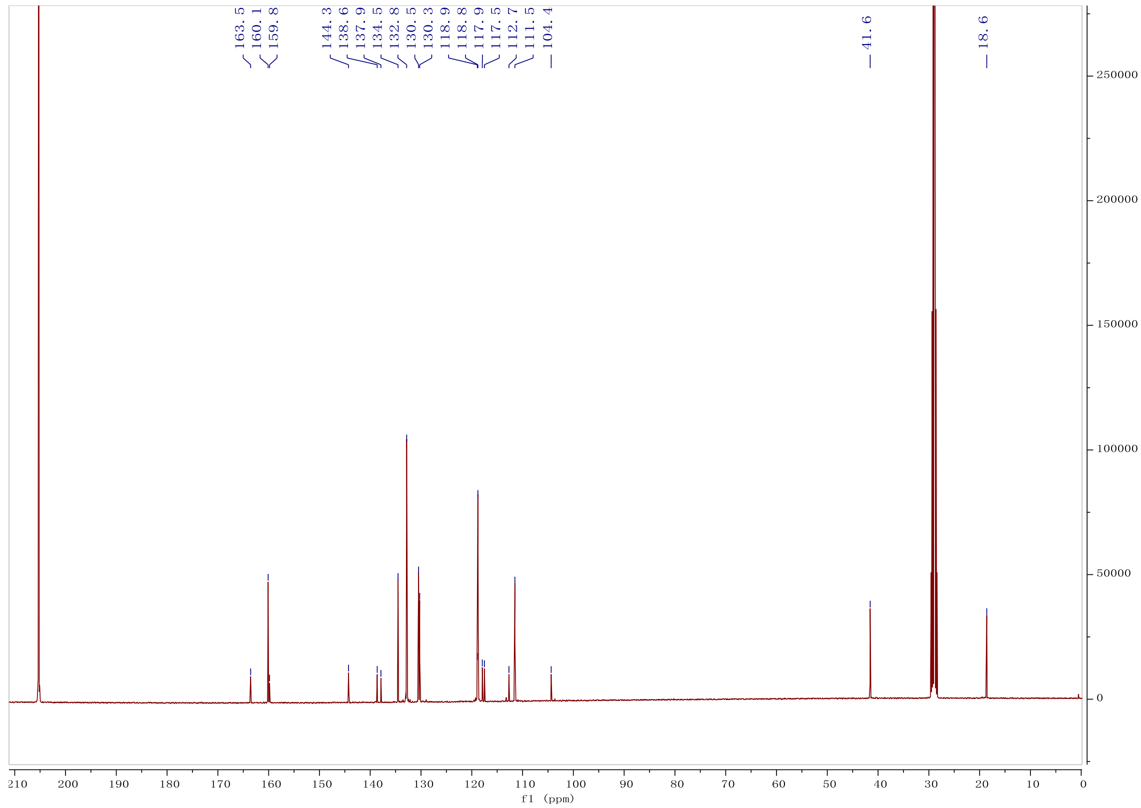

4. HRMS, ^{1}H-NMR and ^{13}C NMR spectrum of A4

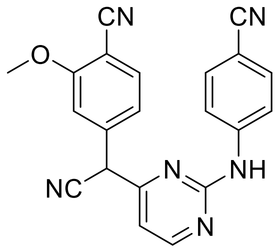

Chemical Formula: $C_{21}H_{14}N_6O$
Exact Mass: 366.1229
A4

Display Report

Analysis Info

Analysis Name	D:\Data\data\2019\20200107LTT-D-A4_RA1_01_3124.d	Acquisition Date	1/8/2020 9:28:30 AM
Method	MS-2MIN-POS.m	Operator	BDAL@DE
Sample Name	20200107LTT-D-A4	Instrument	compact 8255754.20127
Comment			

Acquisition Parameter

Source Type	ESI	Ion Polarity	Positive	Set Nebulizer	2.0 Bar
Focus	Active	Set Capillary	4500 V	Set Dry Heater	200 °C
Scan Begin	50 m/z	Set End Plate Offset	-500 V	Set Dry Gas	8.0 l/min
Scan End	3000 m/z	Set Charging Voltage	2000 V	Set Divert Valve	Waste
		Set Corona	0 nA	Set APCI Heater	0 °C

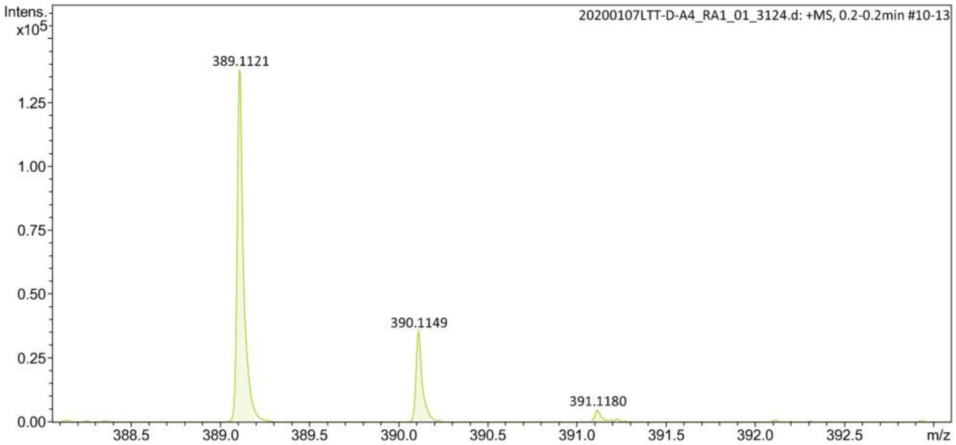

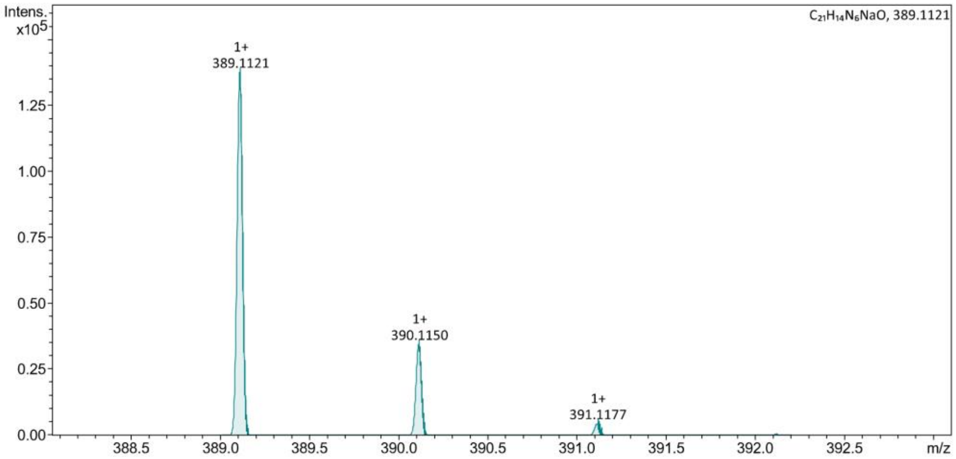

20200107LTT-D-A4_RA1_01_3124.d

Bruker Compass DataAnalysis 4.3 printed: 1/8/2020 10:24:16 AM by: BDAL@DE Page 1 of 1

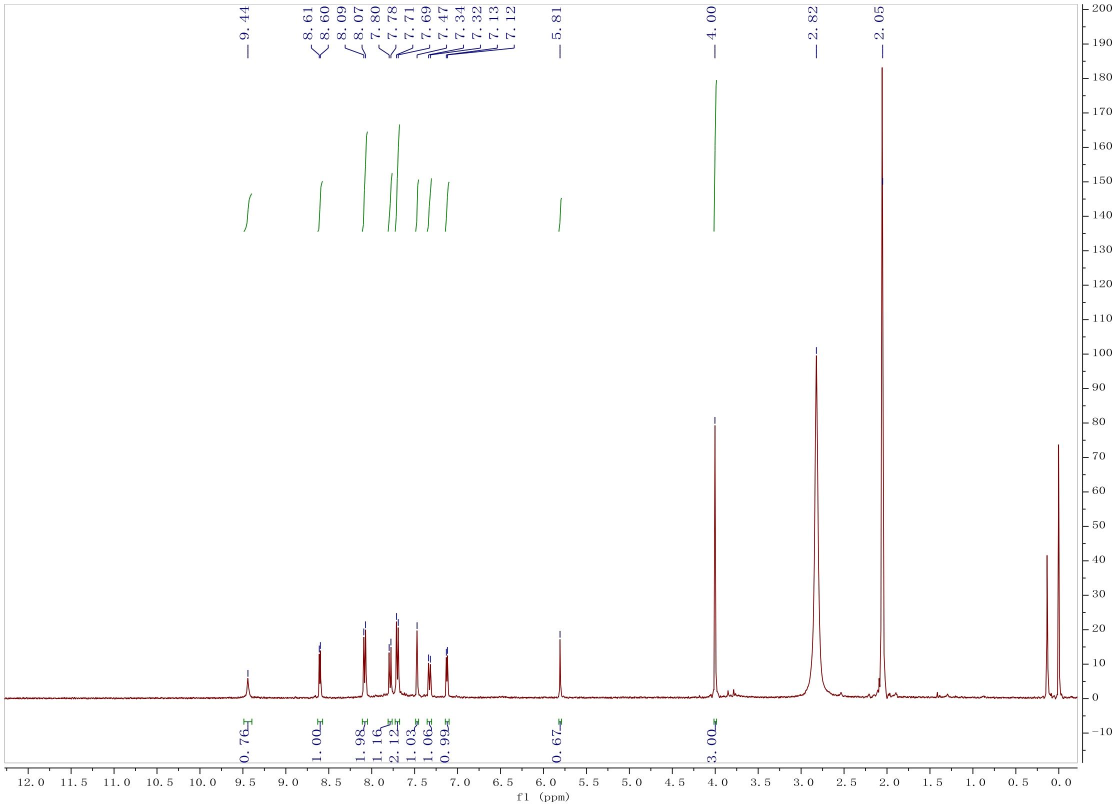
9.44
8.61
8.60
8.09
8.07
7.80
7.78
7.71
7.69
7.47
7.34
7.32
7.13
7.12
5.81
4.00
2.82
2.05
0.76
1.00
1.98
1.16
2.12
1.03
1.06
0.99
0.67
3.00
f1 (ppm)

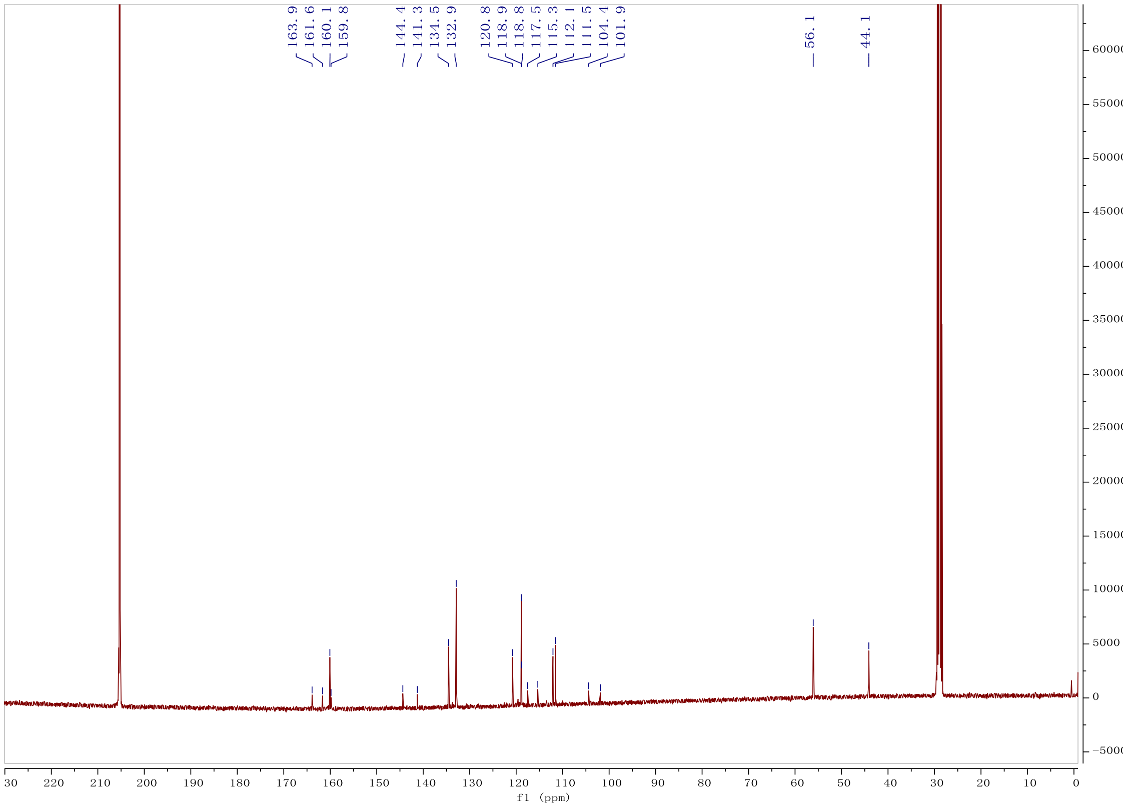
163.9
161.6
160.1
159.8
144.4
141.3
134.5
132.9
120.8
118.9
118.8
117.5
115.3
112.1
111.5
104.4
101.9
56.1
44.1
f1 (ppm)

5. HRMS, HPLC, ^{1}H-NMR and ^{13}C NMR spectrum of A5

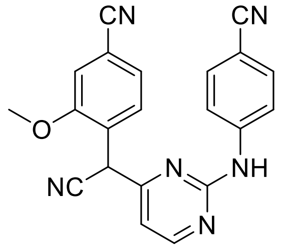

Chemical Formula: $C_{21}H_{14}N_6O$
Exact Mass: 366.1229

A5

Display Report

Analysis Info

Analysis Name	D:\Data\data\2019\20200107LTT-E-A5_RA4_01_3127.d	Acquisition Date	1/8/2020 9:36:46 AM
Method	MS-2MIN-POS.m	Operator	BDAL@DE
Sample Name	20200107LTT-E-A5	Instrument	compact 8255754.20127
Comment			

Acquisition Parameter

Source Type	ESI	Ion Polarity	Positive	Set Nebulizer	2.0 Bar
Focus	Active	Set Capillary	4500 V	Set Dry Heater	200 °C
Scan Begin	50 m/z	Set End Plate Offset	-500 V	Set Dry Gas	8.0 l/min
Scan End	3000 m/z	Set Charging Voltage	2000 V	Set Divert Valve	Waste
		Set Corona	0 nA	Set APCI Heater	0 °C

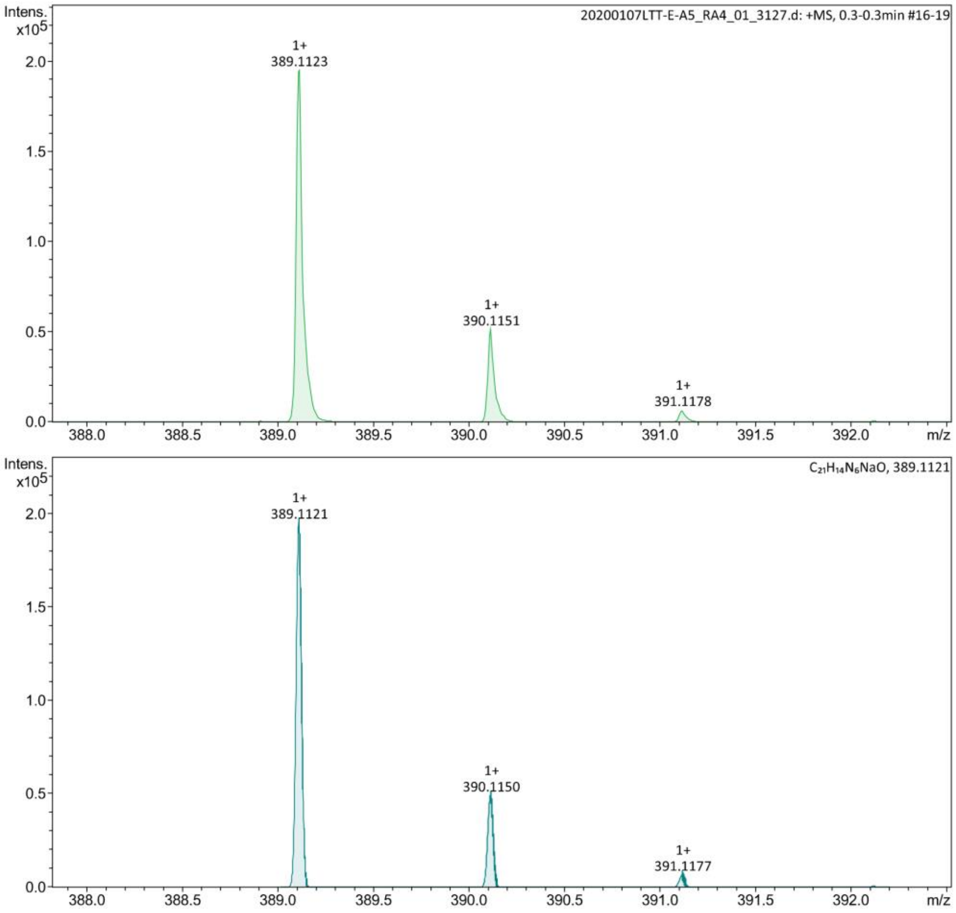

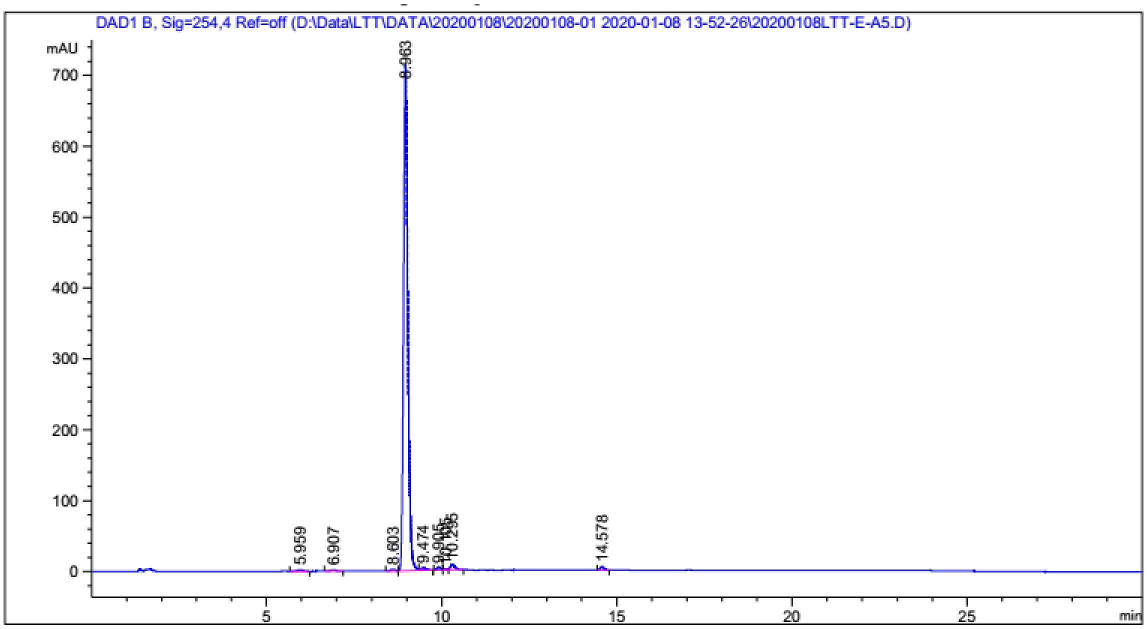

1	5.959	BB	0.1438	21.16767	1.74776	0.3402
2	6.907	BB	0.1329	20.65470	1.84024	0.3319
3	8.603	BB	0.0969	16.28233	2.19895	0.2616
4	8.963	BV R	0.1266	5995.59473	713.36243	96.3452
5	9.474	VB E	0.1191	28.17799	3.02669	0.4528
6	9.905	BV	0.1057	28.27242	3.74086	0.4543
7	10.105	VV E	0.0743	6.82353	1.14825	0.1096
8	10.295	VB R	0.1264	70.43349	7.98897	1.1318
9	14.578	BB	0.0999	35.62985	4.92322	0.5725

Totals : 6223.03670 739.97737

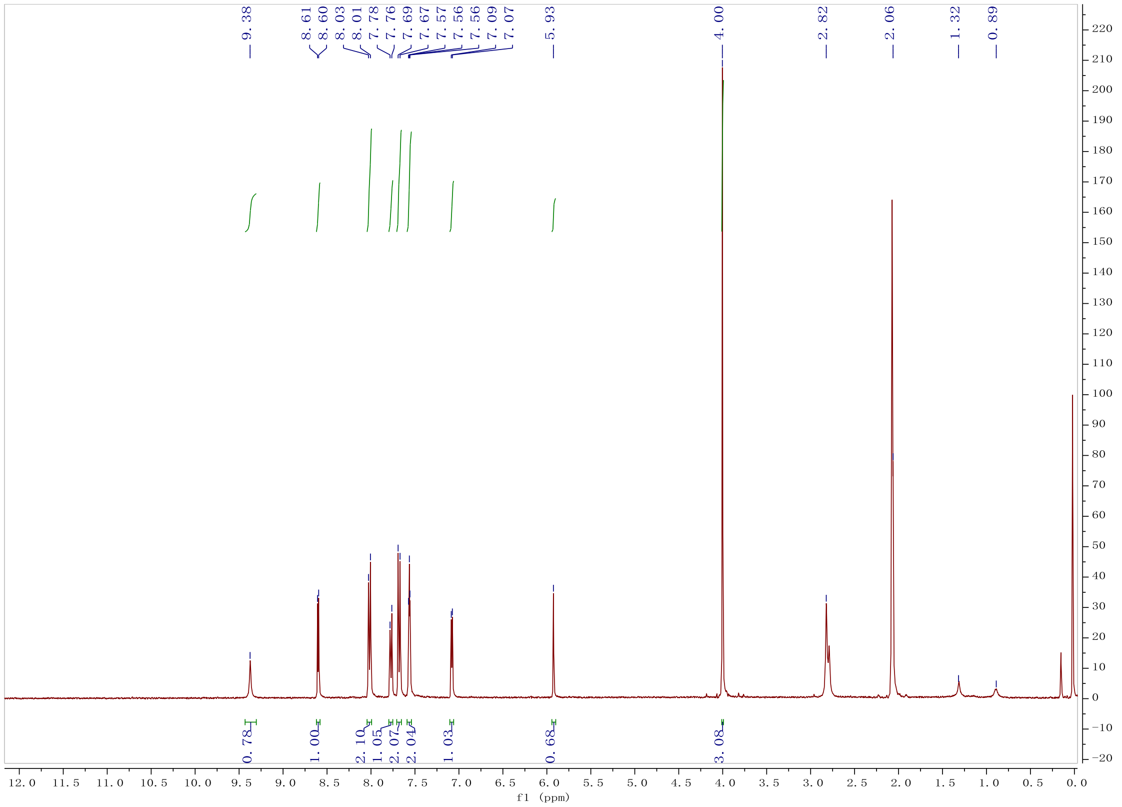
9.38
8.61
8.60
8.03
8.01
7.78
7.76
7.69
7.67
7.57
7.56
7.56
7.09
7.07
5.93
4.00
2.82
2.06
1.32
0.89
0.78
1.00
2.10
1.05
2.07
2.04
1.03
0.68
3.08
f1 (ppm)

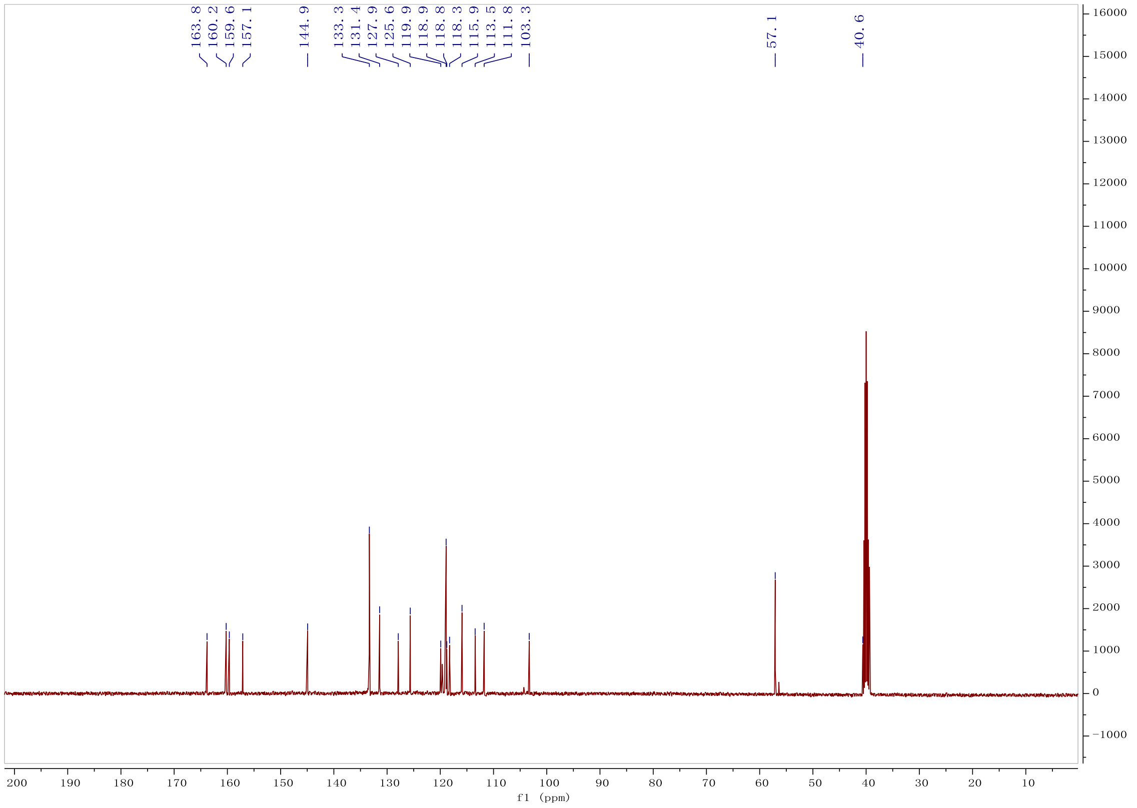
163.8
160.2
159.6
157.1
144.9
133.3
131.4
127.9
125.6
119.9
118.9
118.8
118.3
115.9
113.5
111.8
103.3
57.1
40.6
f1 (ppm)

6. HRMS, HPLC, ^{1}H-NMR and ^{13}C NMR spectrum of A6

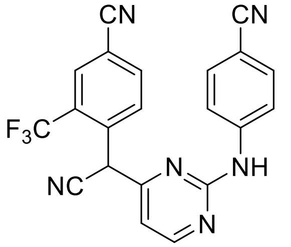

Chemical Formula: $C_{21}H_{11}F_3N_6$

Exact Mass: 404.0997

A6

Display Report

Analysis Info

Analysis Name	D:\Data\data\2019\20200107LTT-F-A6_RB1_01_3123.d	Acquisition Date	1/8/2020 9:25:44 AM
Method	MS-2MIN-POS.m	Operator	BDAL@DE
Sample Name	20200107LTT-F-A6	Instrument	compact 8255754.20127
Comment			

Acquisition Parameter

Source Type	ESI	Ion Polarity	Positive	Set Nebulizer	2.0 Bar
Focus	Active	Set Capillary	4500 V	Set Dry Heater	200 °C
Scan Begin	50 m/z	Set End Plate Offset	-500 V	Set Dry Gas	8.0 l/min
Scan End	3000 m/z	Set Charging Voltage	2000 V	Set Divert Valve	Waste
		Set Corona	0 nA	Set APCI Heater	0 °C

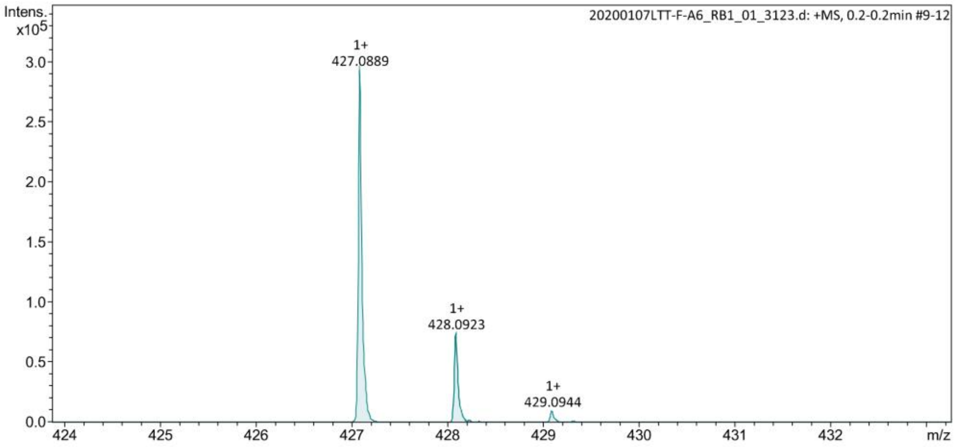

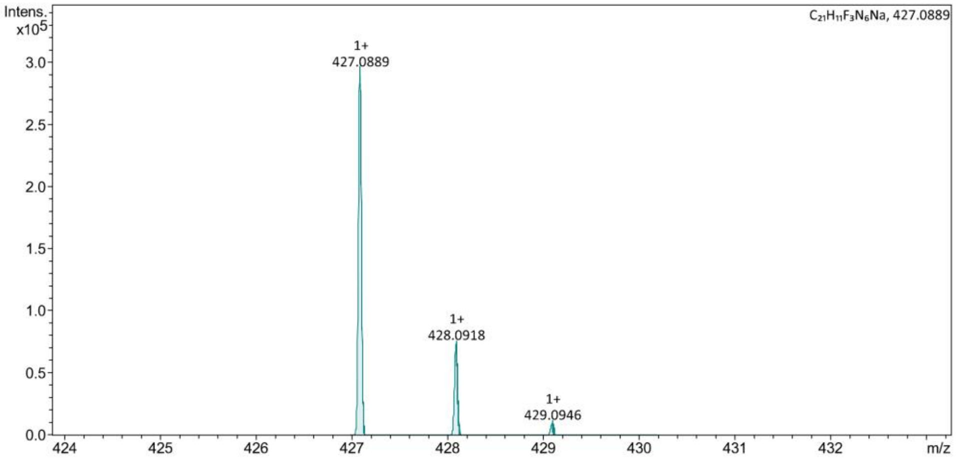

20200107LTT-F-A6_RB1_01_3123.d

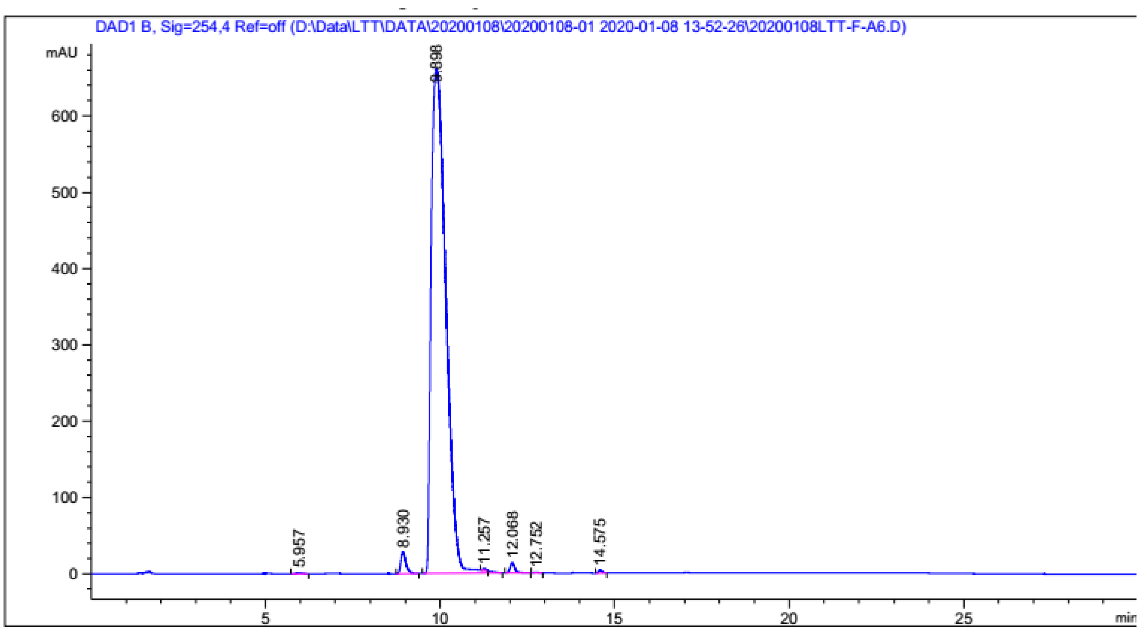

Peak #	RetTime [min]	Type	Width [min]	Area [mAU*s]	Height [mAU]	Area %
1	5.957	BB	0.1270	15.89840	1.47793	0.0810
2	8.930	BB	0.1483	283.85385	28.47197	1.4464
3	9.898	BV R	0.4594	1.91407e4	660.95819	97.5351
4	11.257	VB E	0.0889	17.77038	2.76846	0.0906
5	12.068	BB	0.1487	129.33585	13.27079	0.6591
6	12.752	BB	0.1131	10.01035	1.04692	0.0510
7	14.575	BB	0.1029	26.85141	3.75549	0.1368

Totals : 1.96245e4 711.74975

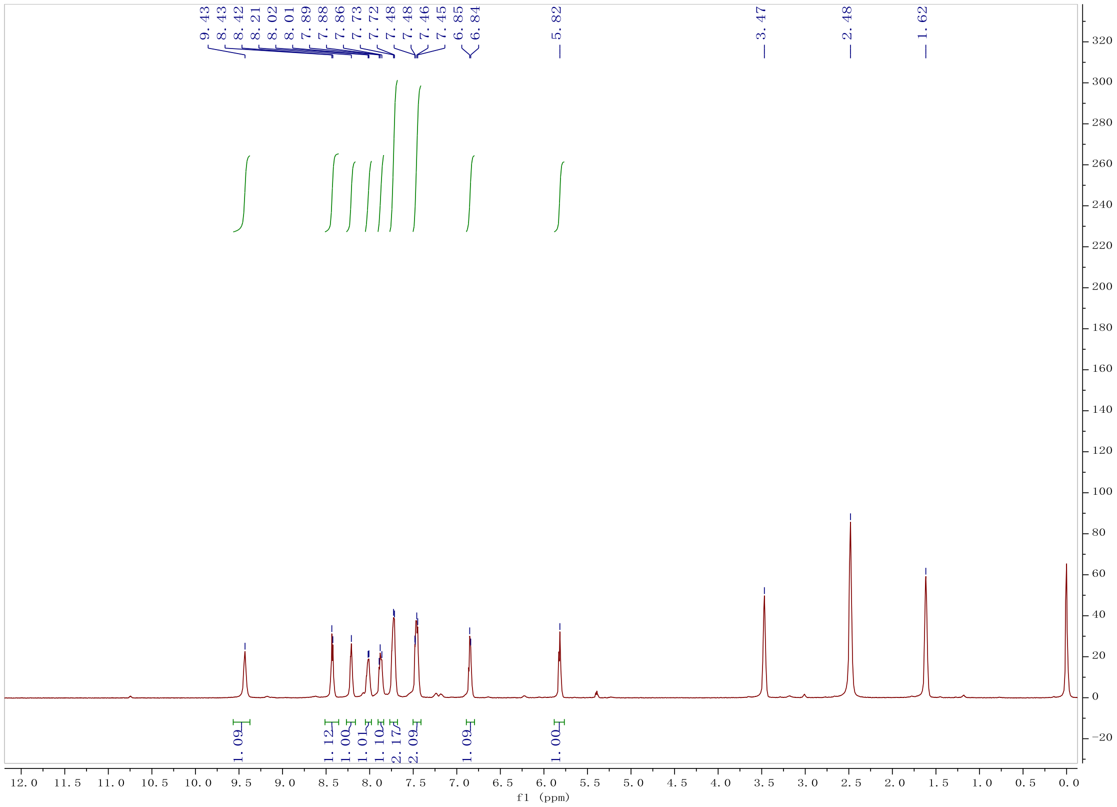
9.43
8.43
8.42
8.21
8.02
8.01
7.89
7.88
7.86
7.73
7.72
7.48
7.48
7.46
7.45
6.85
6.84
5.82
3.47
2.48
1.62
1.09
1.12
1.00
1.01
1.10
2.17
2.09
1.09
1.00
f1 (ppm)

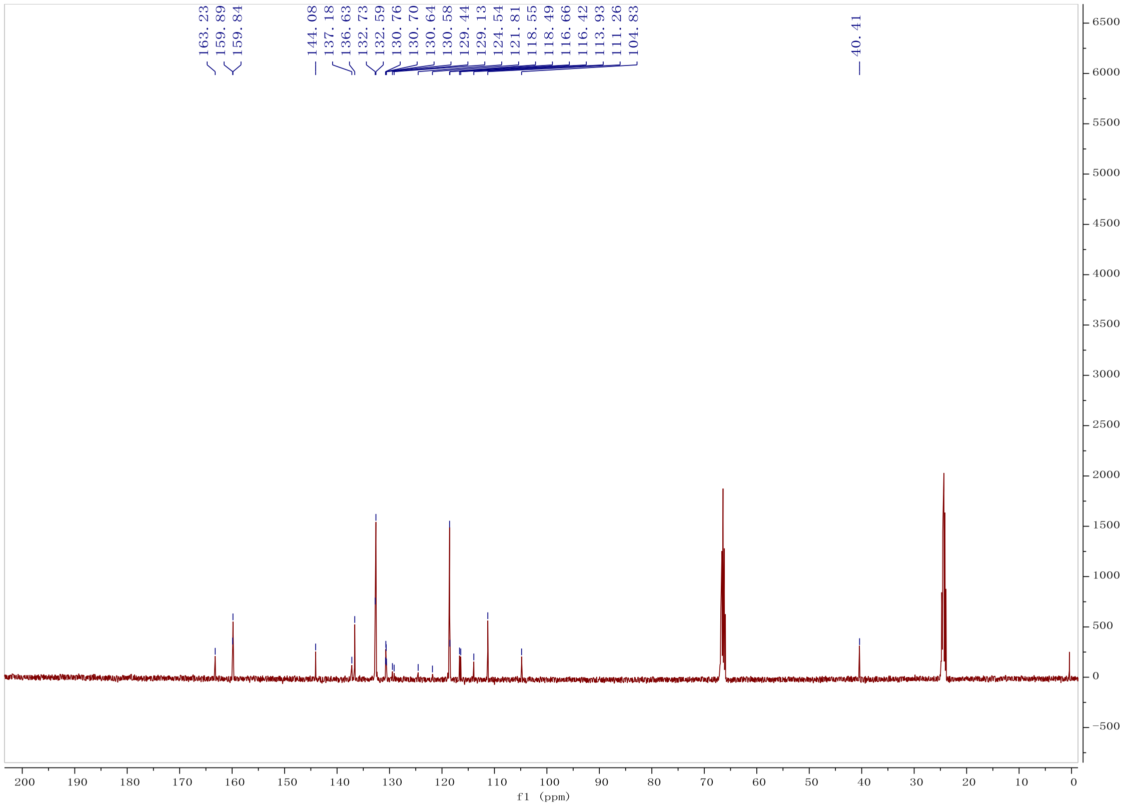
163.23
159.89
159.84
144.08
137.18
136.63
132.73
132.59
130.76
130.70
130.64
130.58
129.44
129.13
124.54
121.81
118.55
118.49
116.66
116.42
113.93
111.26
104.83
40.41
f1 (ppm)

7. HRMS, ^{1}H-NMR and ^{13}C NMR spectrum of A7

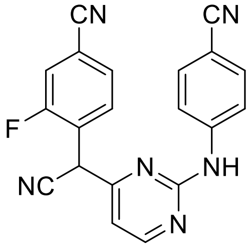

Chemical Formula: $C_{20}H_{11}FN_6$
Exact Mass: 354.1029
A7

Display Report

Analysis Info

Analysis Name	D:\Data\data\2019\20200102LTT-G-A7_RB3_01_3133.d	Acquisition Date	1/8/2020 10:40:33 AM
Method	MS-2MIN-NEG.m	Operator	BDAL@DE
Sample Name	20200102LTT-G-A7	Instrument	compact 8255754.20127
Comment			

Acquisition Parameter

Source Type	ESI	Ion Polarity	Negative	Set Nebulizer	2.0 Bar
Focus	Active	Set Capillary	2800 V	Set Dry Heater	200 °C
Scan Begin	50 m/z	Set End Plate Offset	-500 V	Set Dry Gas	8.0 l/min
Scan End	1500 m/z	Set Charging Voltage	2000 V	Set Divert Valve	Waste
		Set Corona	0 nA	Set APCI Heater	0 °C

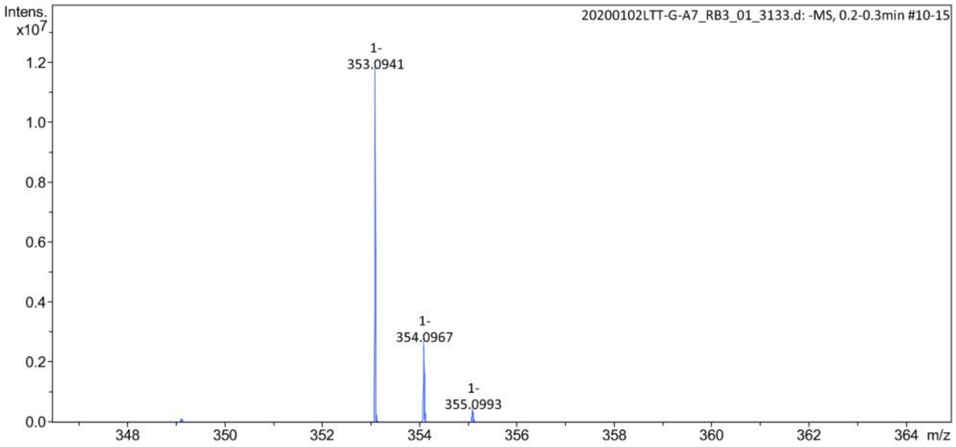

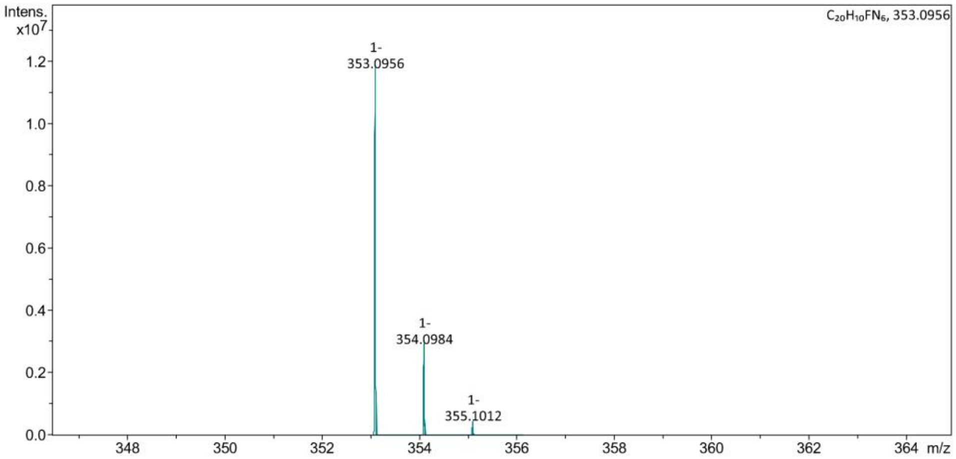

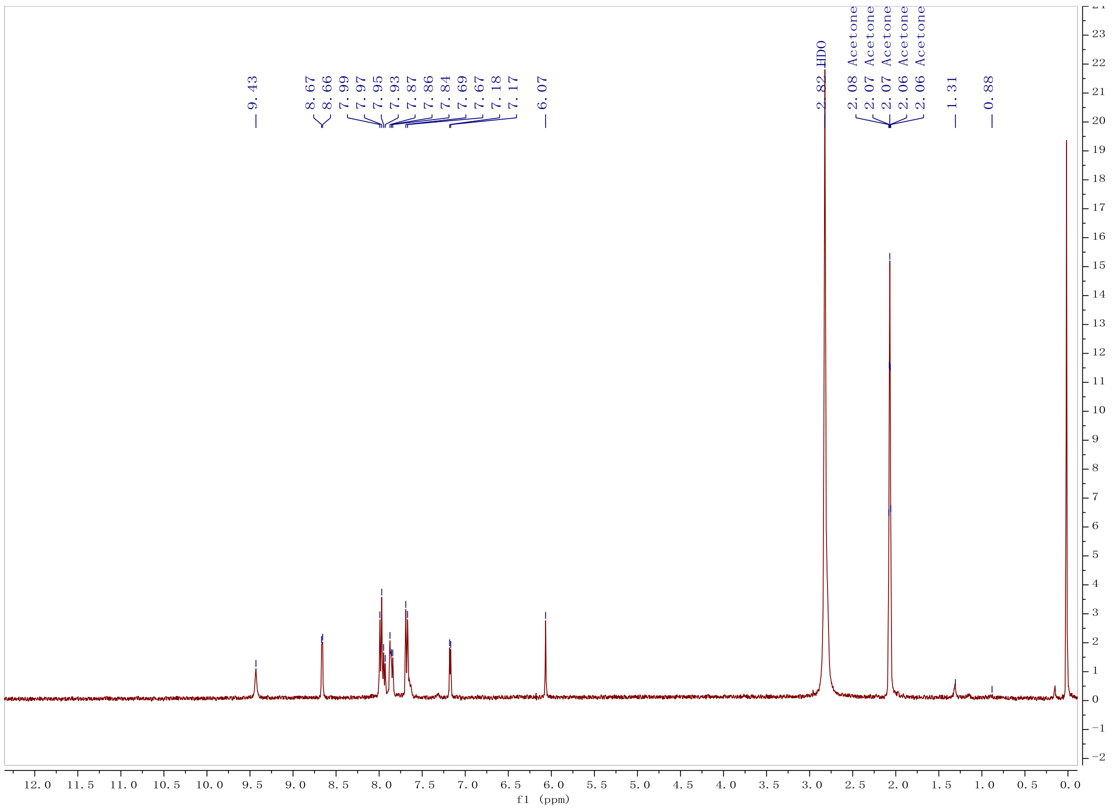
9.43
8.67
8.66
7.99
7.97
7.95
7.93
7.87
7.86
7.84
7.69
7.67
7.18
7.17
6.07
2.82 HDO
2.08 Acetone
2.07 Acetone
2.07 Acetone
2.06 Acetone
2.06 Acetone
1.31
0.88
f1 (ppm)

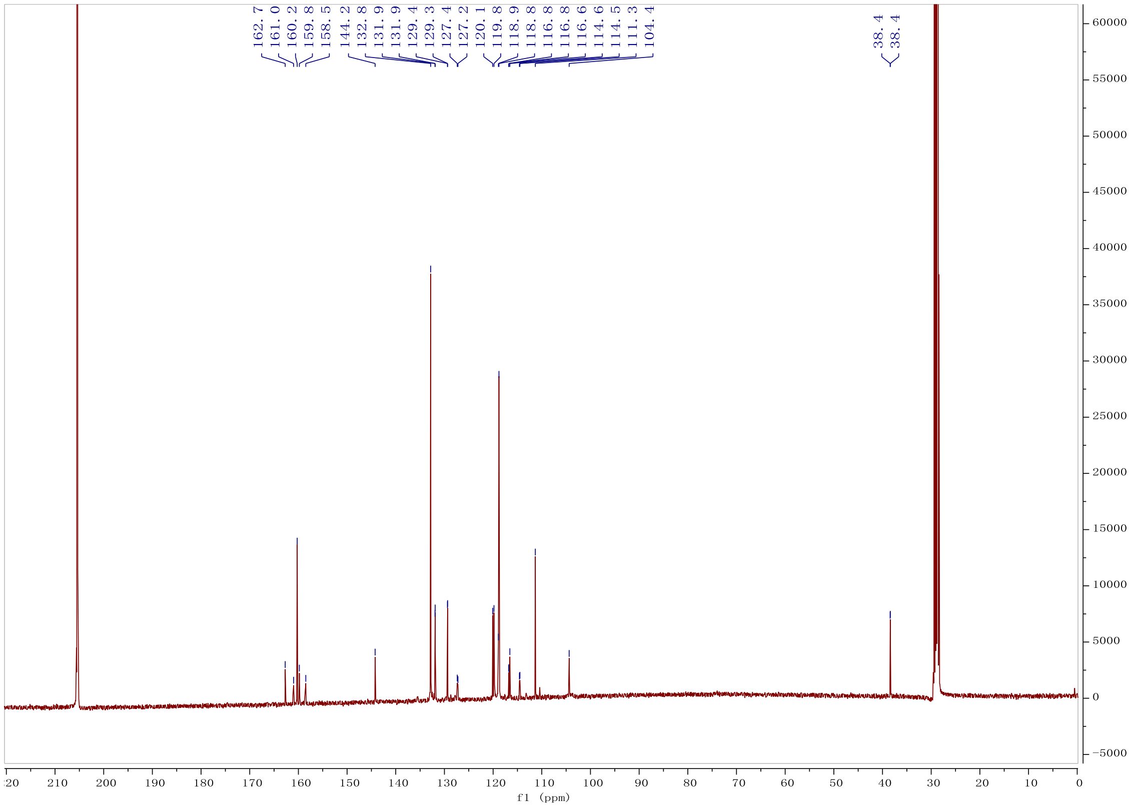
162.7
161.0
160.2
159.8
158.5
144.2
132.8
131.9
131.9
129.4
129.3
127.4
127.2
120.1
119.8
118.9
118.8
116.8
116.8
116.6
114.6
114.5
111.3
104.4
38.4
38.4
f1 (ppm)

8. HRMS, HPLC, ^{1}H-NMR and ^{13}C NMR spectrum of A8

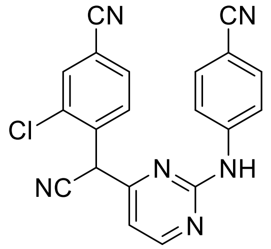

Chemical Formula: $C_{20}H_{11}ClN_6$
Exact Mass: 370.0734

A8

Display Report

Analysis Info

Analysis Name	D:\Data\data\2019\20200102LTT-H-A8_RB4_01_3119.d	Acquisition Date	1/8/2020 9:14:40 AM
Method	MS-2MIN-POS.m	Operator	BDAL@DE
Sample Name	20200102LTT-H-A8	Instrument	compact 8255754.20127
Comment			

Acquisition Parameter

Source Type	ESI	Ion Polarity	Positive	Set Nebulizer	2.0 Bar
Focus	Active	Set Capillary	4500 V	Set Dry Heater	200 °C
Scan Begin	50 m/z	Set End Plate Offset	-500 V	Set Dry Gas	8.0 l/min
Scan End	3000 m/z	Set Charging Voltage	2000 V	Set Divert Valve	Waste
		Set Corona	0 nA	Set APCI Heater	0 °C

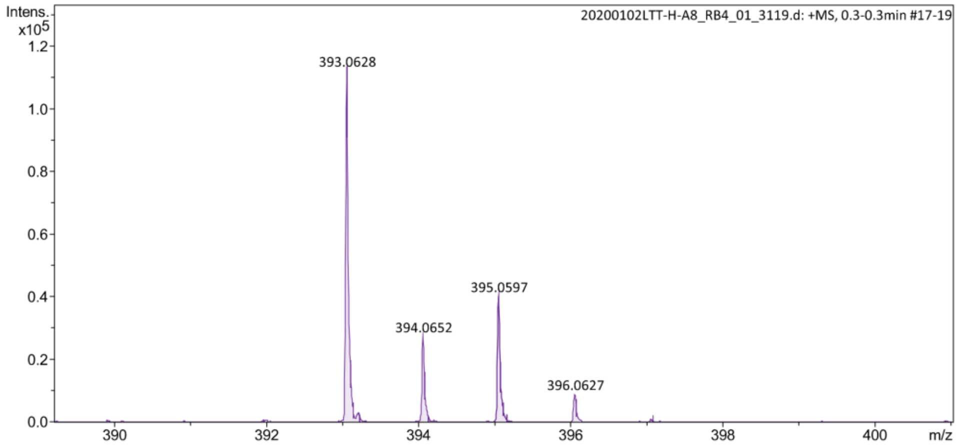

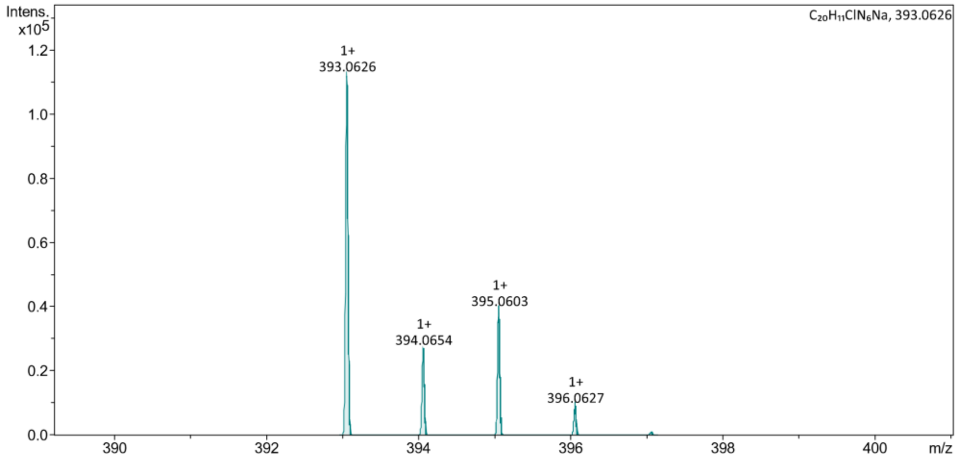

20200102LTT-H-A8_RB4_01_3119.d

Bruker Compass DataAnalysis 4.3 printed: 1/8/2020 10:34:27 AM by: BDAL@DE Page 1 of 1

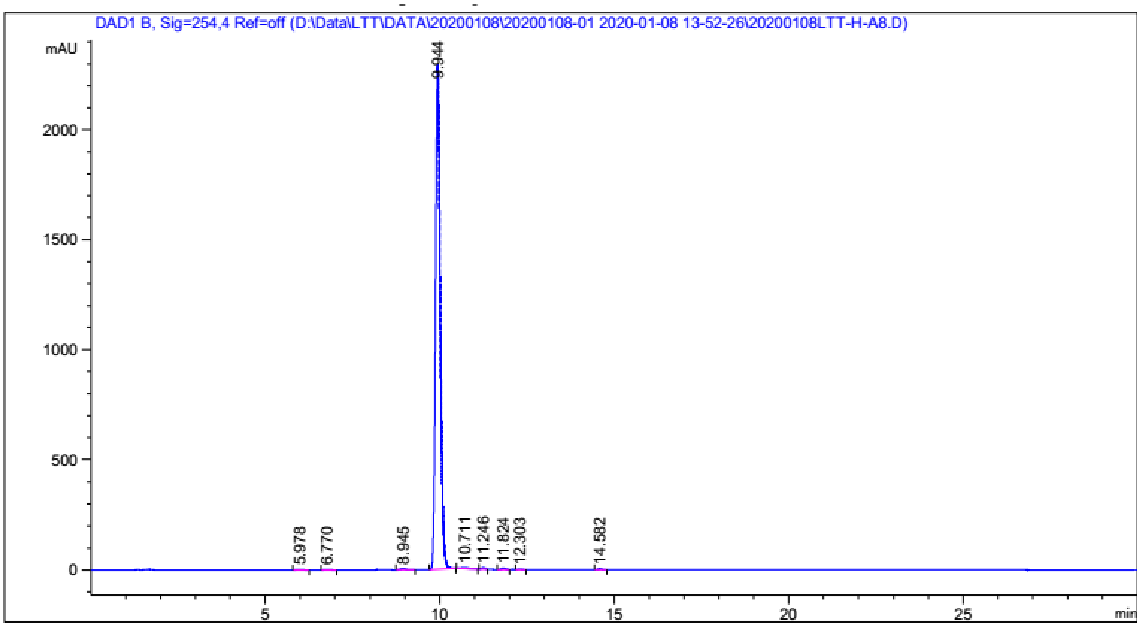

Peak #	RetTime [min]	Type	Width [min]	Area [mAU*s]	Height [mAU]	Area %
1	5.978	BB	0.1252	16.37985	1.55146	0.0764
2	6.770	BB	0.1270	11.31786	1.06891	0.0528
3	8.945	BB	0.1298	54.19859	5.50844	0.2529
4	9.944	BB	0.1408	2.11745e4	2291.52100	98.7928
5	10.711	BB	0.1592	59.67686	4.48590	0.2784
6	11.246	BB	0.0934	30.08104	4.98219	0.1403
7	11.824	BV	0.1090	48.05025	5.41818	0.2242
8	12.303	BB	0.1070	12.32087	1.36950	0.0575
9	14.582	BB	0.0999	26.71183	3.75716	0.1246

Totals : 2.14332e4 2319.66274

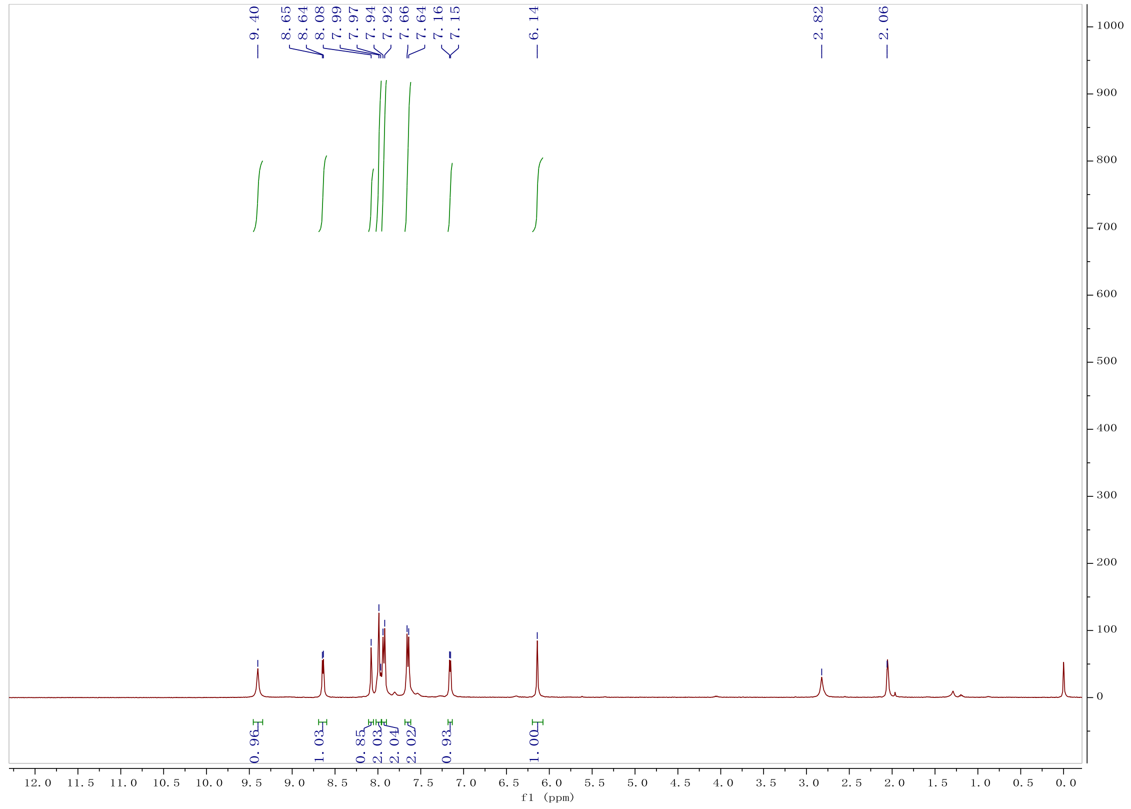
9.40
8.65
8.64
8.08
7.99
7.97
7.94
7.92
7.66
7.64
7.16
7.15
6.14
2.82
2.06
0.96
1.03
0.85
2.03
2.04
2.02
0.93
1.00
f1 (ppm)

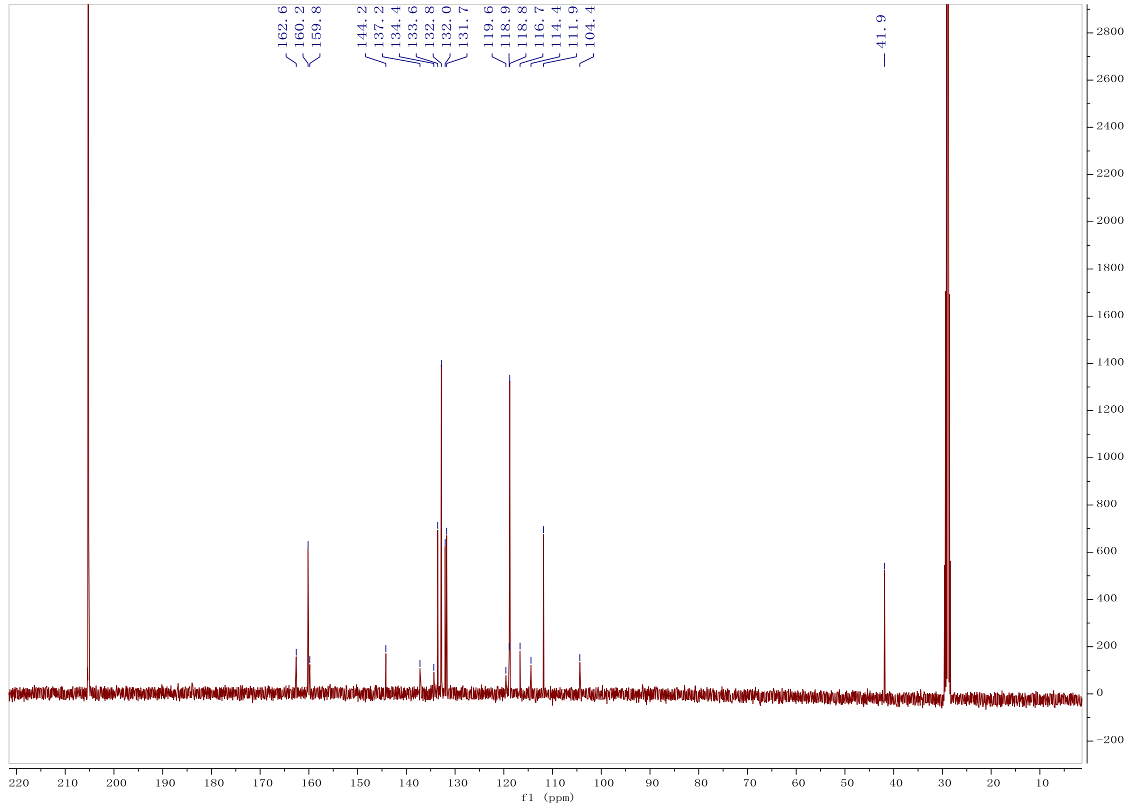
162.6
160.2
159.8
144.2
137.2
134.4
133.6
132.8
132.0
131.7
119.6
118.9
118.8
116.7
114.4
111.9
104.4
41.9
f1 (ppm)

9. HRMS, HPLC, ^{1}H-NMR and ^{13}C NMR spectrum of A9

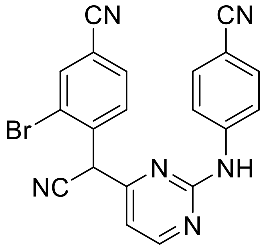

Chemical Formula: $C_{20}H_{11}BrN_6$
Exact Mass: 414.0229
A9

Display Report

Analysis Info

Analysis Name	D:\Data\data\2019\20200107LTT-I-A9_RB6_01_3135.d	Acquisition Date	1/8/2020 10:50:52 AM
Method	MS-2MIN-NEG.m	Operator	BDAL@DE
Sample Name	20200107LTT-I-A9	Instrument	compact 8255754.20127
Comment			

Acquisition Parameter

Source Type	ESI	Ion Polarity	Negative	Set Nebulizer	2.0 Bar
Focus	Active	Set Capillary	2800 V	Set Dry Heater	200 °C
Scan Begin	50 m/z	Set End Plate Offset	-500 V	Set Dry Gas	8.0 l/min
Scan End	1500 m/z	Set Charging Voltage	2000 V	Set Divert Valve	Waste
		Set Corona	0 nA	Set APCI Heater	0 °C

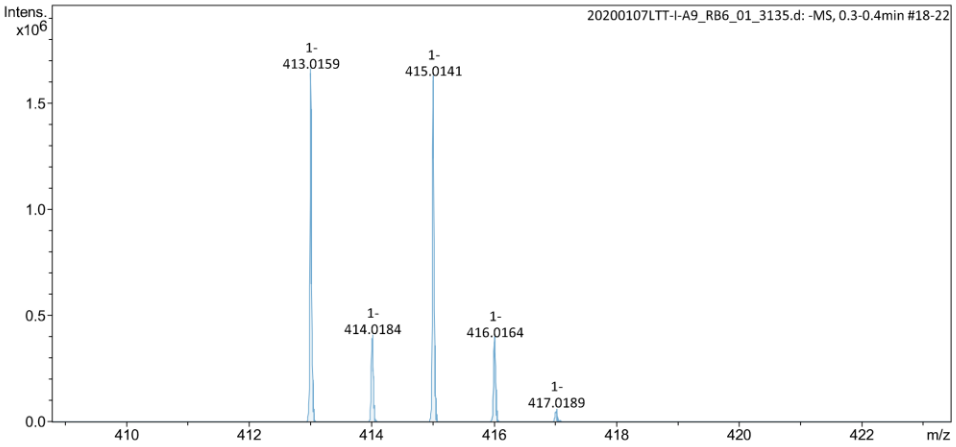

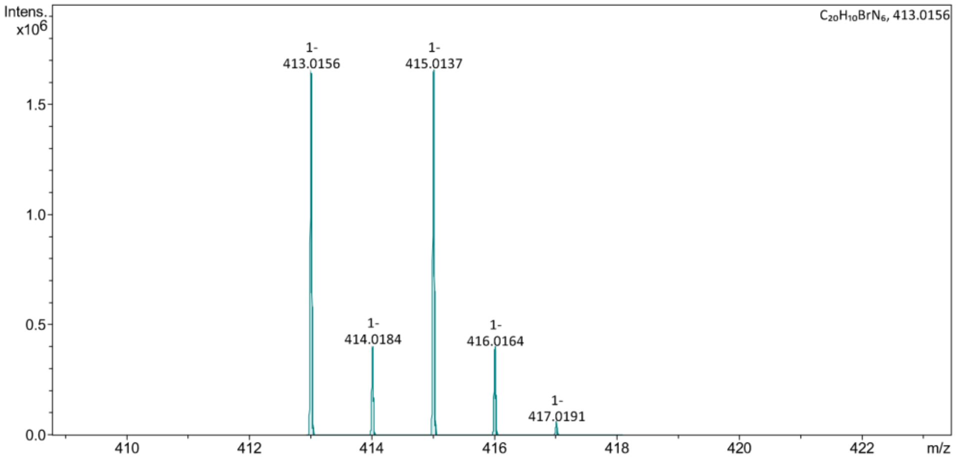

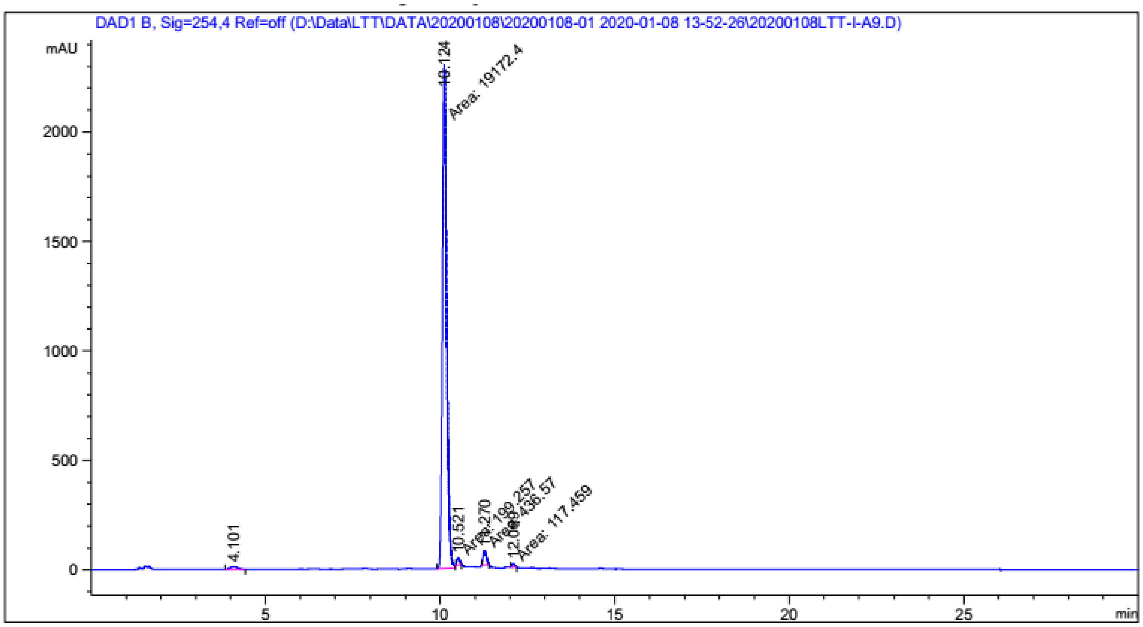

Peak #	RetTime [min]	Type	Width [min]	Area [mAU*s]	Height [mAU]	Area %
1	4.101	BB	0.1742	181.05978	12.74475	0.9005
2	10.124	MF	0.1388	1.91724e4	2301.56079	95.3531
3	10.521	MM	0.0996	199.25746	33.35526	0.9910
4	11.270	MM	0.1101	436.56985	66.08507	2.1713
5	12.089	MM	0.1070	117.45864	18.28863	0.5842

Totals : 2.01067e4 2432.03450

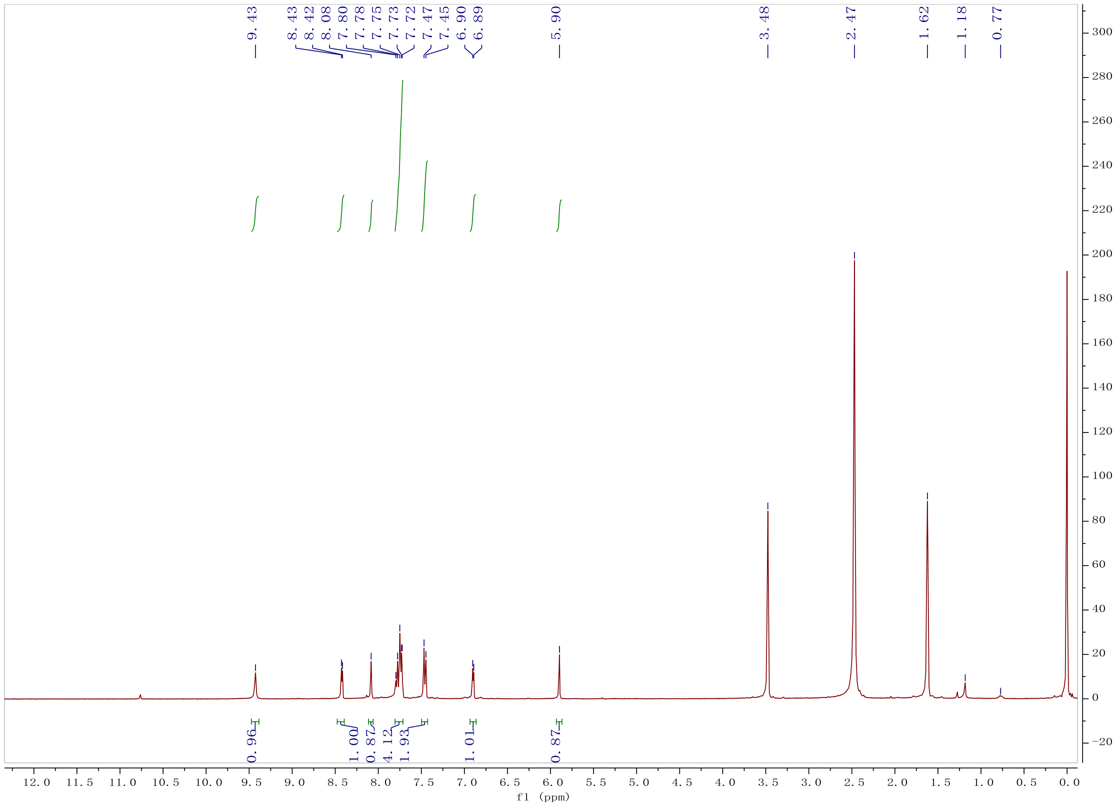
9.43
8.43
8.42
8.08
7.80
7.78
7.75
7.73
7.72
7.47
7.45
6.90
6.89
5.90
3.48
2.47
1.62
1.18
0.77
0.96
1.00
0.87
4.12
1.93
1.01
0.87
f1 (ppm)

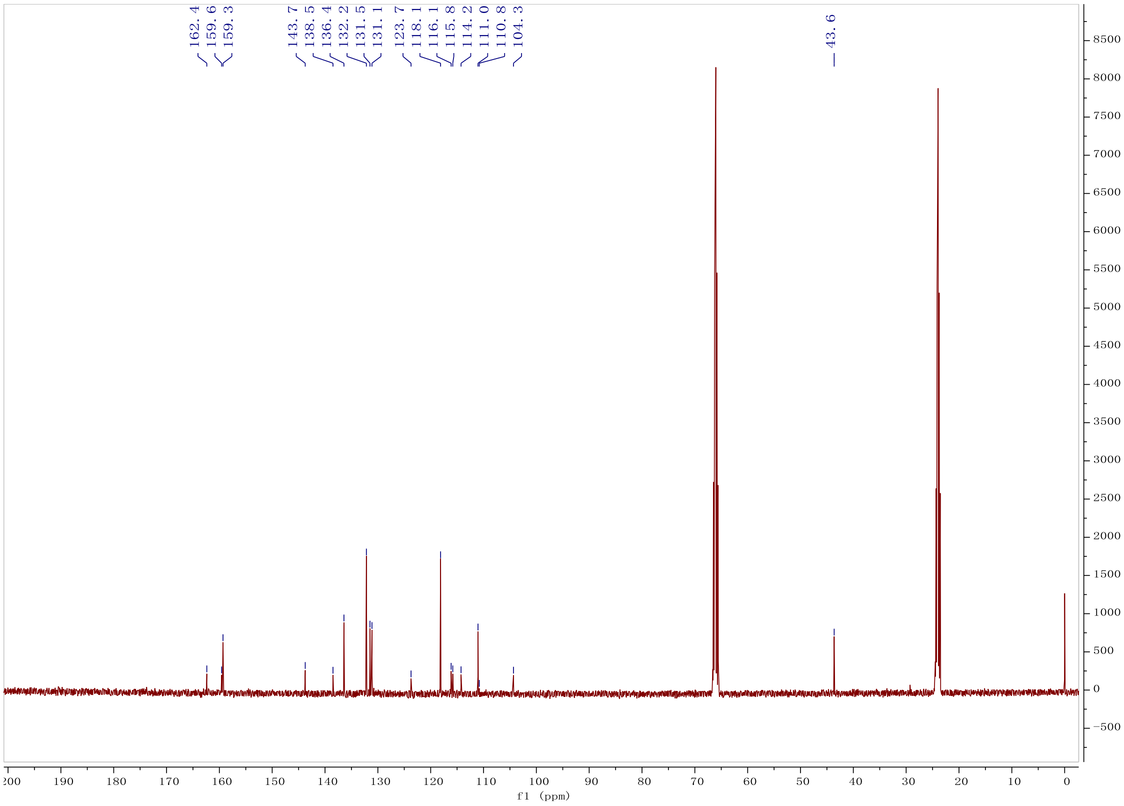
162.4
159.6
159.3
143.7
138.5
136.4
132.2
131.5
131.1
123.7
118.1
116.1
115.8
114.2
111.0
110.8
104.3
43.6
f1 (ppm)

10. HRMS, HPLC, ^{1}H-NMR and ^{13}C NMR spectrum of A10

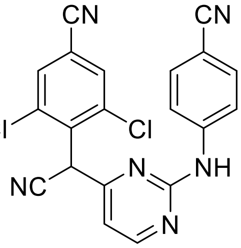

Chemical Formula: $C_{20}H_{10}Cl_2N_6$
Exact Mass: 404.0344
A10

Display Report

Analysis Info

Analysis Name	D:\Data\data\2019\20200107LTT-J-A10_RA2_01_3125.d	Acquisition Date	1/8/2020 9:31:15 AM
Method	MS-2MIN-POS.m	Operator	BDAL@DE
Sample Name	20200107LTT-J-A10	Instrument	compact 8255754.20127
Comment			

Acquisition Parameter

Source Type	ESI	Ion Polarity	Positive	Set Nebulizer	2.0 Bar
Focus	Active	Set Capillary	4500 V	Set Dry Heater	200 °C
Scan Begin	50 m/z	Set End Plate Offset	-500 V	Set Dry Gas	8.0 l/min
Scan End	3000 m/z	Set Charging Voltage	2000 V	Set Divert Valve	Waste
		Set Corona	0 nA	Set APCI Heater	0 °C

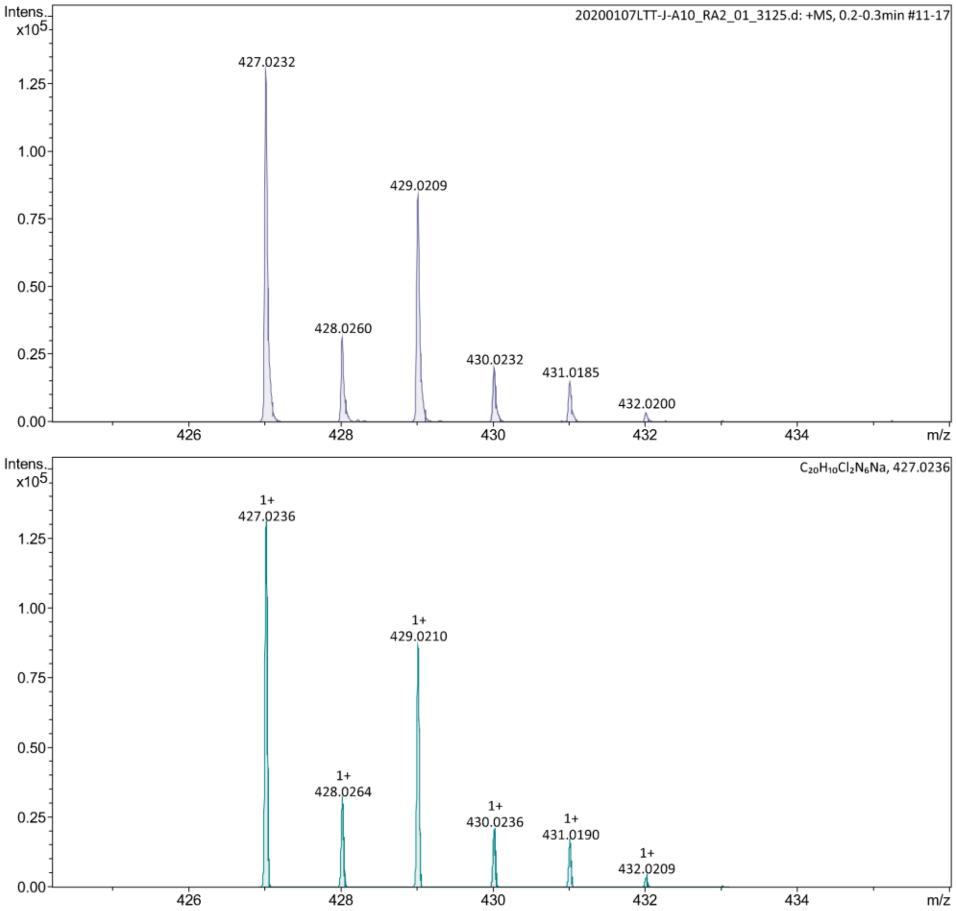

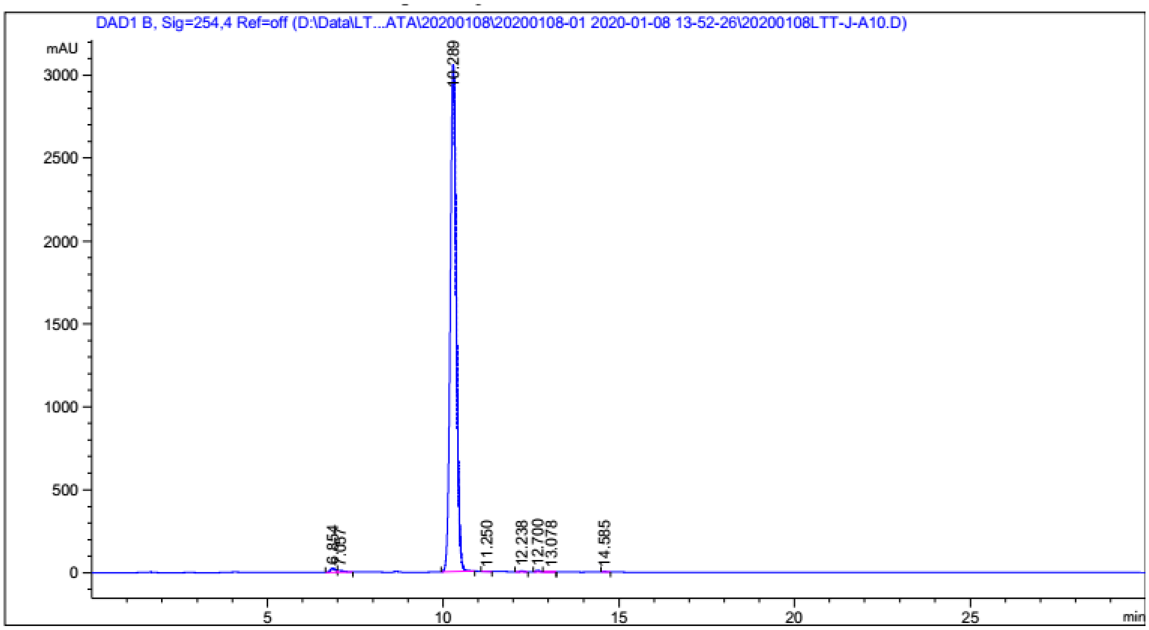

Peak #	RetTime [min]	Type	Width [min]	Area [mAU*s]	Height [mAU]	Area %
1	6.854	BV	0.1505	248.43451	24.34615	0.6766
2	7.057	VB	0.1460	126.28705	11.51151	0.3439
3	10.289	BB	0.1611	3.61590e4	3055.70459	98.4767
4	11.250	BV	0.1242	23.51675	2.23517	0.0640
5	12.238	BB	0.1079	55.24250	7.41486	0.1504
6	12.700	BV R	0.1108	70.39449	9.30520	0.1917
7	13.078	VB E	0.1137	10.66923	1.11939	0.0291
8	14.585	VB	0.0948	24.78256	3.47116	0.0675
Totals :				3.67183e4	3115.10803	

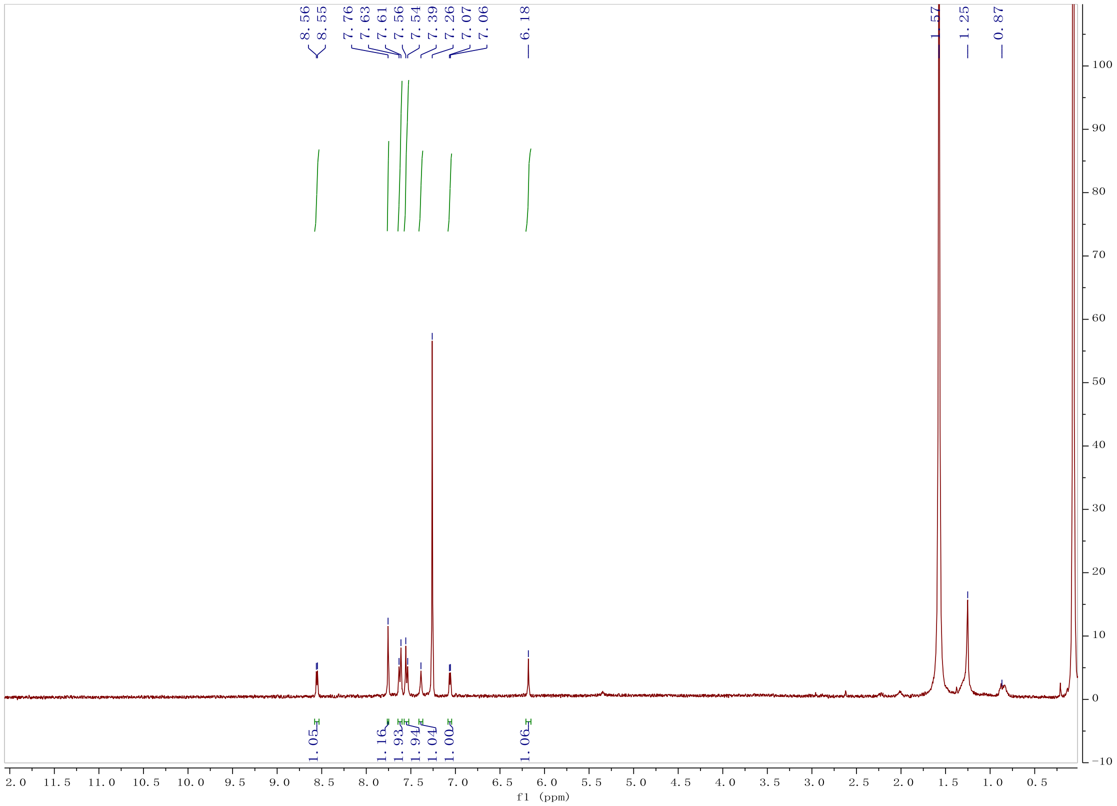
8.56
8.55
7.76
7.63
7.61
7.56
7.54
7.39
7.26
7.07
7.06
6.18
1.57
1.25
0.87
1.05
1.16
1.93
1.94
1.04
1.00
1.06
f1 (ppm)

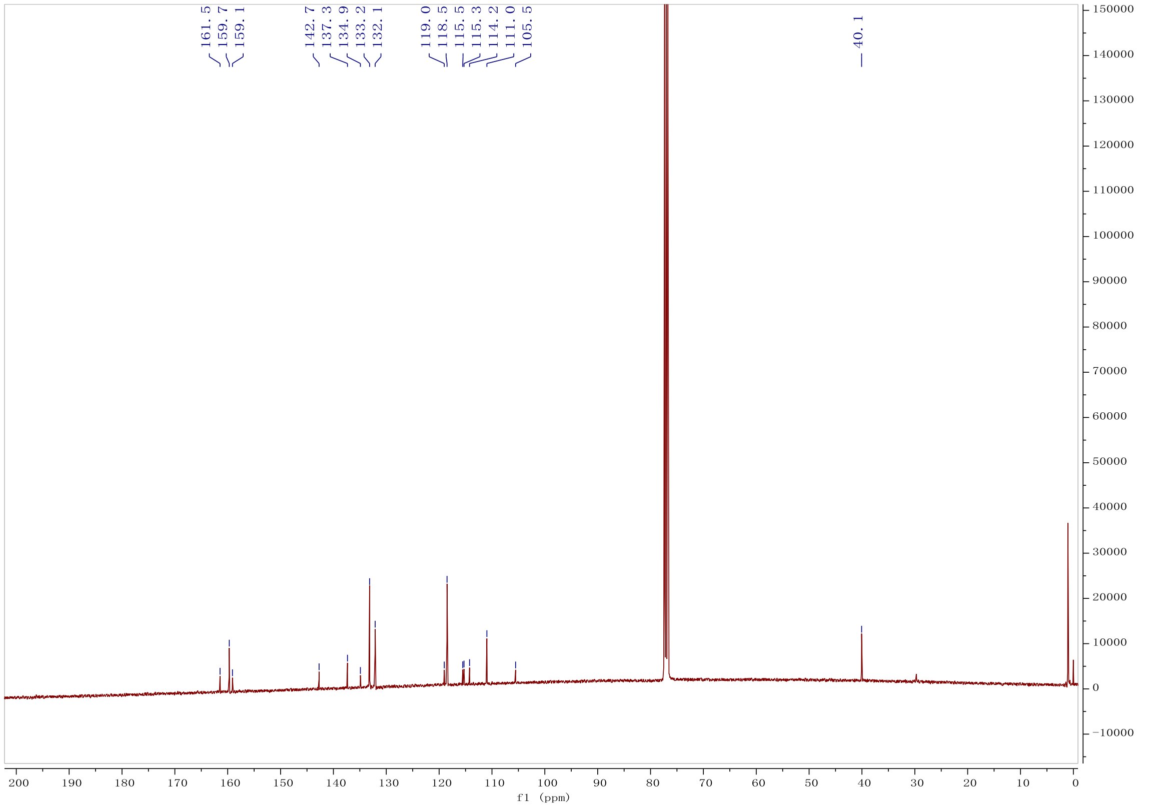
161.5
159.7
159.1
142.7
137.3
134.9
133.2
132.1
119.0
118.5
115.5
115.3
114.2
111.0
105.5
40.1
f1 (ppm)

11. HRMS, HPLC, ^{1}H-NMR and ^{13}C NMR spectrum of A11

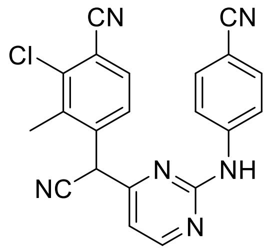

Chemical Formula: $C_{21}H_{13}ClN_6$
Exact Mass: 384.0890
A11

Display Report

Analysis Info

Analysis Name	D:\Data\data\2019\20200107LTT-K-A11_RA6_01_3129.d	Acquisition Date	1/8/2020 9:42:18 AM
Method	MS-2MIN-POS.m	Operator	BDAL@DE
Sample Name	20200107LTT-K-A11	Instrument	compact 8255754.20127
Comment			

Acquisition Parameter

Source Type	ESI	Ion Polarity	Positive	Set Nebulizer	2.0 Bar
Focus	Active	Set Capillary	4500 V	Set Dry Heater	200 °C
Scan Begin	50 m/z	Set End Plate Offset	-500 V	Set Dry Gas	8.0 l/min
Scan End	3000 m/z	Set Charging Voltage	2000 V	Set Divert Valve	Waste
		Set Corona	0 nA	Set APCI Heater	0 °C

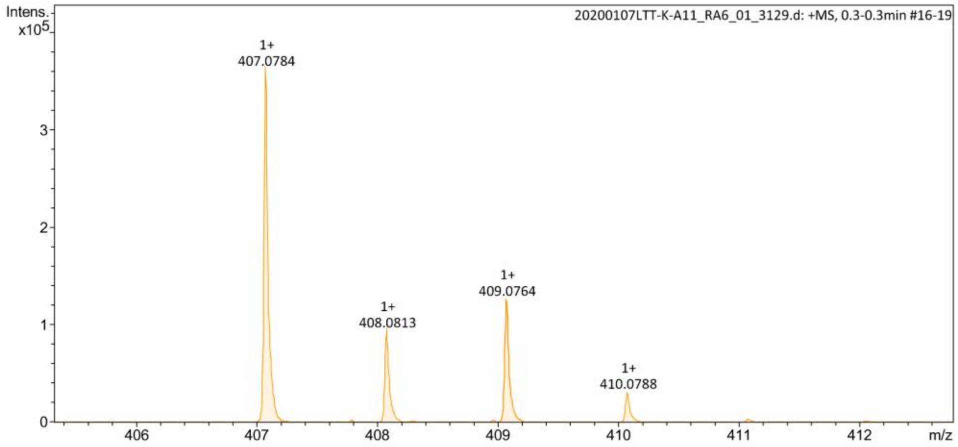

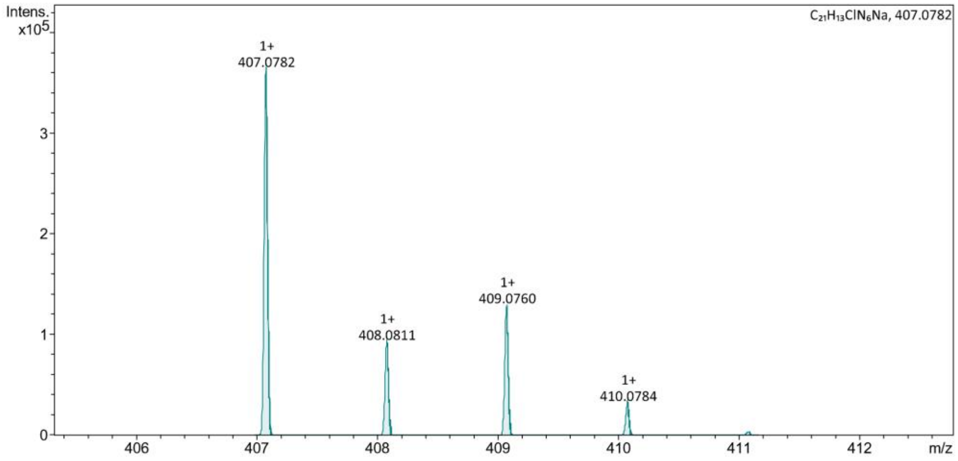

20200107LTT-K-A11_RA6_01_3129.d

Bruker Compass DataAnalysis 4.3 printed: 1/8/2020 10:20:22 AM by: BDAL@DE Page 1 of 1

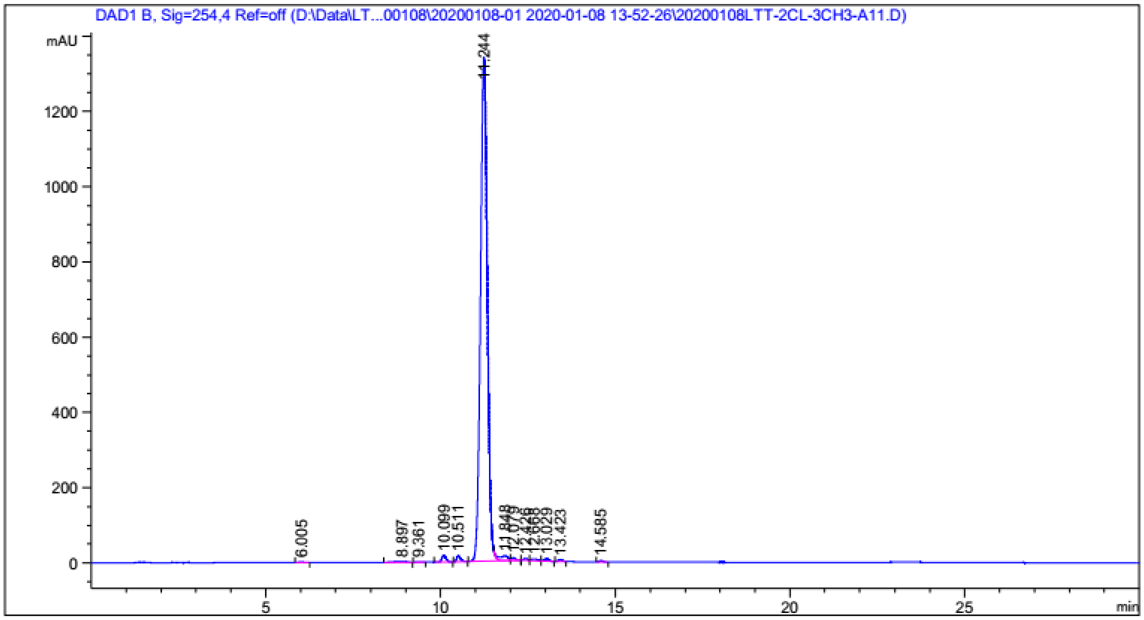

Peak #	RetTime [min]	Type	Width [min]	Area [mAU*s]	Height [mAU]	Area %
1	6.005	BB	0.1196	14.22625	1.40498	0.0751
2	8.897	BB	0.2644	59.53582	2.64244	0.3144
3	9.361	BB	0.1241	11.07698	1.06307	0.0585
4	10.099	BB	0.1249	157.12971	18.62922	0.8297
5	10.511	BB	0.1179	128.00908	15.98117	0.6760
6	11.244	BV R	0.2069	1.80643e4	1339.21216	95.3902
7	11.848	VV E	0.2054	213.76898	12.66510	1.1288
8	12.079	VB E	0.1134	56.25141	6.98854	0.2970
9	12.426	BV	0.1036	37.58985	5.06667	0.1985
10	12.668	VV	0.1520	59.38890	4.68175	0.3136
11	13.029	VB	0.1201	65.99423	6.57537	0.3485
12	13.423	BB	0.1260	42.81383	4.80503	0.2261
13	14.585	BB	0.1025	27.17659	3.70871	0.1435

Totals : 1.89373e4 1423.42422

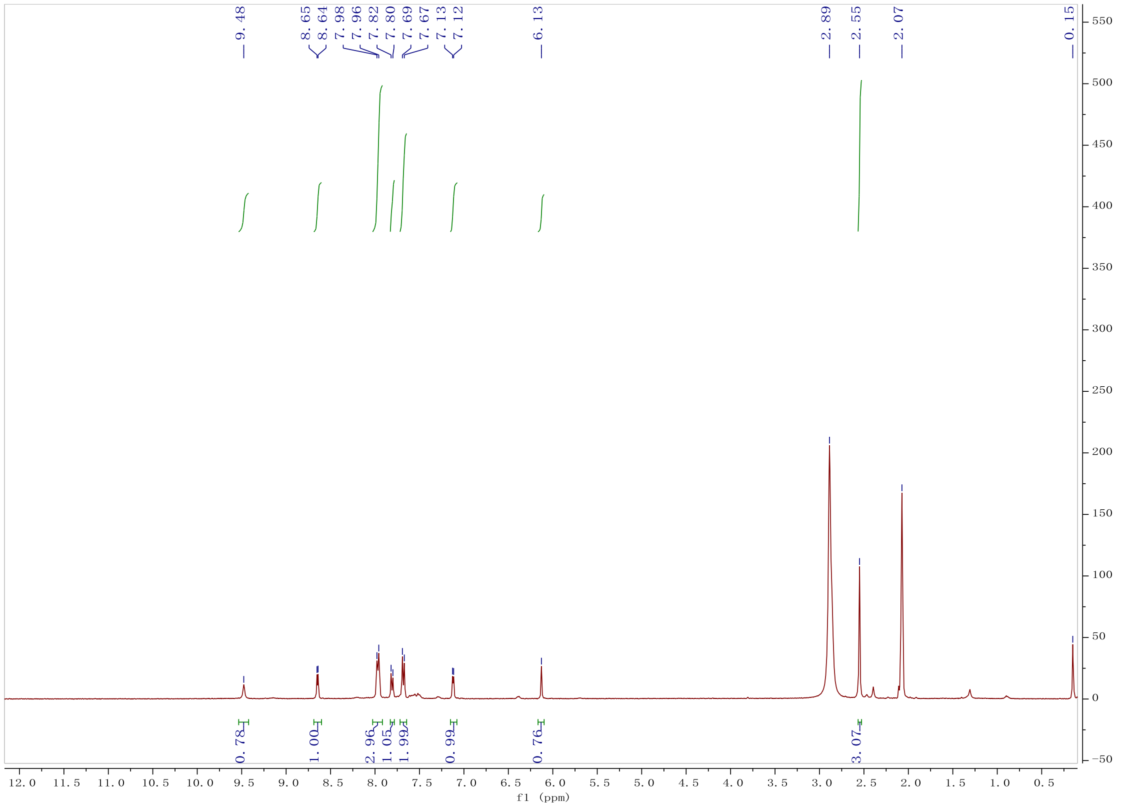

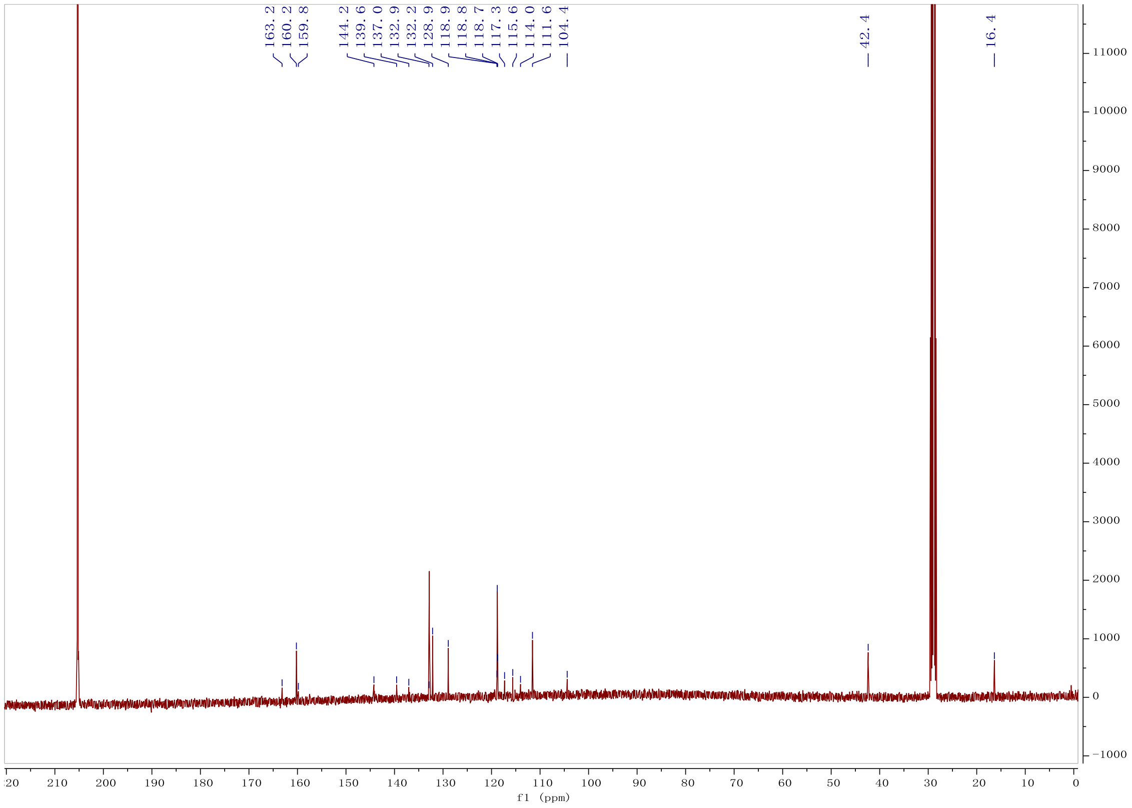

12. HRMS, HPLC, ^{1}H-NMR and ^{13}C NMR spectrum of A12

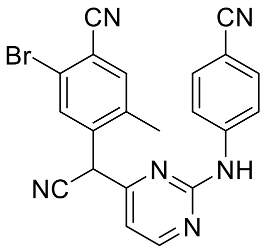

Chemical Formula: $C_{21}H_{13}BrN_6$
Exact Mass: 428.0385
A12

Display Report

Analysis Info

Analysis Name	D:\Data\data\2019\20200107LTT-I-A12_RA5_01_3134.d	Acquisition Date	1/8/2020 10:43:20 AM
Method	MS-2MIN-NEG.m	Operator	BDAL@DE
Sample Name	20200107LTT-I-A12	Instrument	compact 8255754.20127
Comment			

Acquisition Parameter

Source Type	ESI	Ion Polarity	Negative	Set Nebulizer	2.0 Bar
Focus	Active	Set Capillary	2800 V	Set Dry Heater	200 °C
Scan Begin	50 m/z	Set End Plate Offset	-500 V	Set Dry Gas	8.0 l/min
Scan End	1500 m/z	Set Charging Voltage	2000 V	Set Divert Valve	Waste
		Set Corona	0 nA	Set APCI Heater	0 °C

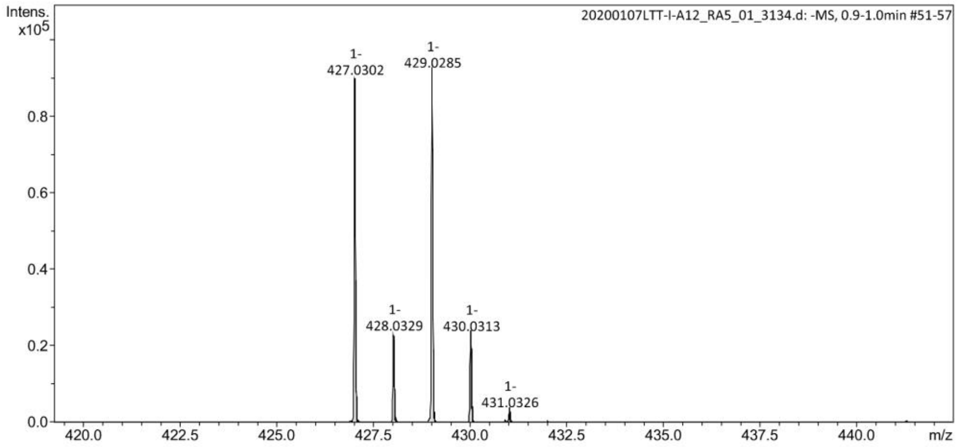

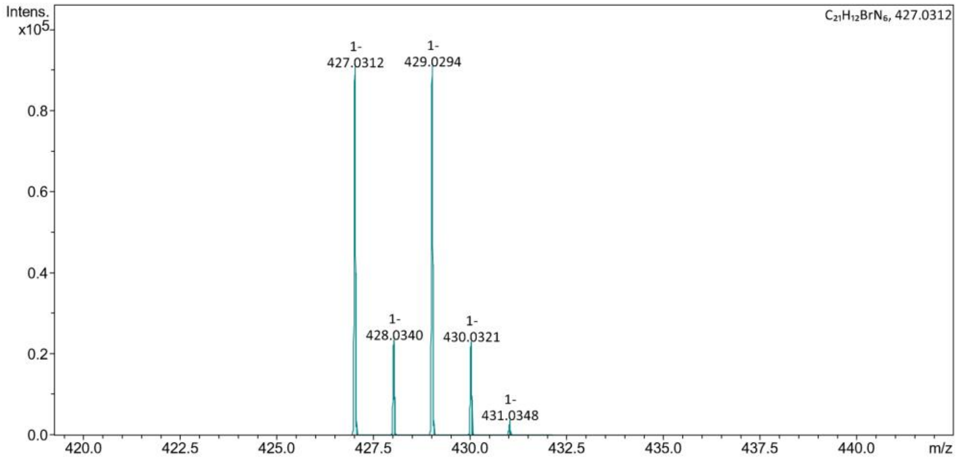

20200107LTT-I-A12_RA5_01_3134.d

Bruker Compass DataAnalysis 4.3 printed: 1/8/2020 10:49:31 AM by: BDAL@DE Page 1 of 1

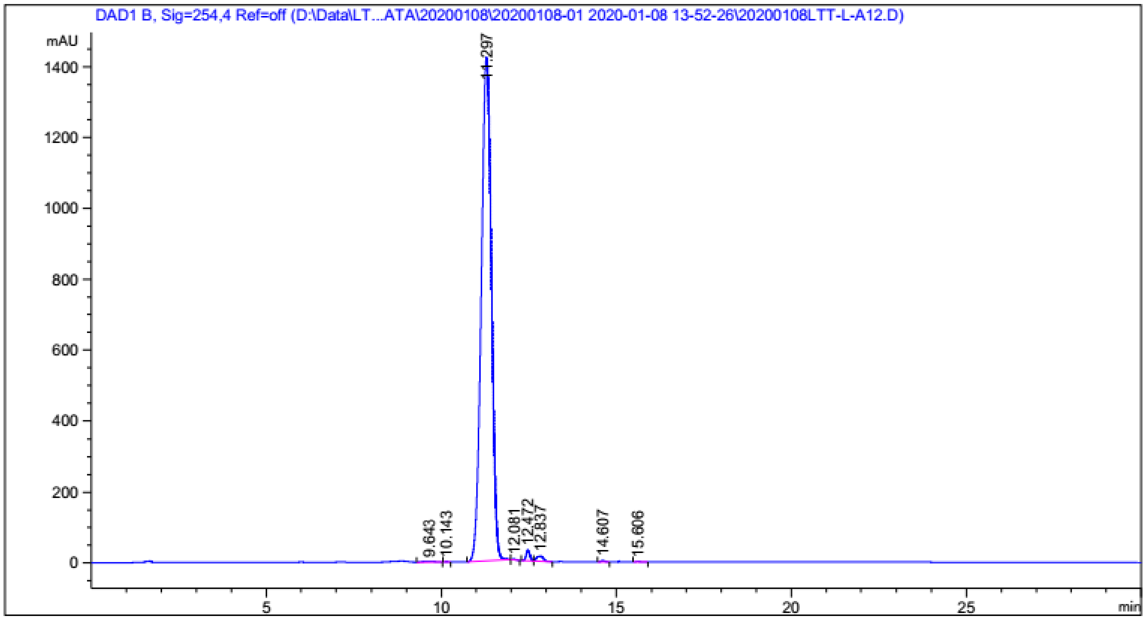

Peak #	RetTime [min]	Type	Width [min]	Area [mAU*s]	Height [mAU]	Area %
1	9.643	BB	0.2517	57.49035	2.68036	0.1989
2	10.143	BB	0.0787	6.69508	1.03903	0.0232
3	11.297	BB	0.3109	2.83736e4	1421.04199	98.1494
4	12.081	BB	0.0982	13.27098	1.66145	0.0459
5	12.472	BV	0.1136	230.55959	30.37538	0.7975
6	12.837	VB	0.1606	191.37198	14.12326	0.6620
7	14.607	BB	0.0953	25.30876	3.54343	0.0875
8	15.606	BB	0.1017	10.27722	1.21532	0.0356
Totals :				2.89086e4	1475.68022	

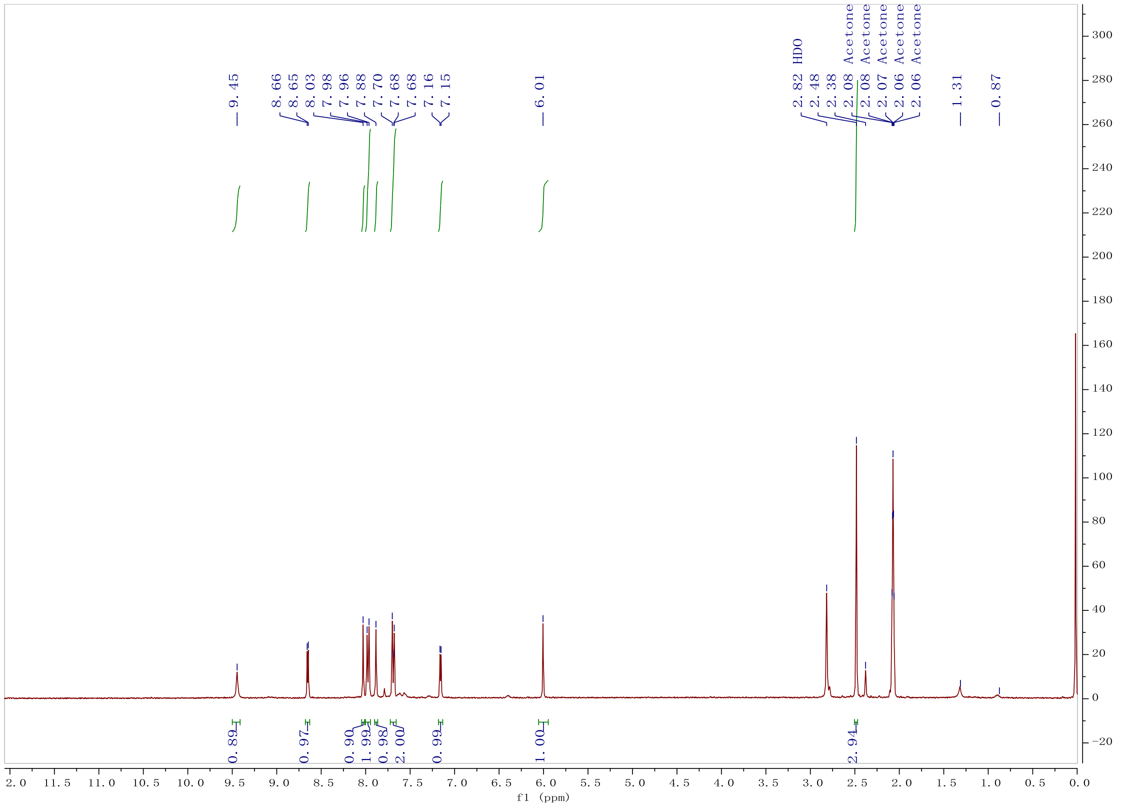
9.45
8.66
8.65
8.03
7.98
7.96
7.88
7.70
7.68
7.68
7.16
7.15
6.01
2.82 HDO
2.48
2.38
2.08 Acetone
2.08 Acetone
2.07 Acetone
2.06 Acetone
2.06 Acetone
1.31
0.87
0.89
0.97
0.90
1.99
0.98
2.00
0.99
1.00
2.94
f1 (ppm)

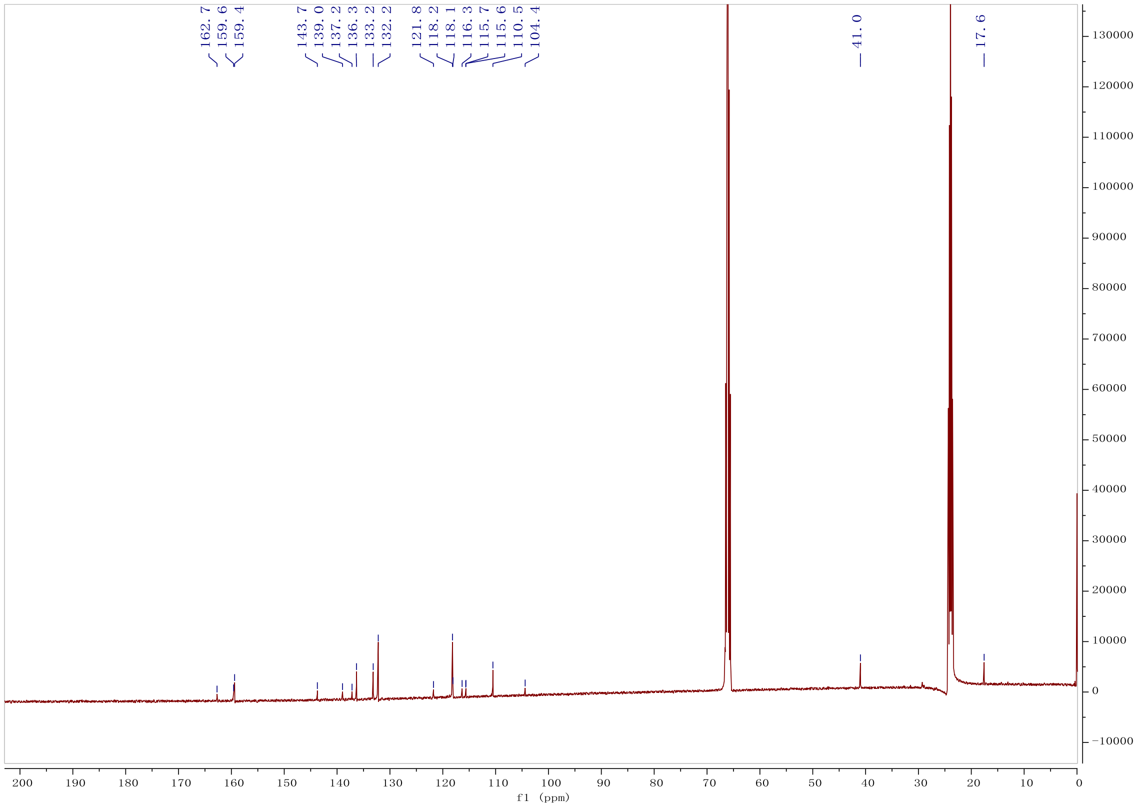
162.7
159.6
159.4
143.7
139.0
137.2
136.3
133.2
132.2
121.8
118.2
118.1
116.3
115.7
115.6
110.5
104.4
41.0
17.6
f1 (ppm)

13. HRMS, ^{1}H-NMR and ^{13}C NMR spectrum of B1

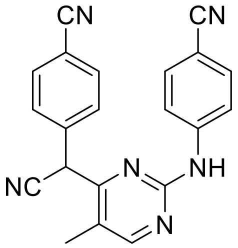

Chemical Formula: $C_{21}H_{14}N_6$
Exact Mass: 350.1280

B1

Display Report

Analysis Info

Analysis Name	D:\Data\data\2019\20191231LTTCH3-A1_RE2_01_3062.d	Acquisition Date	1/2/2020 19:01:07 PM
Method	MS-2MIN-POS.m	Operator	BDAL@DE
Sample Name	20191231LTTCH3-A1	Instrument	compact 8255754.20127
Comment			

Acquisition Parameter

Source Type	ESI	Ion Polarity	Positive	Set Nebulizer	2.0 Bar
Focus	Active	Set Capillary	4500 V	Set Dry Heater	200 °C
Scan Begin	50 m/z	Set End Plate Offset	-500 V	Set Dry Gas	8.0 l/min
Scan End	3000 m/z	Set Charging Voltage	2000 V	Set Divert Valve	Waste
		Set Corona	0 nA	Set APCI Heater	0 °C

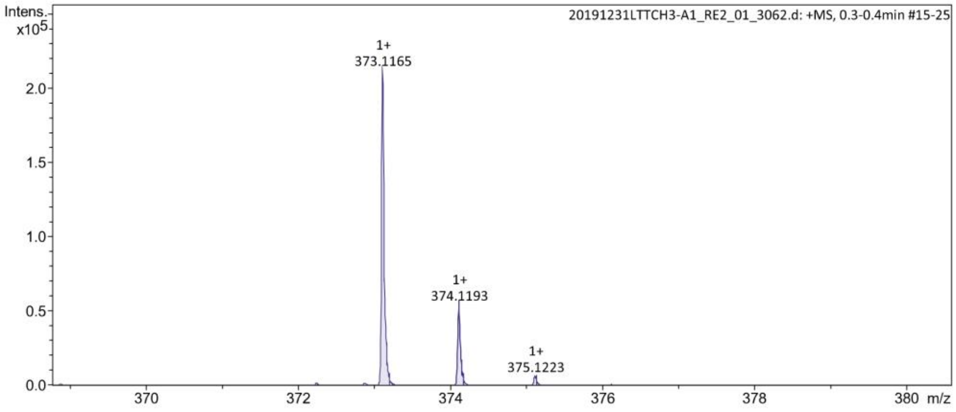

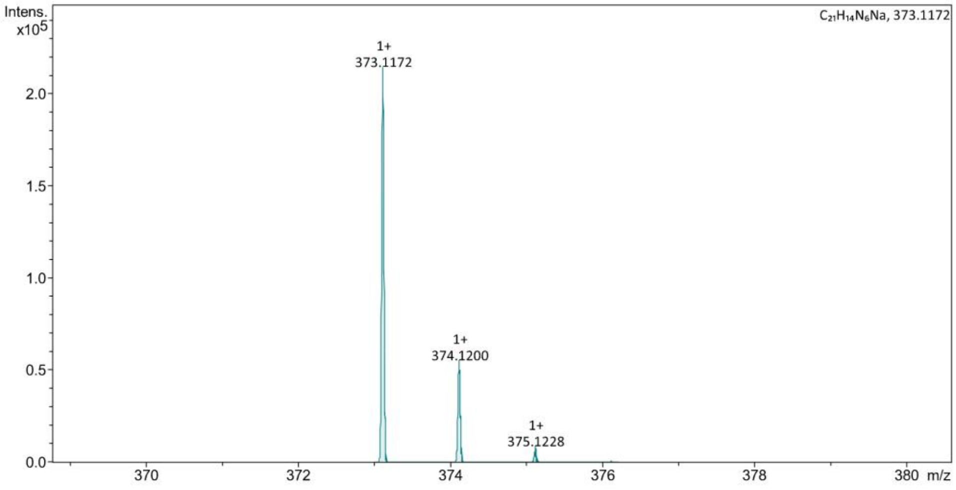

20191231LTTCH3-A1_RE2_01_3062.d

Bruker Compass DataAnalysis 4.3 printed: 1/2/2020 20:11:01 PM by: BDAL@DE Page 1 of 1

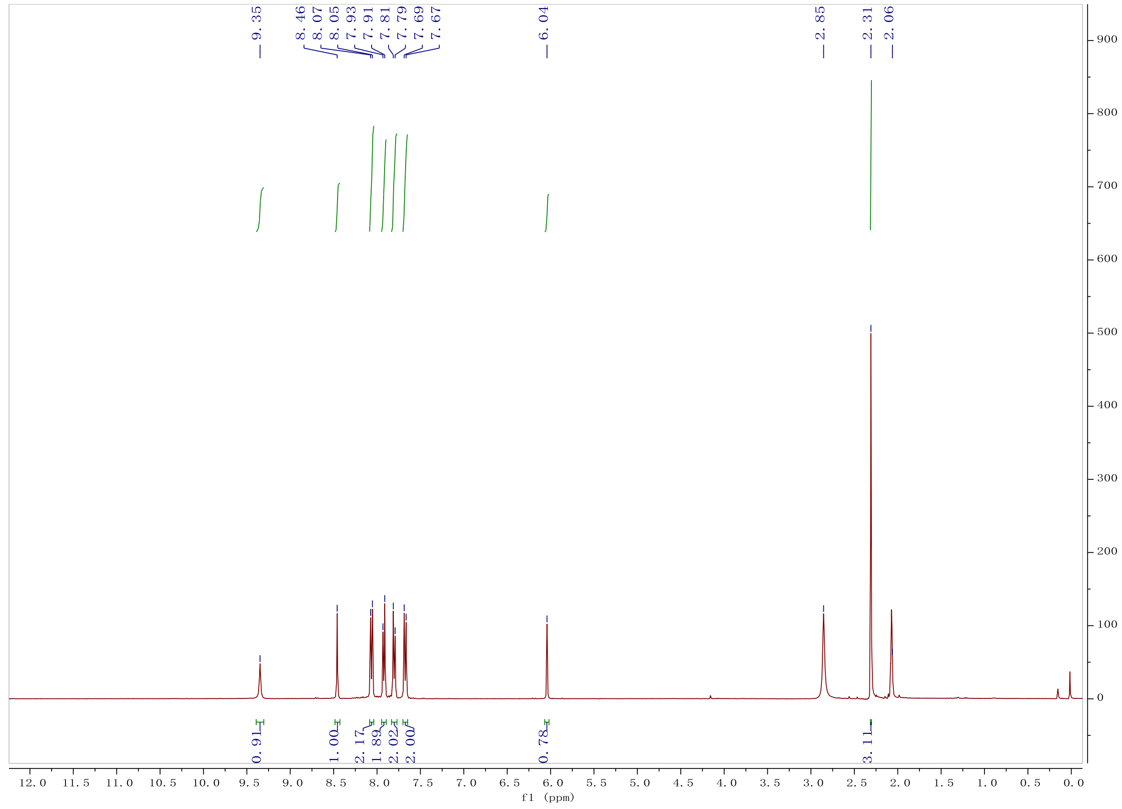

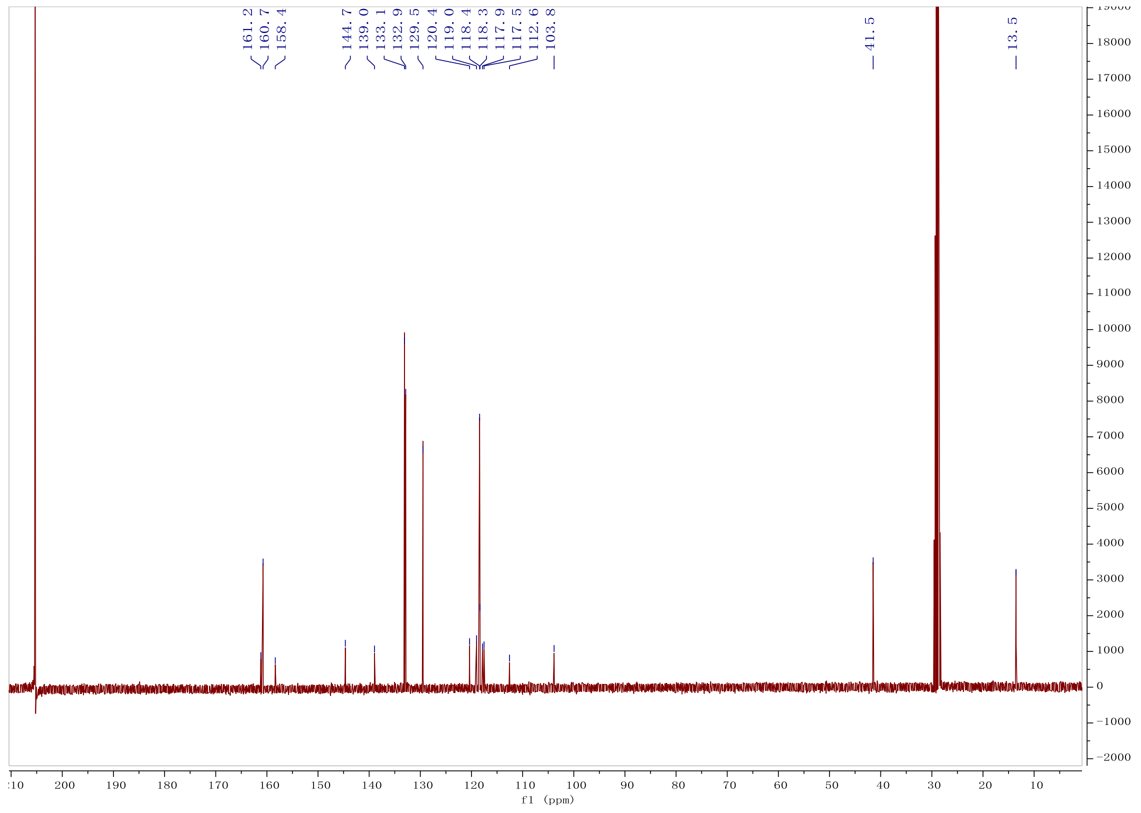

14. HRMS, HPLC, ^{1}H-NMR and ^{13}C NMR spectrum of B2

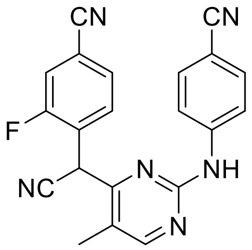

Chemical Formula: $C_{21}H_{13}FN_6$
Exact Mass: 368.1186
B2

Display Report

Analysis Info

Analysis Name	D:\Data\data\2019\20200107LTT-N-B2_RB5_01_3120.d	Acquisition Date	1/8/2020 9:17:27 AM
Method	MS-2MIN-POS.m	Operator	BDAL@DE
Sample Name	20200107LTT-N-B2	Instrument	compact 8255754.20127
Comment			

Acquisition Parameter

Source Type	ESI	Ion Polarity	Positive	Set Nebulizer	2.0 Bar
Focus	Active	Set Capillary	4500 V	Set Dry Heater	200 °C
Scan Begin	50 m/z	Set End Plate Offset	-500 V	Set Dry Gas	8.0 l/min
Scan End	3000 m/z	Set Charging Voltage	2000 V	Set Divert Valve	Waste
		Set Corona	0 nA	Set APCI Heater	0 °C

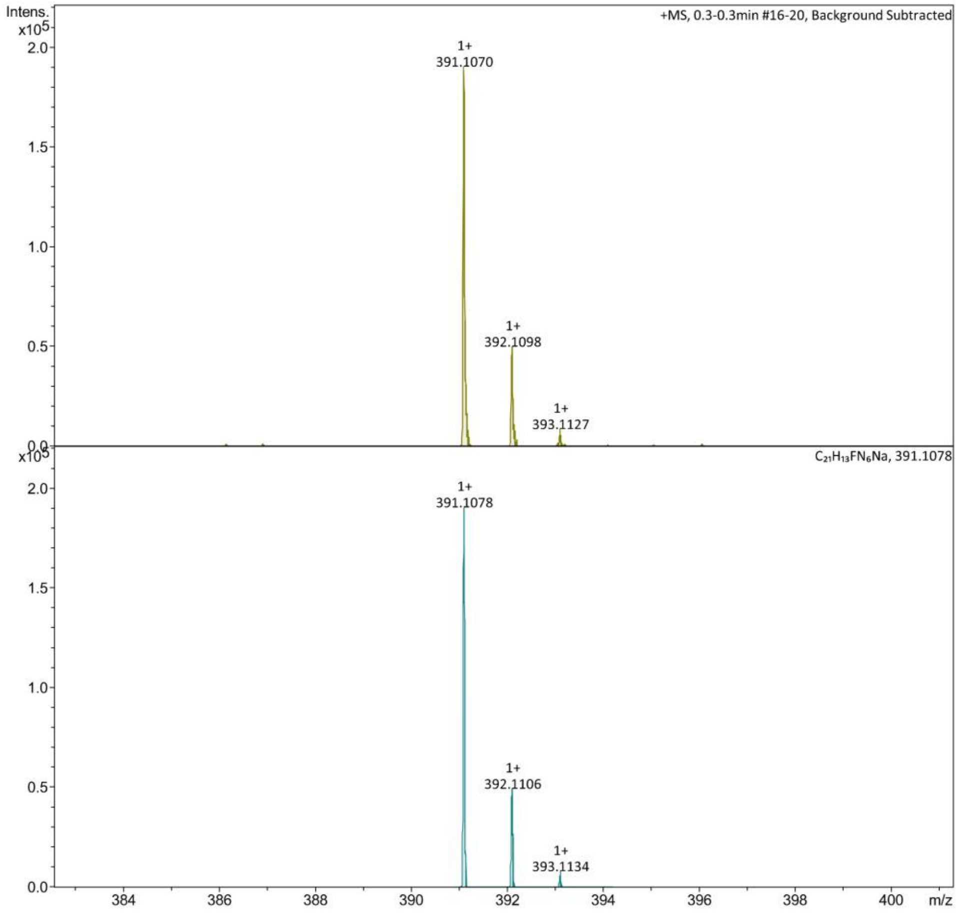

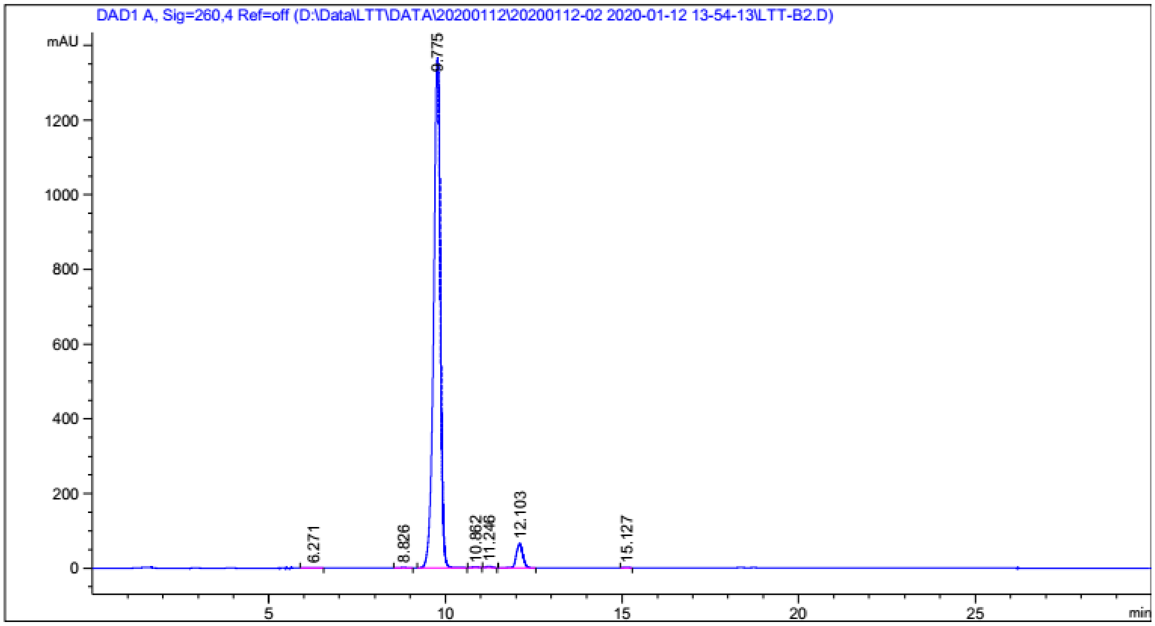

Peak #	RetTime [min]	Type	Width [min]	Area [mAU*s]	Height [mAU]	Area %
1	6.271	BB	0.1782	17.11209	1.12657	0.0897
2	8.826	BB	0.1393	17.42171	1.47485	0.0913
3	9.775	BB	0.2010	1.81387e4	1364.93164	95.0466
4	10.862	BB	0.1336	24.29423	2.17088	0.1273
5	11.246	BB	0.1360	41.53120	3.72717	0.2176
6	12.103	BB	0.1723	827.40460	66.18542	4.3356
7	15.127	BB	0.1134	17.54040	1.84565	0.0919

Totals : 1.90840e4 1441.46218

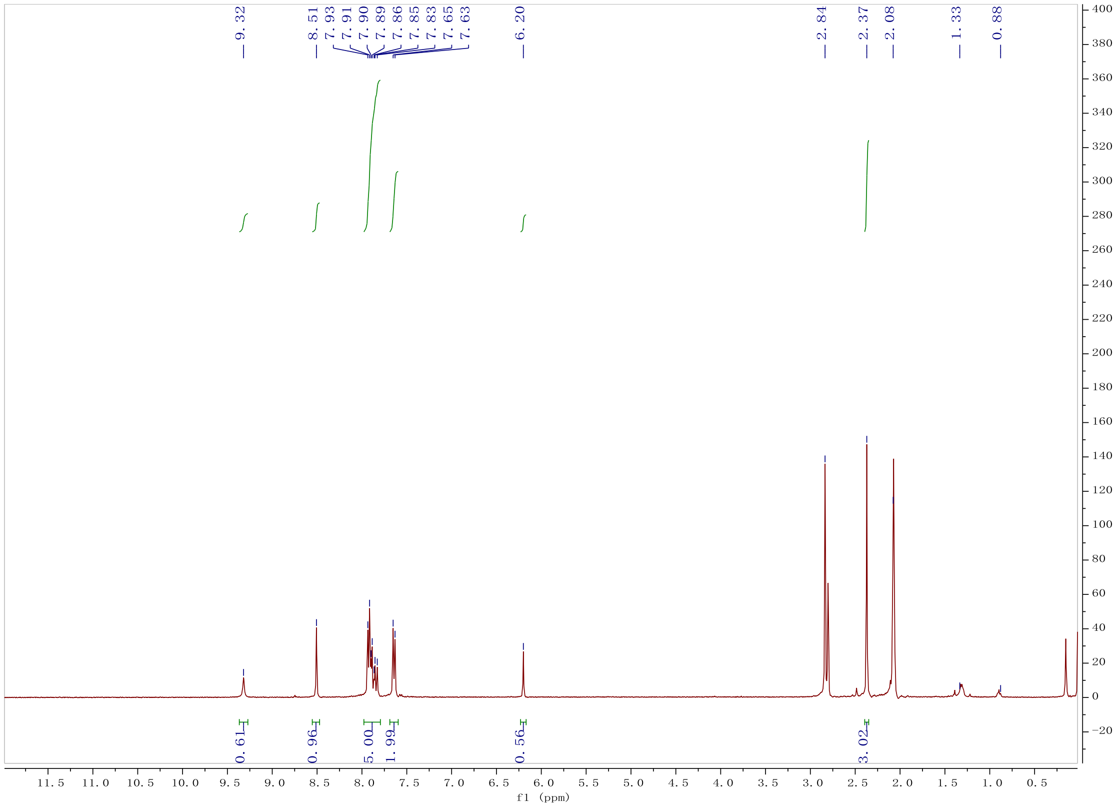

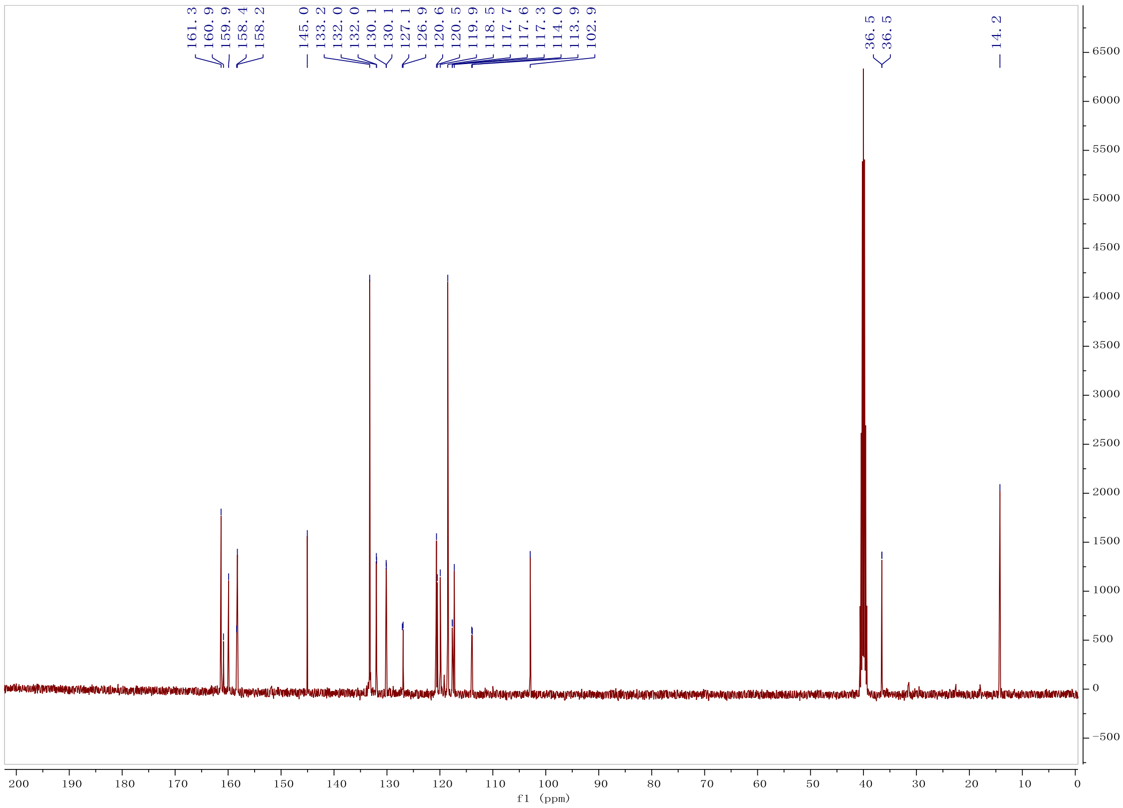

15. HRMS, HPLC, ^{1}H-NMR and ^{13}C NMR spectrum of B3

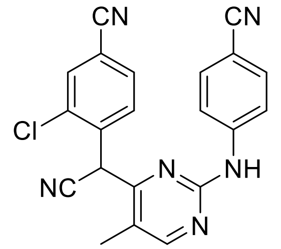

Chemical Formula: $C_{21}H_{13}ClN_6$
Exact Mass: 384.0890

B3

Display Report

Analysis Info

Analysis Name	D:\Data\data\2019\20191231LTTCH3-A3_RD7_01_3065.d	Acquisition Date	1/2/2020 19:09:25 PM
Method	MS-2MIN-POS.m	Operator	BDAL@DE
Sample Name	20191231LTTCH3-A3	Instrument	compact 8255754.20127
Comment			

Acquisition Parameter

Source Type	ESI	Ion Polarity	Positive	Set Nebulizer	2.0 Bar
Focus	Active	Set Capillary	4500 V	Set Dry Heater	200 °C
Scan Begin	50 m/z	Set End Plate Offset	-500 V	Set Dry Gas	8.0 l/min
Scan End	3000 m/z	Set Charging Voltage	2000 V	Set Divert Valve	Waste
		Set Corona	0 nA	Set APCI Heater	0 °C

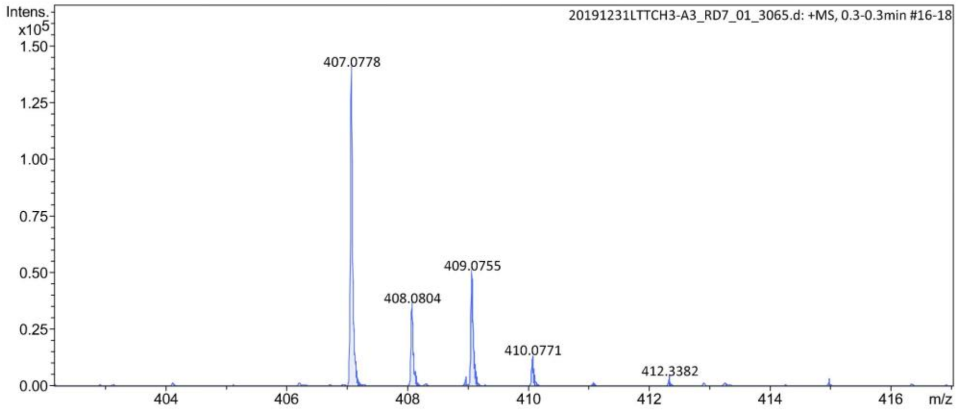

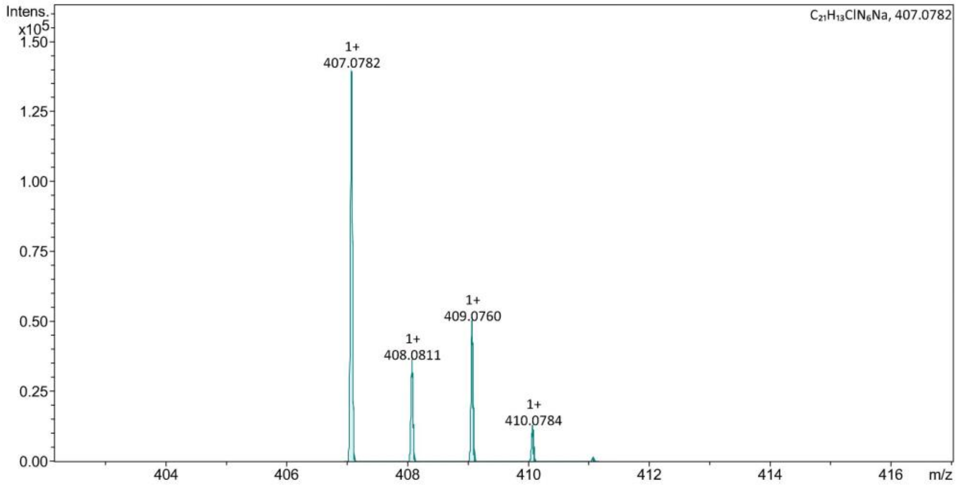

20191231LTTCH3-A3_RD7_01_3065.d
Bruker Compass DataAnalysis 4.3 printed: 1/2/2020 20:05:37 PM by: BDAL@DE Page 1 of 1

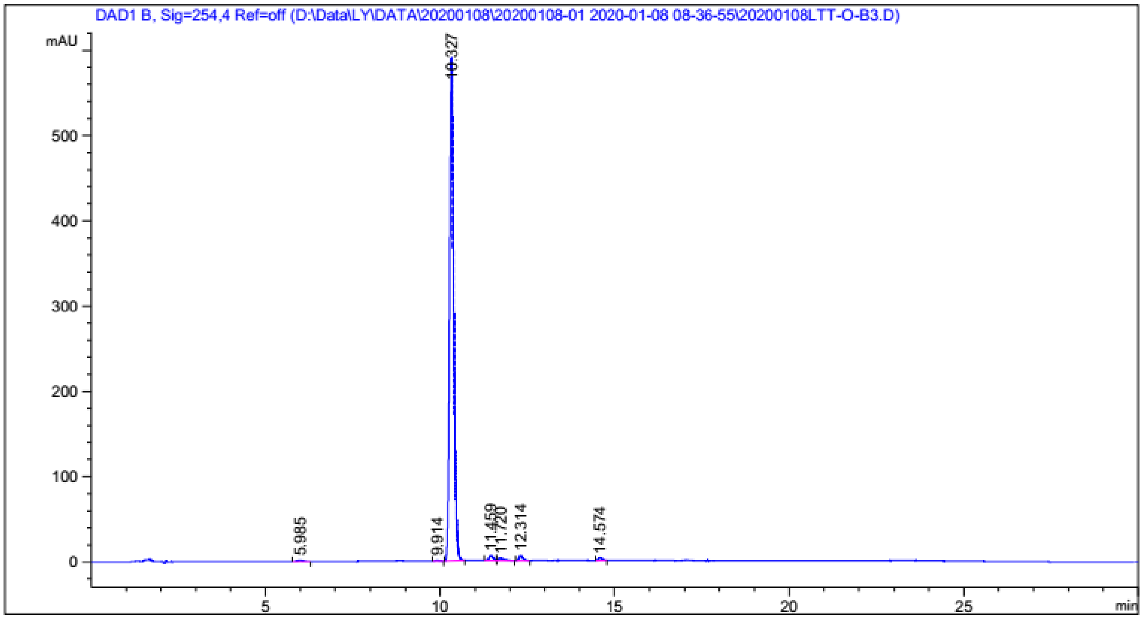

Peak #	RetTime [min]	Type	Width [min]	Area [mAU*s]	Height [mAU]	Area %
1	5.985	BB	0.1335	18.97492	1.69691	0.3859
2	9.914	BB	0.0956	8.01804	1.02083	0.1631
3	10.327	BB	0.1194	4727.72412	590.62573	96.1439
4	11.459	BV	0.1202	51.40282	6.01634	1.0453
5	11.720	VB	0.1436	35.82325	3.25032	0.7285
6	12.314	BB	0.1121	47.53342	5.99076	0.9666
7	14.574	BB	0.1022	27.86654	3.83972	0.5667

Totals : 4917.34312 612.44061

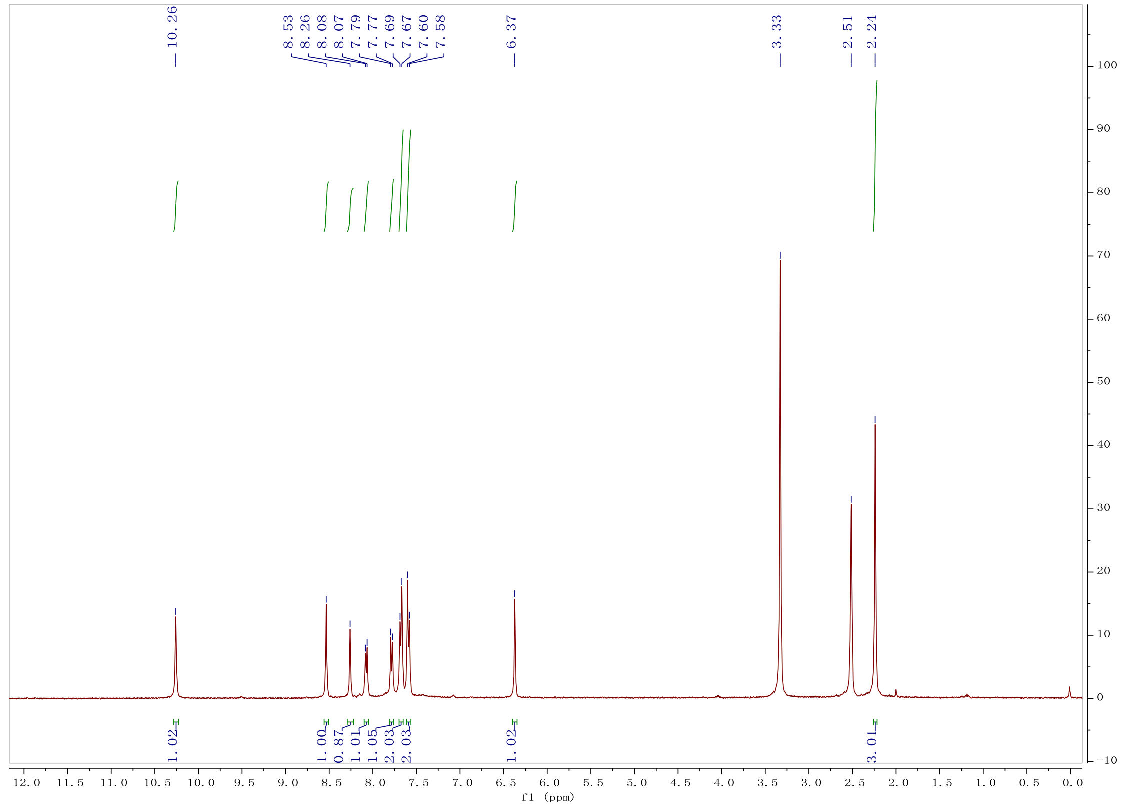
10.26
8.53
8.26
8.08
8.07
7.79
7.77
7.69
7.67
7.60
7.58
6.37
3.33
2.51
2.24
1.02
1.00
0.87
1.01
1.05
2.03
2.03
1.02
3.01
f1 (ppm)

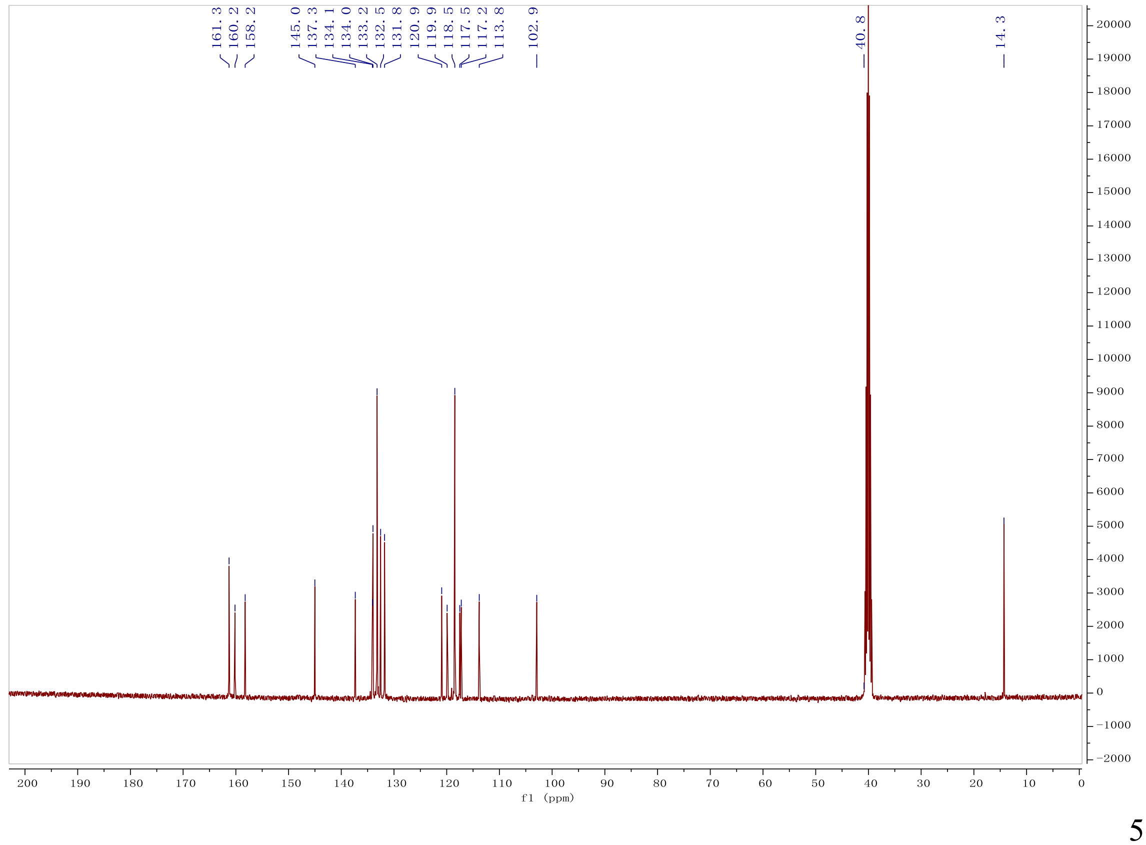
161.3
160.2
158.2
145.0
137.3
134.1
134.0
133.2
132.5
131.8
120.9
119.9
118.5
117.5
117.2
113.8
102.9
40.8
14.3
f1 (ppm)

16. HRMS, HPLC, ^{1}H-NMR and ^{13}C NMR spectrum of B4

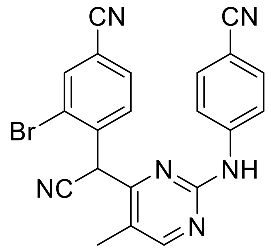

Chemical Formula: $C_{21}H_{13}BrN_6$
Exact Mass: 428.0385

B4

Display Report

Analysis Info

Analysis Name	D:\Data\data\2019\20200107LTT-P-B4_RB7_01_3122.d	Acquisition Date	1/8/2020 9:22:58 AM
Method	MS-2MIN-POS.m	Operator	BDAL@DE
Sample Name	20200107LTT-P-B4	Instrument	compact 8255754.20127
Comment			

Acquisition Parameter

Source Type	ESI	Ion Polarity	Positive	Set Nebulizer	2.0 Bar
Focus	Active	Set Capillary	4500 V	Set Dry Heater	200 °C
Scan Begin	50 m/z	Set End Plate Offset	-500 V	Set Dry Gas	8.0 l/min
Scan End	3000 m/z	Set Charging Voltage	2000 V	Set Divert Valve	Waste
		Set Corona	0 nA	Set APCI Heater	0 °C

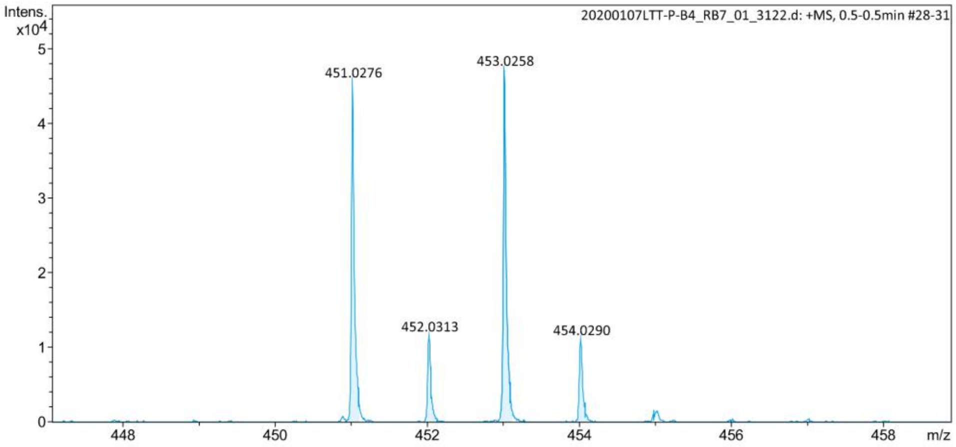

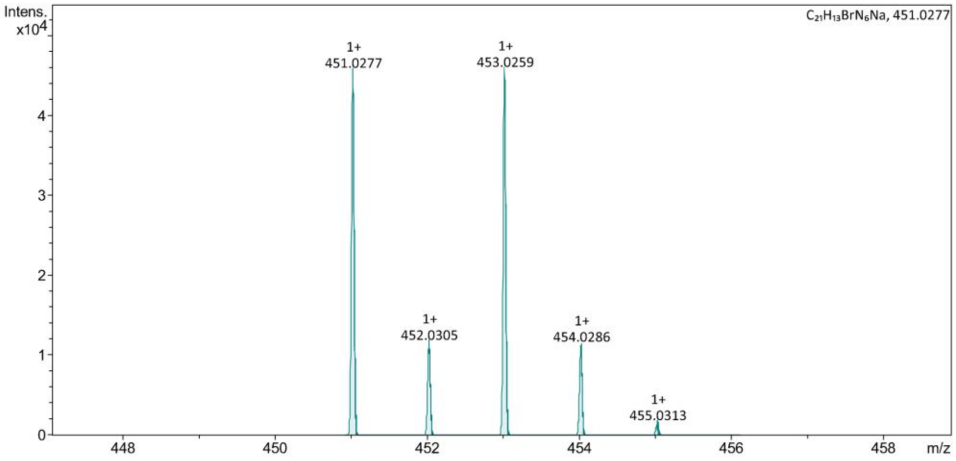

20200107LTT-P-B4_RB7_01_3122.d

Bruker Compass DataAnalysis 4.3 printed: 1/8/2020 10:27:49 AM by: BDAL@DE Page 1 of 1

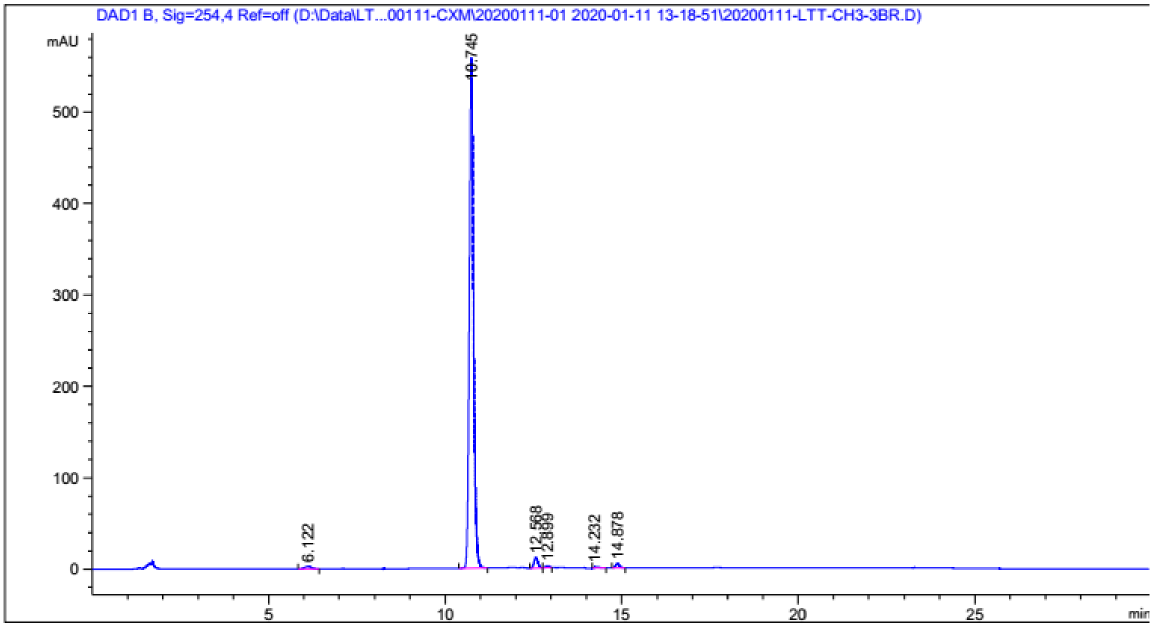

Peak #	RetTime [min]	Type	Width [min]	Area [mAU*s]	Height [mAU]	Area %
1	6.122	BB	0.1578	34.80717	2.59020	0.7215
2	10.745	BB	0.1269	4635.55518	558.28589	96.0908
3	12.568	BB	0.1109	90.43862	12.00032	1.8747
4	12.899	BB	0.0856	15.67393	2.21285	0.3249
5	14.232	BB	0.0728	10.74120	1.74553	0.2227
6	14.878	BB	0.1065	36.92433	4.92324	0.7654
Totals :				4824.14043	581.75803	

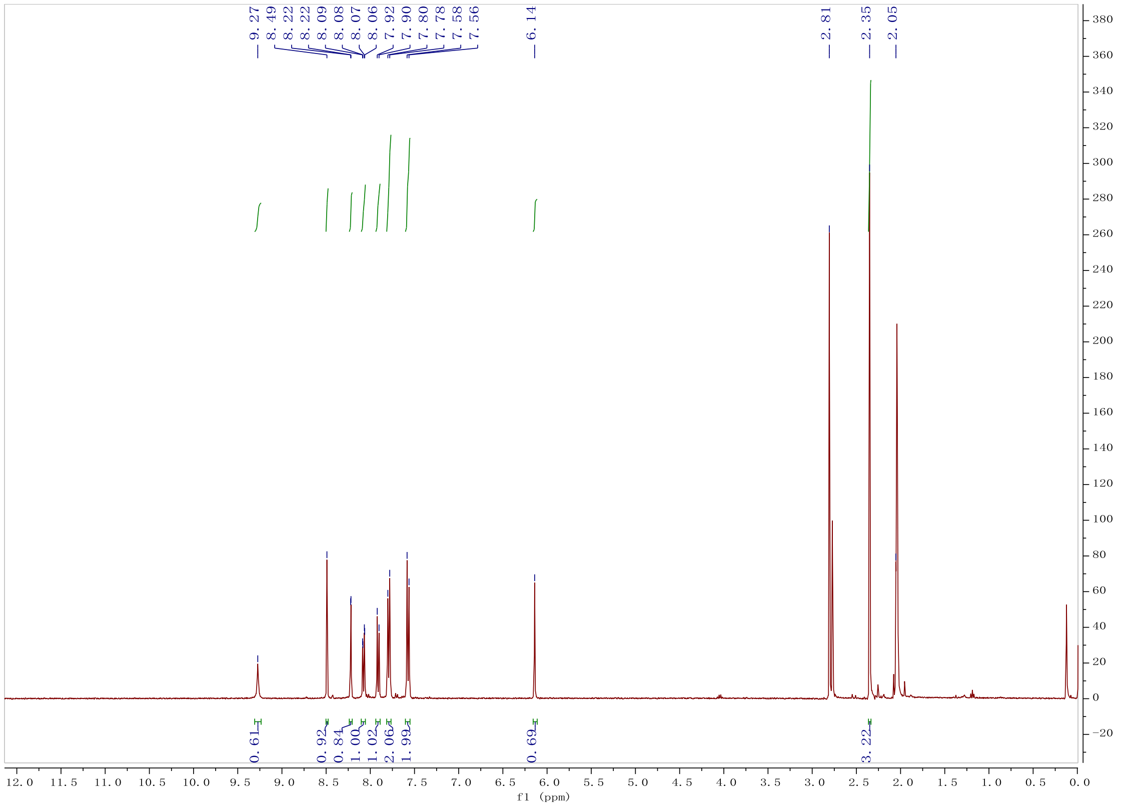
9.27
8.49
8.22
8.22
8.09
8.08
8.07
8.06
7.92
7.90
7.80
7.78
7.58
7.56
6.14
2.81
2.35
2.05
0.61
0.92
0.84
1.00
1.02
2.06
1.99
0.69
3.22
f1 (ppm)

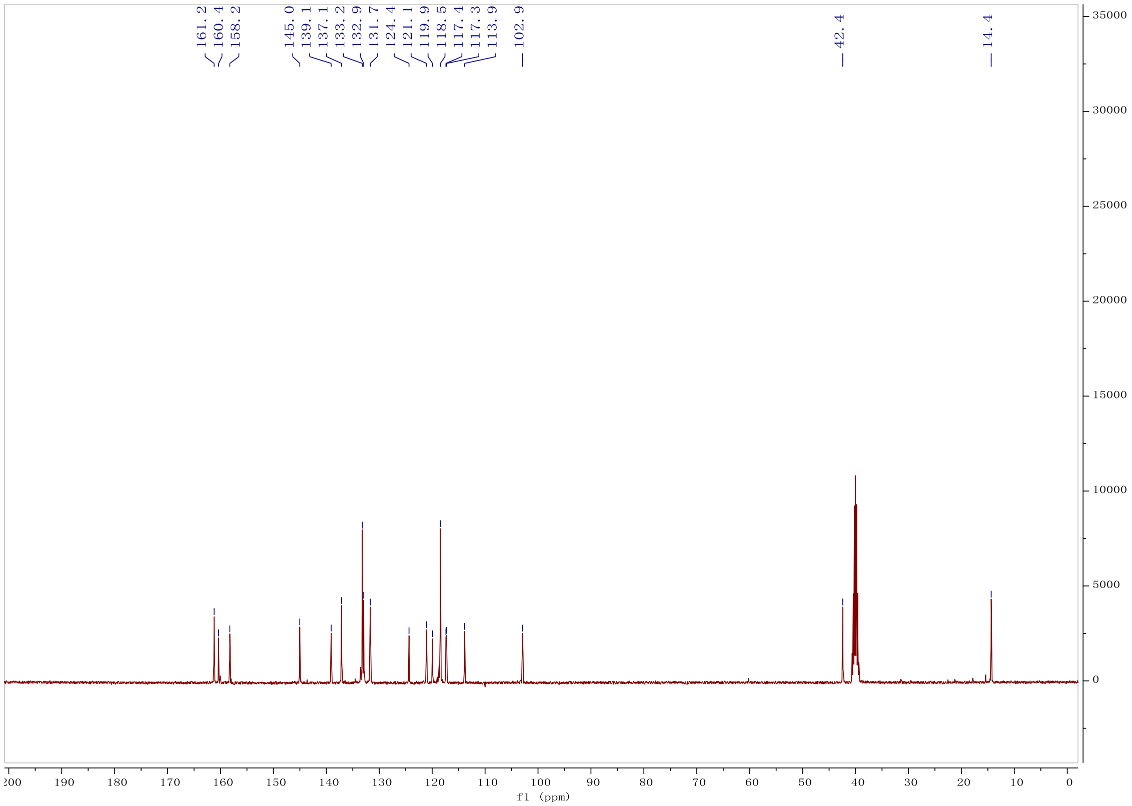
161.2
160.4
158.2
145.0
139.1
137.1
133.2
132.9
131.7
124.4
121.1
119.9
118.5
117.4
117.3
113.9
102.9
42.4
14.4
f1 (ppm)

17. HRMS, HPLC, ^{1}H-NMR and ^{13}C NMR spectrum of B5

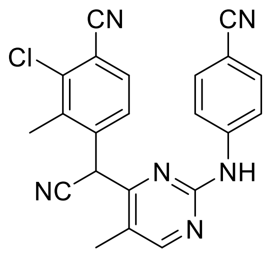

Chemical Formula: $C_{22}H_{15}ClN_6$
Exact Mass: 398.1047

B5

Display Report

Analysis Info

Analysis Name	D:\Data\data\2019\20200107LTT-Q-B5_RA3_01_3126.d	Acquisition Date	1/8/2020 9:34:01 AM
Method	MS-2MIN-POS.m	Operator	BDAL@DE
Sample Name	20200107LTT-Q-B5	Instrument	compact 8255754.20127
Comment			

Acquisition Parameter

Source Type	ESI	Ion Polarity	Positive	Set Nebulizer	2.0 Bar
Focus	Active	Set Capillary	4500 V	Set Dry Heater	200 °C
Scan Begin	50 m/z	Set End Plate Offset	-500 V	Set Dry Gas	8.0 l/min
Scan End	3000 m/z	Set Charging Voltage	2000 V	Set Divert Valve	Waste
		Set Corona	0 nA	Set APCI Heater	0 °C

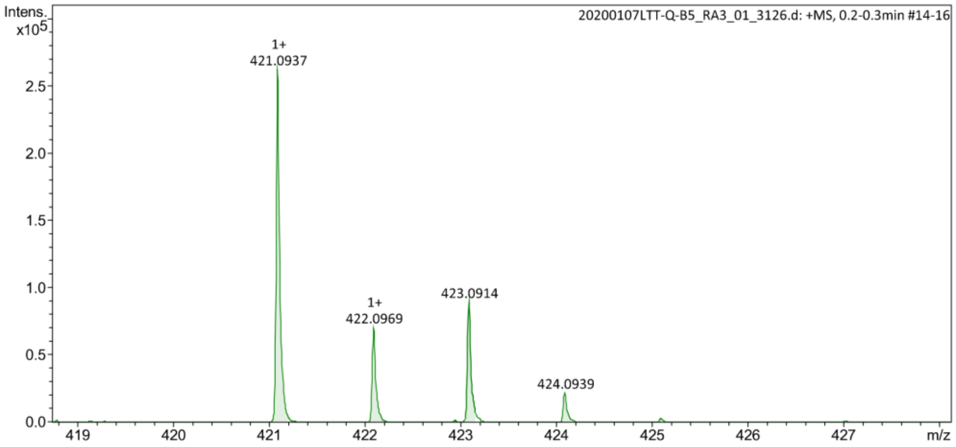

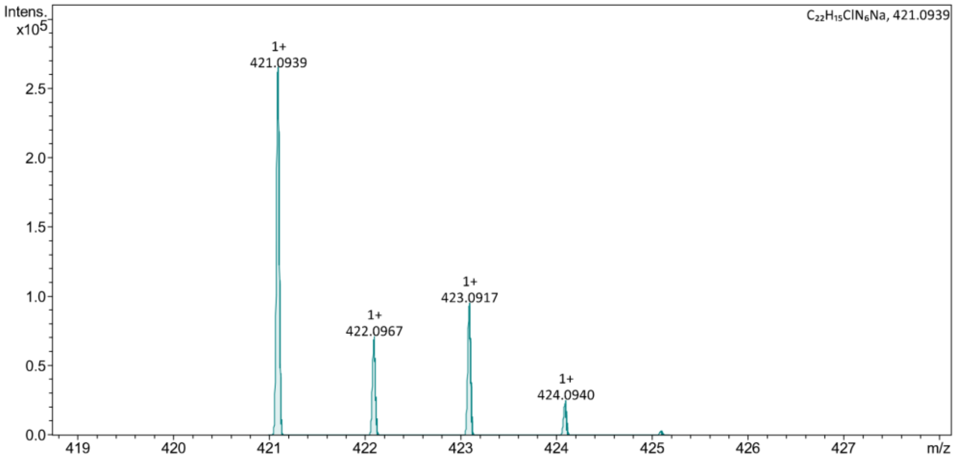

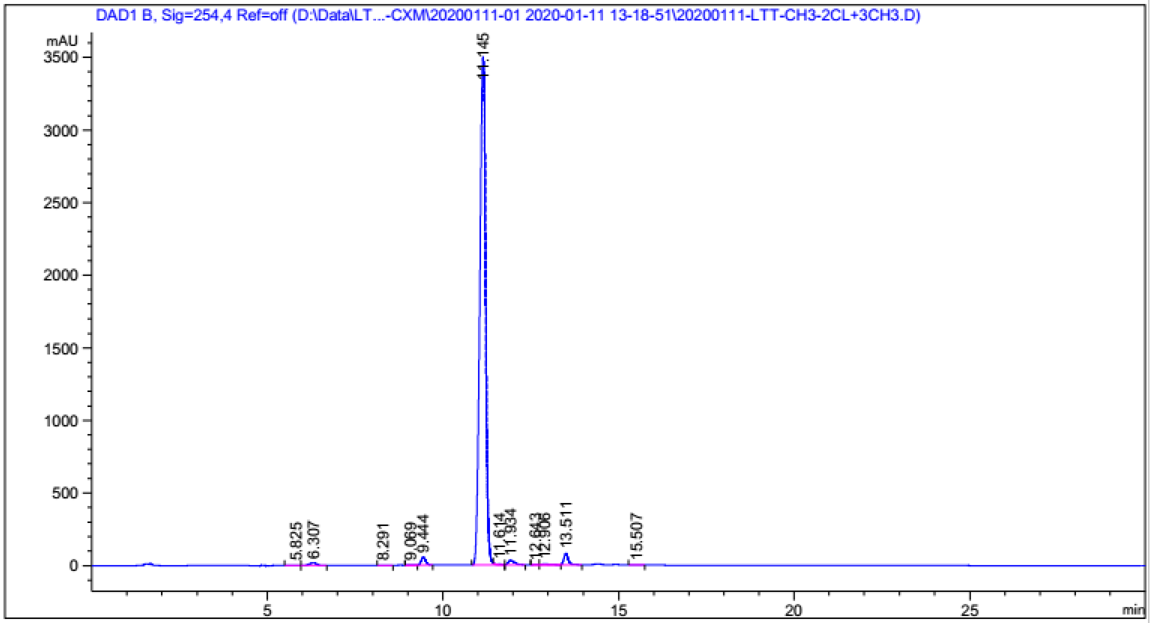

Peak #	RetTime [min]	Type	Width [min]	Area [mAU*s]	Height [mAU]	Area %
1	5.825	BB	0.0978	8.39060	1.02702	0.0198
2	6.307	BB	0.1969	268.34116	19.21711	0.6347
3	8.291	BB	0.1510	14.35273	1.14338	0.0339
4	9.069	VV E	0.1184	29.35218	2.96779	0.0694
5	9.444	VB R	0.1384	486.48398	54.37025	1.1506
6	11.145	BV R	0.1373	4.02295e4	3493.60571	95.1467
7	11.614	VV E	0.1148	42.02334	4.59091	0.0994
8	11.934	VB	0.1698	377.39883	31.83869	0.8926
9	12.643	BV E	0.1032	14.59579	1.68243	0.0345
10	12.906	VV E	0.1925	99.51124	6.78839	0.2354
11	13.511	VB R	0.1325	693.26202	80.08997	1.6396
12	15.507	BB	0.1475	18.35076	1.48132	0.0434

Totals : 4.22815e4 3698.80296

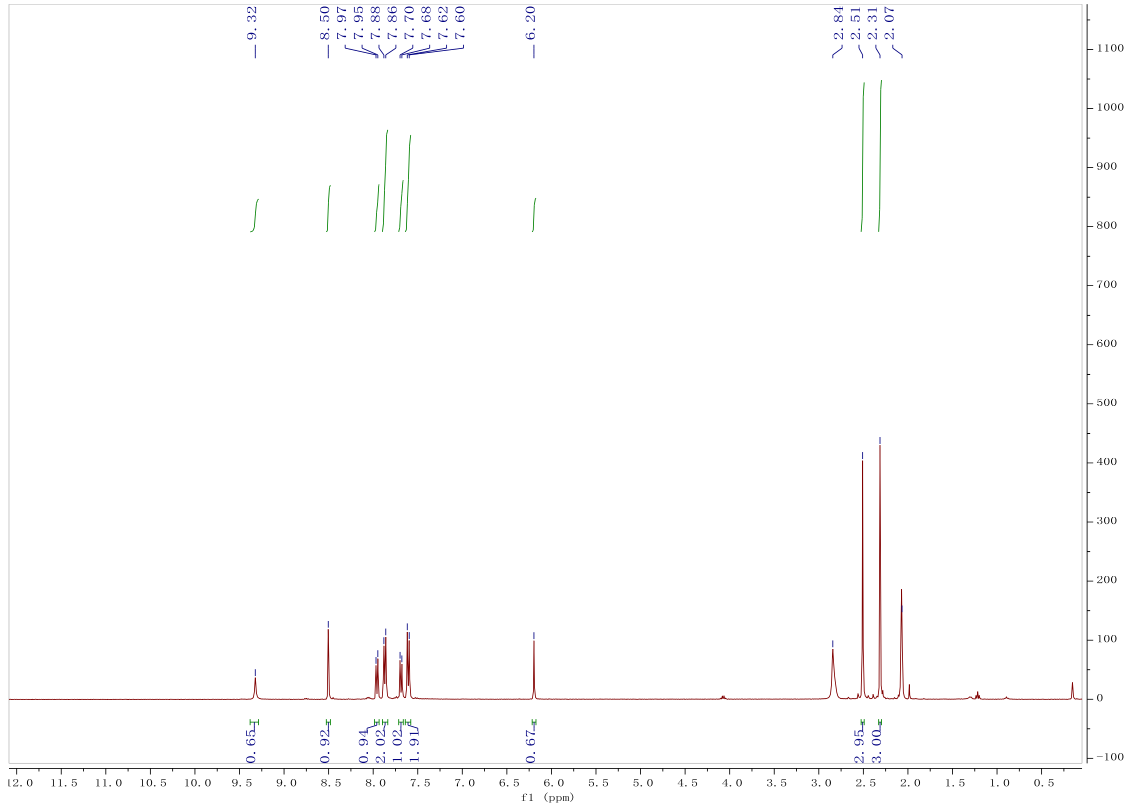

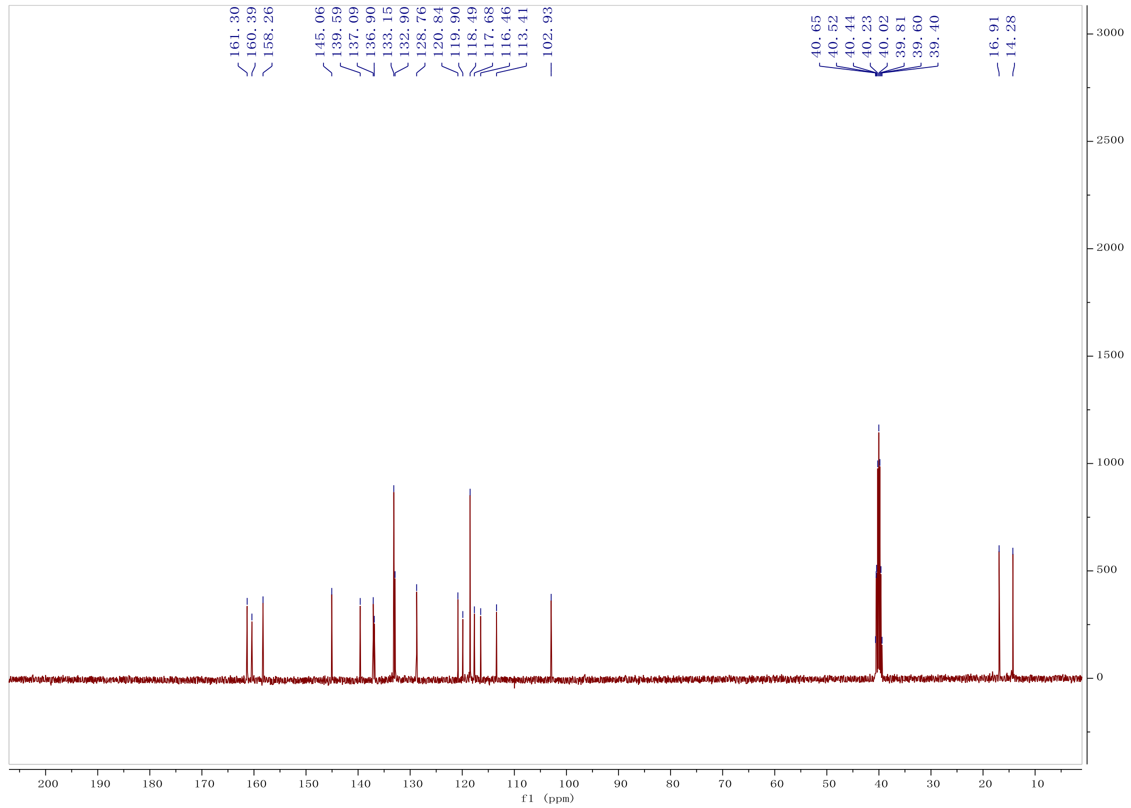

18. HRMS, ^{1}H-NMR and ^{13}C NMR spectrum of B6

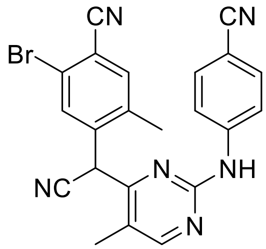

Chemical Formula: $C_{22}H_{15}BrN_6$
Exact Mass: 442.0542
B6

Display Report

Analysis Info

Analysis Name	D:\Data\data\2019\20191231LTTCH3-A8_RE5_01_3059.d	Acquisition Date	1/2/2020 18:52:50 PM
Method	MS-2MIN-POS.m	Operator	BDAL@DE
Sample Name	20191231LTTCH3-A8	Instrument	compact 8255754.20127
Comment			

Acquisition Parameter

Source Type	ESI	Ion Polarity	Positive	Set Nebulizer	2.0 Bar
Focus	Active	Set Capillary	4500 V	Set Dry Heater	200 °C
Scan Begin	50 m/z	Set End Plate Offset	-500 V	Set Dry Gas	8.0 l/min
Scan End	3000 m/z	Set Charging Voltage	2000 V	Set Divert Valve	Waste
		Set Corona	0 nA	Set APCI Heater	0 °C

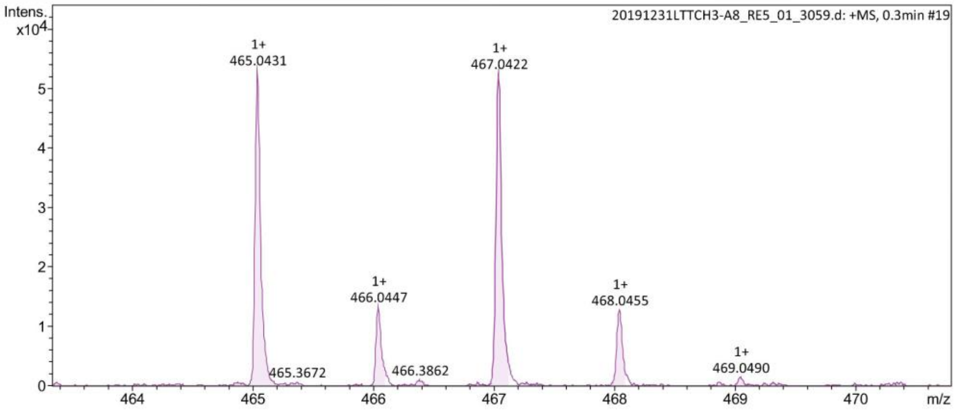

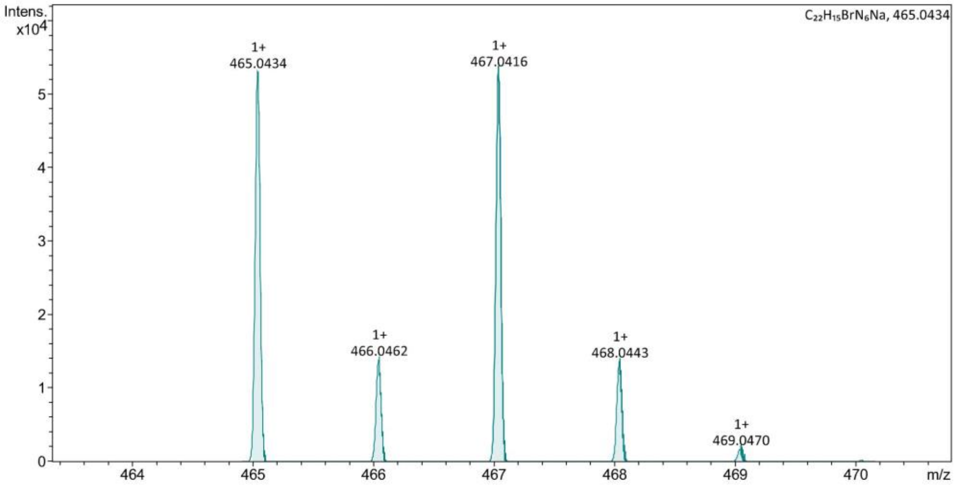

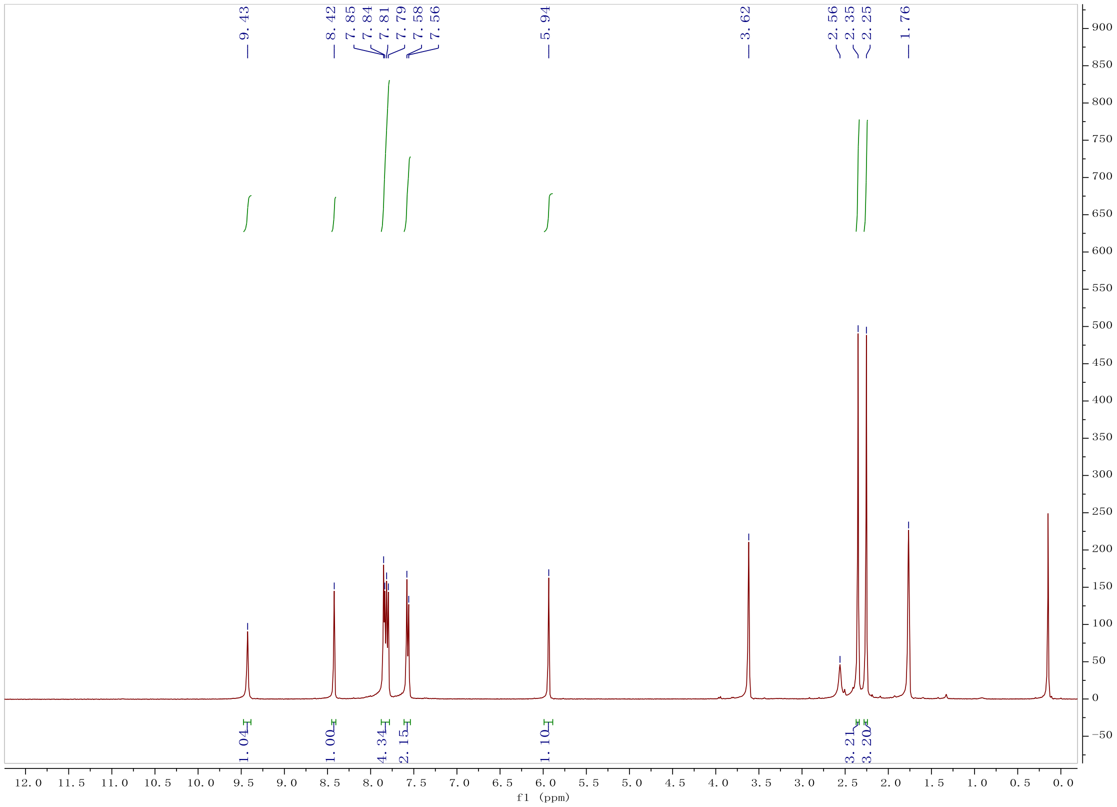

9.43
8.42
7.85
7.84
7.81
7.79
7.58
7.56
5.94
3.62
2.56
2.35
2.25
1.76
1.04
1.00
4.34
2.15
1.10
3.21
3.20
f1 (ppm)

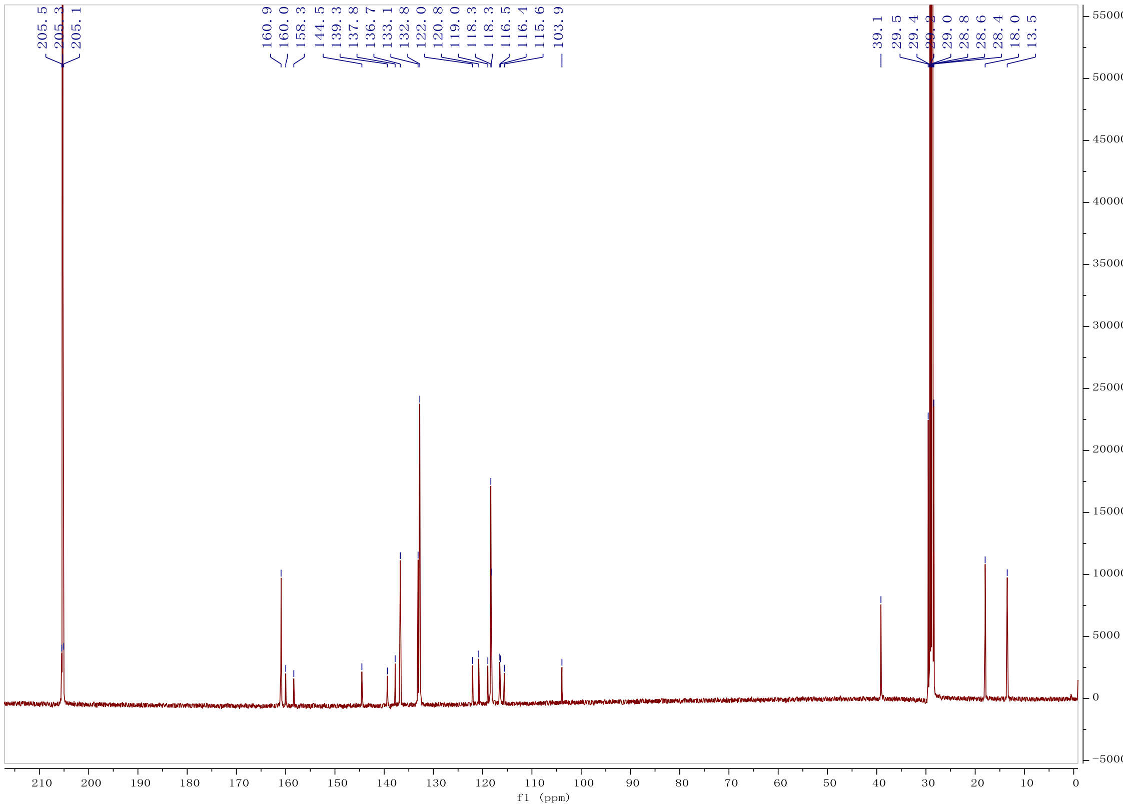

205.5
205.3
205.1
160.9
160.0
158.3
144.5
139.3
137.8
136.7
133.1
132.8
122.0
120.8
119.0
118.3
118.3
116.5
116.4
115.6
103.9
39.1
29.5
29.4
29.2
29.0
28.8
28.6
28.4
18.0
13.5
f1 (ppm)
